# Improving sleep quality leads to better mental health: A meta-analysis of randomised controlled trials

**DOI:** 10.1016/j.smrv.2021.101556

**Published:** 2021-12

**Authors:** Alexander J. Scott, Thomas L. Webb, Marrissa Martyn-St James, Georgina Rowse, Scott Weich

**Affiliations:** a Keele University, School of Psychology, Keele, UK; b School of Health and Related Research (ScHARR), The University of Sheffield, UK; c Department of Psychology, The University of Sheffield, UK; d Clinical Psychology Unit, Department of Psychology, The University of Sheffield, UK

**Keywords:** Sleep, Insomnia, CBTi, Mental health, Meta-analysis, Causal inference, Depression, Anxiety, Stress, Psychosis

## Abstract

The extent to which sleep is causally related to mental health is unclear. One way to test the causal link is to evaluate the extent to which interventions that improve sleep quality also improve mental health. We conducted a meta-analysis of randomised controlled trials that reported the effects of an intervention that improved sleep on composite mental health, as well as on seven specific mental health difficulties. 65 trials comprising 72 interventions and *N* = 8608 participants were included. Improving sleep led to a significant medium-sized effect on composite mental health (*g+* = −0.53), depression (*g+* = −0.63), anxiety (*g+* = −0.51), and rumination (*g+* = −0.49), as well as significant small-to-medium sized effects on stress (*g+* = −0.42), and finally small significant effects on positive psychosis symptoms (*g+* = −0.26). We also found a dose response relationship, in that greater improvements in sleep quality led to greater improvements in mental health. Our findings suggest that sleep is causally related to the experience of mental health difficulties. Future research might consider how interventions that improve sleep could be incorporated into mental health services, as well as the mechanisms of action that explain how sleep exerts an effect on mental health.

## Does improving sleep lead to better mental health? A meta-analysis of randomised controlled trials

Problems sleeping are common. A review of several hundred epidemiological studies [1] concluded that nearly one-third of the general population experience symptoms of insomnia (defined as difficulties falling asleep and/or staying asleep), between 4% and 26% experience excessive sleepiness, and between 2% and 4% experience obstructive sleep apnoea. Additionally, a recent study of over 2000 participants reported that the prevalence of ‘general sleep disturbances’ was 32% [2] and Chattu et al. concluded on the basis of a large systematic review of the evidence that public and health professionals need to be more aware of the adverse effects of poor sleep [3]. Mental health problems are also common, with around 17% of adults experiencing mental health difficulties of varying severities [4], and evidence from large nationally representative studies suggesting that mental health difficulties are on the increase [5]. Sleep and mental health are, therefore, global public health challenges in their own right, with each having substantive impacts on both individuals and society [3,6,7]. However, problems sleeping and mental health difficulties are also intrinsically linked [8,9]. It was previously assumed that mental health difficulties led to problems sleeping [10,11]; however, the reverse may also be true [12], such that poor sleep contributes to the onset, recurrence, and maintenance of mental health difficulties [[13]∗, [14], [15]∗, [16], [17]]. Therefore, the extent to which there is a causal relation between (poor) sleep and (worse) mental health and the possibility that interventions designed to improve sleep might be able to reduce mental health difficulties warrants investigation.

### Evidence on the relationship between sleep and mental health

The association between sleep and mental health is well documented [9,13,[18], [19], [20], [21], [22], [23]∗]. For example, people with insomnia are 10 and 17 times more likely than those without insomnia to experience clinically significant levels of depression and anxiety, respectively [24]. Furthermore, a meta-analysis of 21 longitudinal studies reported that people with insomnia at baseline had a two-fold risk of developing depression at follow-up compared with people who did not experience insomnia [13]. Although research most commonly studies the associations between insomnia and depression and anxiety, there is also evidence that problems sleeping are associated with a variety of mental health difficulties. For example, poor sleep has also been associated with post-traumatic stress [25], eating disorders [26], and psychosis spectrum experiences such as delusions and hallucinations [23,27]. Studies have also found that specific sleep disorders, such as sleep apnoea [28], circadian rhythm disruption [29], restless leg syndrome [30], excessive daytime sleepiness and narcolepsy [31,32], sleepwalking [33], and nightmares [34] are all more prevalent in those experiencing mental health difficulties.

Unfortunately, most research on the association between sleep and mental health is observational in design. While informative, inferring causation from such studies is difficult. For example, cross-sectional designs tell us that variables are associated in some way, but they cannot say whether one variable precedes the other in a causal chain [35]. Longitudinal designs provide stronger evidence, but are prone to residual confounding [[36], [37], [38]] and other forms of bias that limit causal inference [[39], [40], [41], [42], [43]]. The best evidence is provided by studies that randomly allocate participants to experimental and control conditions to minimise the effects of potential confounds [44,45]. Therefore, to establish whether sleeping problems are causally associated with mental health difficulties, it is necessary to experimentally manipulate sleep to see whether changes in sleep lead to changes in mental health over time (i.e., the interventionist approach to causation, [46]).

Many RCTs have examined the effect of interventions designed to improve sleep (typically cognitive behavioural therapy for insomnia, CBTi), on mental health (typically depression and anxiety). There have also been attempts to meta-analyse some of these RCTs and quantify their effects on mental health outcomes [[47]∗, [48], [49], [50]]. However, even these meta-analyses do not permit robust conclusions as to the causal impact of sleep on mental health outcomes for several reasons. First, previous reviews have included studies that did not successfully manipulate sleep (i.e., the intervention did not improve sleep relative to controls). It is not possible to conclude whether sleep is causally linked to mental health if the experimental manipulation of sleep is unsuccessful [51]. Indeed, these studies simply tell us that it can sometimes be difficult to improve sleep in the first place. Second, reviews have tended to examine the effect of interventions targeting sleep on mental health at the first post-intervention time point. This is problematic for two reasons; 1) there is no temporal lag between the measurement of sleep and measurement of mental health (a key tenet of causal inference); and 2) effects are limited to the short-term where they are likely to be strongest. Third, the focus of previous reviews has been limited to depression and anxiety only, and typically limited to CBTi interventions. Therefore, the effect of improving sleep on other mental health outcomes, using different approaches to intervention, is limited. Finally, to date there has been no or limited attempts to investigate variables that influence – or moderate – the impact of interventions that improve sleep on mental health. It is crucial that the impact of such variables is systematically examined to understand whether the effect of improving sleep on mental health differs across populations, settings, and study designs.

### The present review: an interventionist approach to causation

The present review sought to address these issues to provide an accurate and robust estimate of the effect of changes in sleep quality (i.e., as a result of an intervention) on changes in mental health. To test this empirically, we identified randomised controlled trials that successfully manipulated sleep in an intervention group relative to controls, and then measured mental health at a later follow-up point. We did not limit the scope of interventions to CBTi, or the measures of mental health to solely depression and/or anxiety. Instead, we included any intervention designed to improve sleep that produced a statistically significant effect on sleep quality relative to controls and examined the effect of that improvement in sleep on any subsequent mental health outcome. To better isolate the effect of improved sleep on mental health, we excluded interventions that included specific elements targeting mental health (e.g., CBT elements for depression). Given the (potentially) high degree of heterogeneity between studies that this approach might create, we examined the effect of different study characteristics and outcomes using moderation analyses. Our primary hypothesis is that interventions that significantly improve sleep will lead to significantly improved mental health at follow-up.

## Method

### Eligibility criteria

To be included in the present review, studies needed to 1) be a randomized controlled trial that tested an intervention designed to improve sleep; 2) produce a statistically significant effect on sleep quality when compared to a control group or an alternative treatment, 3) report a measure of mental health subsequent to the measure of sleep quality, 4) report sufficient data to compute an effect size representing the impact of the intervention on both sleep quality and mental health, 5) be written in English, or translatable using available resources. In order to reliably and validly assess the independent contribution of changes in sleep on mental health outcomes among adult populations, studies were excluded if 1) the intervention contained elements that specifically target a mental health problem in addition to elements that target sleep; or 2) recruited children and young people (i.e., <18 years of age).

### Search strategy

First, we searched MEDLINE (1946 to present), Embase (1974 to present), PsycINFO (1967 to present), and The Cochrane Library (1898 to present) using the Cochrane Highly Sensitive Search Strategy (i.e., HSSS, [52]) to identify RCTs that included terms relating to sleep quality and/or sleep disorders, and mental health (see Table 1 for a list of the search terms and Supplementary Material 1 for an example search strategy). Second, the reference lists of extant reviews of the relationship between sleep and mental health were searched for any potential articles. Third, a search for any unpublished or ongoing studies was conducted by searching online databases including White Rose Online, The National Research Register, WHO approved clinical trial databases (e.g., ISRCTN), and PROSPERO. Searches were originally conducted in May 2019 and then updated in February 2021.

**Table 1:** Search terms used to identify RCT's that examined the effect of improving sleep on mental health.

HSSS for RCTs	Sleep terms	Mental health terms
Randomi$ed controlled trial	Sleep∗	“Psychological health”
Controlled clinical trial	“Circadian rhythm”	“Mental”
Randomi$ed	Insomnia	Psychiat∗
Placebo	Hypersomnia	Affect∗
Drug therapy	Parasomnia	Depress∗
Randomly	Narcolepsy	Mood
Trial	Apn$ea	Stress
Groups	Nightmare∗	Anxi∗
	“Restless leg∗ syndrome”	Phobi∗
		“Obsessive compulsive disorder”
		OCD
		PTSD
		“Post-traumatic stress disorder”
		Psychos∗
		Psychotic
		Schiz∗
		Bipolar
		Hallucination∗
		Delusion∗
		“Eating disturbance∗”
		Anorexia
		Bulimia
		“Binge eating”

*Notes*: HSSS for RCTs = highly sensitive search strategy for randomised controlled trials, OCD = obsessive compulsive disorder, PTSD = post-traumatic stress disorder.

### Data management and study selection

We followed PRISMA guidelines [53] when selecting studies. The first phase of screening removed duplicate records and records that were clearly ineligible based on the title and/or abstract. The second phase of screening cross-referenced full-text versions of articles against the inclusion criteria, with eligible records included in the present review, and ineligible records excluded along with reasons for exclusion. Records were screened by two members of the review team, and a sub-sample of 10% of each reviewer's records were second checked by the other reviewer, with almost perfect agreement between the reviewers (*kappa* = 1.00 and 0.99).

### Data extraction

Data was extracted from included studies using a standardized form and an accompanying manual detailing each variable for extraction. In addition to extracting statistical data to compute effect sizes, data pertaining to source characteristics of included studies (e.g., publication status, year, impact factor), characteristics of the sample (e.g., age, type of mental health problem), the study (e.g., the nature of the comparison group, length of follow-up), and the intervention (e.g., intervention type, mode of delivery) was also extracted.

### Outcomes and prioritization

#### Measuring improvements in sleep

The concept of ‘improved sleep’ is multifaceted and can mean different things to different people [[54], [55], [56]]. Consequently, one challenge for the proposed review was to ensure that included studies assessed a similar notion of improved sleep so that they could be meaningfully combined using a single metric. Therefore, we specified that primary studies reported a measure that reflected the overall quality of sleep experienced by participants. The concept of sleep quality can also be subjective [54]; however, broadly speaking, sleep quality consists of sleep continuity (e.g., sleep onset, sleep maintenance, and number of awakenings) and daytime impact (e.g., the extent to which the person feels refreshed on waking and throughout the day, see [54,57]). We used the following hierarchy to decide which outcome measure(s) to use to estimate an effect size (in descending order of prioritization); 1) self-report measures of global sleep quality (e.g., the Pittsburgh Sleep Quality Index); 2) outcomes specific to a given sleep disorder that assess sleep continuity and impact on daily life (e.g., the Insomnia Severity Index); and 3) individual components of self-reported sleep continuity aggregated to form a single composite effect size (e.g., the average effect of intervention on sleep onset latency (SoL) and wake after sleep onset (WASO)).

#### Measuring mental health

We examined the effect of improving sleep on 1) composite mental health (which included all mental health outcomes reported across studies, see Table 2 for outcomes), and 2) specific mental health difficulties in isolation (e.g., depression separately from other mental health outcomes). We computed the between-group effect of improving sleep on each mental health outcome reported by the study at the furthest follow-up point available. This strategy provides a stringent test of the effect of improving sleep on mental health outcomes in the sense that any changes need to have been maintained over time. In line with previous reviews [58], these effect sizes were then averaged to form a ‘composite’ measure of mental health. As with the measures of sleep quality, we prioritized self-report measures of mental health rather than observer-rated measures, as arguably it is the subjective experience of mental health problems that is most important [59].

**Table 2:** Summary of studies included in the review.

Author (year)	Intervention	Control	Outcome	Measure	*n*_*e*_	*n*_*c*_	*g*_*+*_
Alessi et al. (2016) [117]	CBTi	Sleep education	Depression	PHQ-9	89	51	0.20
Ashworth et al. (2015) [118]	CBTi	CBTi (self-help)	Anxiety	DASS-A	18	18	−1.41∗∗∗
			Depression	BDI	18	18	−2.31∗∗∗
Behrendt et al. (2020) [119]	CBTi	WLC	Depression	CES-D	46	80	−0.52∗∗
			Rumination	PSWQ	46	80	−0.45∗
Bergdahl et al. (2016) [120]	CBTi	Acupuncture	Anxiety	HADS-A	23	22	0.03
			Depression	HADS-D	23	22	0.06
Blom et al. (2017) [121]	CBTi	CBT for depression	Depression	MADRS	20	17	−0.31
Cape et al. (2016) [122]	CBTi	TaU	Anxiety	GAD-7	91	99	−0.11
			Depression	PHQ-9	92	100	−0.20
Casault et al. (2015) [123]	CBTi	WLC	Anxiety	HADS-A	17	18	−0.39
			Depression	HADS-D	17	18	−0.11
Chang et al. (2016) [124]	Herbal tea	WLC	Depression	EPDS	35	37	−0.52∗
Chang et al. (2016) [125]	Sleep education + relaxation	WLC	Anxiety	HADS-A	43	41	−0.68∗∗
			Depression	HADS-D	43	41	−0.52∗
Chao et al. (2021) [126]	CBTi	WLC	Depression	HADS-D	32	39	−0.67∗∗
			Anxiety	HADS-A	32	39	−0.60∗
Chen et al. (2009) [127]	Yoga	TaU	Depression	TDS	62	66	−0.60∗∗∗
Chen et al. (2019) [128]	Acupuncture	Sham acupuncture	Mood/affect	K-10	31	31	−0.50
Cheng et al. (2019) [129]	CBTi	Sleep education	Depression	QIDS	358	300	−0.45∗∗∗
Christensen et al. (2016) [130]	CBTi	Health education	Anxiety	GAD-7	224	280	−0.34∗∗∗
			Depression	PHQ-9	224	280	−0.53∗∗∗
Chung et al. (2018) [131]	Acupuncture	WLC	Anxiety	HADS-A	71	32	−0.37
			Depression	HADS-D	71	32	−0.46∗
Currie et al. (2000) [132]	CBTi	WLC	Depression	BDI	32	28	−0.31
Edinger et al. (2005)^a^ [133]	CBTi	TaU	Mood/affect	POMS	6	7	−1.27
Edinger et al. (2005)^b^ [133]	Sleep hygiene	TaU	Mood/affect	POMS	7	7	−1.00
Espie et al. (2008) [81]	CBTi	Sleep hygiene	Anxiety	HADS-A	67	39	−0.52∗
			Depression	HADS-D	67	39	−0.59∗∗
Espie et al. (2014) [134]	CBTi	TaU	Anxiety	DASS-A	40	47	−0.79∗∗∗
			Depression	DASS-D	40	47	−0.94∗∗∗
			Stress	DASS-S	40	47	−0.93∗∗∗
Espie et al. (2019) [135]	CBTi	WLC	Anxiety	GAD-7	411	495	−0.31∗∗∗
			Depression	PHQ-9	411	495	−0.39∗∗∗
Falloon et al. (2015) [136]	Sleep restriction	Sleep hygiene	Anxiety	GAD-7	43	50	−0.50∗
			Depression	PHQ-9	43	50	−0.27
Felder et al. (2020) [137]	CBTi	TaU	Depression	EPDS	88	91	−0.40∗∗
			Anxiety	GAD-7	88	90	−0.37∗
Freeman et al. (2015) [138]	CBTi	TaU	Delusions	PSYRATS	23	25	−0.24
			Hallucinations	PSYRATS	23	25	−0.23
			Paranoia	GPTS	20	25	−0.28
			Psychosis	PANSS tot	21	24	−0.07
Freeman et al. (2017) [139]	CBTi	TaU	Anxiety	GAD-7	603	971	−0.26∗∗∗
			Depression	PHQ-9	603	971	−0.35∗∗∗
			Hallucinations	SPEQ	603	971	−0.27∗∗∗
			Paranoia	GPTS	603	971	−0.27∗∗∗
Garland et al. (2014) [140]	CBTi	Mindfulness	Mood/affect	POMS	40	32	−0.19
			Stress	C–SOSI	40	32	−0.26
Garland et al. (2019) [141]	CBTi	Acupuncture	Anxiety	HADS-A	73	75	0.02
			Depression	HADS-D	73	75	−0.09
Germain et al. (2012) [142]	CBTi + IRT	Prazosin placebo	Anxiety	BAI	12	12	−0.28
			Depression	BDI	12	12	−0.36
			PTSD	PCL	12	12	−0.46
Glozier et al. (2019) [143]	CBTi	Sleep education	Depression	CES-D	31	28	−0.03
Ham et al. (2020) [144]	CBTi	Sleep hygiene	Depression	CES-D	24	20	−0.56
Ho et al. (2014)^a^ [145]	CBTi + telephone support	WLC	Anxiety	HADS-A	49	33	−0.21
			Depression	HADS-D	49	33	−0.13
Ho et al. (2014)^b^ [145]	CBTi	WLC	Anxiety	HADS-A	45	33	−0.19
			Depression	HADS-D	45	33	−0.16
Irwin et al. (2014)^a^ [146]	CBTi	WLC	Depression	IDS-C	46	11	−0.63
Irwin et al. (2014)^b^ [146]	Tai Chi	WLC	Depression	IDS-C	39	12	−0.22
Jansson-Frojmark et al. (2012) [147]	CBTi	WLC	Anxiety	HADS-A	15	15	−1.19∗∗
			Depression	HADS-D	15	15	−1.12∗∗
Jernelov et al. (2012)^a^ [148]	CBTi + telephone support	WLC	Mood/affect	CORE-OM	44	22	−0.50
			Stress	PSS	44	22	−0.64∗
Jernelov et al. (2012)^b^ [148]	CBTi	WLC	Mood/affect	CORE-OM	45	22	−0.39
			Stress	PSS	45	22	−0.30
Jungquist et al. (2012) [149]	CBTi	Self-monitoring	Depression	BDI	14	4	−2.44∗∗∗
Kaldo, V et al. (2015) [150]	CBTi	Mindfulness + sleep hygiene + relaxation	Stress	PSS	54	53	0.00
Kalmbach et al. (2019)^a^ [151]	CBTi	Sleep hygiene	Depression	BDI-II	42	20	−0.45
			Rumination	ERRI	42	20	−0.17
			Rumination	PSWQ	42	20	−0.38
Kalmbach et al. (2019)^b^ [151]	CBTi	Sleep hygiene	Depression	BDI-II	34	20	−0.51
				ERRI	34	20	−0.08
				PSWQ	34	20	−0.53
Katofsky et al. (2012) [152]	CBTi + sleep medication	Sleep medication	Depression	BDI	41	39	−0.11
Kyle et al. (2020) [153]	CBTi	WLC	Depression	PHQ-9	136	166	−0.53∗∗∗
			Anxiety	GAD-7	136	166	−0.33∗∗
Lancee et al. (2012)^a^ [154]	CBTi (digital)	WLC	Anxiety	HADS-A	109	92	−0.17
			Depression	CES-D	109	42	−0.23
Lancee et al. (2012)^b^ [154]	CBTi (booklet)	WLC	Anxiety	HADS-A	126	91	−0.02
			Depression	CES-D	126	41	−0.03
Lancee et al. (2013) [155]	CBTi	CBTi (self-help)	Anxiety	HADS-A	102	95	−0.16
			Depression	CES-D	102	95	−0.32∗
Lee et al. (2020) [156]	Acupuncture	WLC	Depression	HADS-D	49	49	−2.66∗∗∗
			Anxiety	HADS-A	49	49	−0.91∗∗∗
Lichstein et al. (2013) [157]	CBTi	Hypnotic taper	Anxiety	STAI	22	18	−0.35
			Depression	GDS	22	18	−0.72∗
Martinez et al. (2014) [158]	CBTi	Sleep hygiene	Anxiety	SCL-90-R	27	20	−0.06
			Depression	SCL-90-R	27	20	−0.37
McCrae et al. (2019) [159]	CBTi	WLC	Anxiety	STAI	24	23	−0.42
			Depression	BDI	24	23	−0.57
McCurry et al. (1998) [160]	CBTi	WLC	Depression	CES-D	20	9	−0.08
Nguyen et al. (2017) [161]	CBTi	TaU	Anxiety	HADS-A	13	11	−0.98∗
			Depression	HADS-D	13	11	−1.73∗∗∗
Nguyen et al. (2019) [162]	CBTi	TaU	Anxiety	HADS-A	9	6	−0.37
			Depression	HADS-D	9	6	−1.51∗
Norell-Clarke et al. (2015) [163]	CBTi	Relaxation + sleep hygiene	Depression	BDI	24	20	−0.33
Park et al. (2015) [164]	Nordic walking	General walking	Depression	BDI	12	12	−1.10∗
Peoples et al. (2019) [165]	CBTi	Sleep hygiene + Armodafinil + placebo	Depression	PHQ-9	30	30	−0.97∗∗∗
Raskind et al. (2013) [166]	Prazosin	Placebo	Depression	HAM-D	32	35	−0.67∗∗
			Depression	PHQ-9	32	35	−0.69∗∗
			PTSD	CAPS	32	35	−0.83∗∗
Sadler et al. (2018) [167]	CBTi	Sleep education	Anxiety	GAI	22	21	−2.02∗∗∗
			Depression	GDS	22	21	−4.14∗∗∗
Sato et al. (2019) [168]	CBTi	TaU	Anxiety	HADS-A	11	11	−0.81
			Depression	CES-D	11	11	−1.52∗∗
Savard et al. (2005) [169]	CBTi	WLC	Anxiety	HADS-A	27	30	0.35
			Depression	HADS-D	27	30	0.27
Schiller et al. (2018) [170]	CBTi	WLC	Burnout	SMBQ	25	26	−0.03
Sheaves et al. (2017) [171]	CBTi	TaU	Suicidal ideation	BSS	20	20	−0.14
			Psychosis	PANSS pos	20	20	−0.31
			Psychosis	PANSS neg	20	20	−0.51
			Psychosis	PANSS tot	20	20	−0.34
Sheaves et al. (2019) [172]	CBT for nightmares	TaU	Anxiety	DASS-A	11	9	−0.65
			Depression	DASS-D	11	9	0.15
			Dissociation	DES-B	11	9	−0.73
			Hallucinations	CAPS	11	9	−0.10
			Paranoia	GPTS	11	9	−0.82
			Psychosis	DES-B	11	9	−0.73
			Stress	DASS-S	11	9	−0.46
			Suicidal ideation	BSS	11	9	0.48
Song et al. (2020) [173]	CBTi	Sleep hygiene	Depression	BDI	12	13	−0.07
			Anxiety	BAI	12	13	−0.98∗
Tek et al. (2014) [174]	Eszopiclone	Placebo	Depression	CDS	19	17	−0.07
			Psychosis	PANSS-pos	19	17	−0.32
			Psychosis	PANSS-neg	19	17	−0.05
			Psychosis	PANSS-tot	19	17	−0.10
Thiart et al. (2015) [175]	CBTi	WLC	Rumination	PSWQ	59	54	−0.84∗∗∗
Wagley (2010) [176]	CBTi	WLC	Depression	PHQ-9	24	10	−1.55∗∗∗
Wen et al. (2018) [177]	Augmented acupuncture	Standard acupuncture	Depression	HADS-D	43	46	−1.01∗∗∗
Yeung et al. (2011)^a^ [178]	Electroacupuncture	Placebo acupuncture	Depression	HDRS	22	11	−0.28
Yeung et al. (2011)^b^ [178]	Standard acupuncture	Placebo acupuncture	Depression	HDRS	23	12	−0.47
Zhang et al. (2020) [179]	Acupuncture	Sham acupuncture	Depression	SDS	46	44	−3.56∗∗∗
			Anxiety	SAS	46	44	−3.93∗∗∗
Zhu et al. (2018) [180]	Tai Chi	TaU	Depression	SDS	37	12	−0.30

*Note*: ∗*p* < 0.05, ∗∗*p* < 0.01, ∗∗∗*p* < 0.001. CBTi = cognitive behavioural therapy for insomnia, dx = diagnosis, IRT = image rehearsal therapy, MH = mental health, *n*_e_ = number of participants in intervention group, *n*_c_ = number of participants in the control group, PTSD = post-traumatic stress disorder, TaU = treatment as usual, WLC = wait list control. ^ab^Subscript indicates that the study reports multiple eligible interventions in the same study, in these situations both interventions were included as separate studies in the analysis and the control was halved accordingly.

#### Risk of bias

Risk of bias was assessed using the risk of bias assessment criteria developed by the Cochrane Collaboration [60]. RCTs were classified as being at overall risk of bias according to three of the six domains – 1) allocation concealment, 2) blinding of outcome assessment and 3) completeness of outcome data (attrition). RCTs judged as being at low risk of bias for all three domains were judged at overall low risk of bias. RCTs judged as being at high risk for any of the three domains were judged as overall high risk of bias. RCTs judged as a mix of low and unclear risk on these three domains, or all unclear were judged as unclear with respect to risk of bias.

#### Estimating effect sizes

Hedges *g* and the associated standard error were estimated using the means and standard deviations reported by each of the primary studies. Where means and standard deviations were not reported, effect sizes were estimated by converting relevant summary statistics into Hedges *g*. Where studies reported multiple outcome measures for the same/similar constructs (e.g., several measures of depression), effect sizes were computed for each outcome and then meta-analysed in their own right to form one overall effect.

#### Meta-analytic approach

All analyses were conducted in R [61], using the ‘*esc*’ [62], ‘*meta’* [63], ‘*metafor’* [64], ‘*dmetar*’ [65], and ‘*robvis*’ [66] packages. The pooled, sample-weighted, average effect size was computed using a random effects model as effect sizes between studies are likely to vary considerably [67]. Following Cohen's recommendations [68], *g* = 0.20 was taken to represent a ‘small’ effect size, *g* = 0.50 a ‘medium’ effect size and *g* = 0.80 a ‘large’ effect size. The *I*^2^ statistic was used to assess heterogeneity of effect sizes across the included studies and was interpreted according to the classifications suggested by Higgins et al. [69], where *I*^2^ = 25% indicates low heterogeneity, *I*^2^ = 50% indicates moderate heterogeneity, and *I*^2^ = 75% indicates high heterogeneity. Publication bias was assessed via visual inspection of a funnel plot and Egger's test [70]. Additionally, Orwin's formula [71] was used to determine the fail-safe *n*. Finally, outliers were defined as any effect size for which the confidence intervals did not overlap with the confidence interval of the pooled effect [72]. We conducted a sensitivity analysis examining the effect of outliers for each outcome by rerunning the analysis with any outlying effect sizes removed.

#### Subgroup analyses

Moderation analysis was conducted to identify variables that were associated with the effect of improving sleep on mental health outcomes. A minimum of three studies representing each moderator level category was required in order to conduct moderation analysis. For categorical variables, the analysis was based on a mixed effects model, in that the pooling of effect sizes within each moderator level was based on a random effects model, while the comparison of effect sizes between levels was based on a fixed effects model. The *Q* statistic was then used to assess whether effect sizes were significantly different between moderator levels. For continuous variables, sample-weighted meta-regression was used to investigate the impact of the moderator on mental health effect sizes.

#### Data availability statement

All data and analysis code are freely available on the Open Science Framework under a creative commons 4.0 license (for access, see [73]).

## Results

### Study selection

**Fig. 1:**
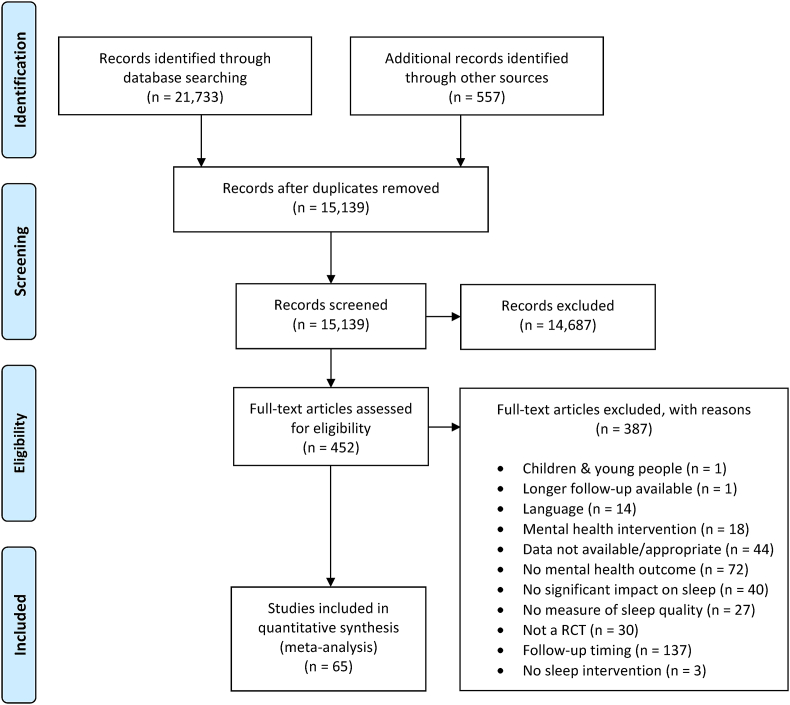
PRISMA diagram showing the flow of studies through the review.

Fig. 1 shows the flow of records through the review. Systemic searches of the published and grey literature retrieved a total of 21,733 records, which was reduced to 15,139 after duplicates were removed. Of these records, 14,687 (97%) were excluded in the first stage of screening, leaving 452 full-text records to be screened. Of these records, 387 (86%) were cross-referenced against the review eligibility criteria and excluded (see Fig. 1 for a breakdown of reasons and Supplementary Materials 2 for a list of the studies excluded at this stage), leaving 65 records for inclusion in the meta-analysis.

### Study characteristics

Table 2 describes key characteristics of the included studies. The 65 studies provided 72 comparisons between an intervention that successfully improved sleep quality vs. a control group.

#### Participants

A total of *N* = 8608 participants took part across the 72 interventions. 38 of the comparisons (53%) included participants with a comorbid physical or mental health problem, while 31 (43%) reported no comorbid health problems, and 3 (4%) reported insufficient detail to make a judgement. Of the 38 comparisons including participants with comorbid health problems, 18 (47%) reported mental health diagnoses, and 20 (53%) had physical health problems.

#### Outcome measures

The majority of comparisons (61, 85%) reported a measure of depression, but 33 (46%) reported a measure of anxiety, 6 (8%) reported a measure of stress, 5 (7%) reported measures of psychosis spectrum experiences (e.g., total, positive, and negative symptoms), 9 (13%) reported a measure of general mood, 2 (3%) reported post-traumatic stress disorder outcomes, 2 (3%) reported measures of suicidal ideation, 4 (6%) reported rumination outcomes, and 1 (2%) reported a measure of psychological burnout.

#### Interventions and comparisons

Most interventions were multi-component CBTi (53, 74%), but interventions also involved acupuncture (7, 10%), pharmacological treatments (2, 3%), sleep hygiene alone (2, 3%), sleep restriction alone (2, 3%), Tai Chi (2, 3%), CBT for nightmares (1, 2%), herbal remedies (1, 2%), walking (1, 2%), and yoga (1, 2%). Interventions were most often compared against an active control group (34, 47%), but were also compared to waitlist control groups (25, 35%), and groups receiving treatment as usual (13, 18%). On average participants’ mental health was followed-up 20.5 weeks post-intervention (median = 12 weeks post-intervention), with the earliest follow-up being 4-weeks post-intervention, and the furthest follow-up 156-weeks (three years) post intervention.

### Manipulation check: did sleep quality improve significantly in the intervention group relative to controls?

Before we examined the effect of improving sleep quality on subsequent mental health, we confirmed that studies included in the review successfully improved sleep quality. The interventions had large and statistically significant effects on sleep quality at the earliest follow-up point reported (*g*_+_ = −1.07, 95% CI = −1.26 to −0.88, *p* < 0.001), although heterogeneity between studies was substantial (*I*^*2*^ = 79%, *Q* = 331.93, *p* < 0.001). After twelve outlying effect sizes were removed, the effect of the interventions on sleep quality remained large and statistically significant (*g*_+_ = −0.97, 95% CI = −1.07 to −0.88, *p* < 0.001), and heterogeneity was reduced to moderate levels (*I*^*2*^ = 43%, *Q* = 102.32, *p* < 0.001). These findings suggest that the primary studies included in the present review successfully manipulated sleep quality, even after accounting for outliers.

### What effect do improvements in sleep quality have on mental health?

Table 3 presents the effect of improving sleep quality on composite mental health outcomes, and on measures of depression, anxiety, stress, psychosis spectrum experiences, suicidal ideation, PTSD, rumination, and burnout.

**Table 3:** The effect of improving sleep on mental health outcomes.

Outcome	*g*_*+*_	95% CI	*I*^*2*^	*Q*	*k*	*N*
Composite outcomes	−0.53∗∗∗	−0.69 to −0.38	76%	291.94∗∗∗	72	8608
Depression	−0.63∗∗∗	−0.84 to −0.43	81%	322.03∗∗∗	61	7868
Anxiety	−0.51∗∗∗	−0.77 to −0.24	82%	186.92∗∗∗	35	5819
Stress	−0.42∗	−0.79 to −0.05	55%	11.05	6	419
Psychosis spectrum						
PANSS total	−0.17	−0.53 to 0.19	0%	0.41	3	121
Positive symptoms	−0.26∗	−0.43 to −0.08	0%	1.71	5	1715
Negative symptoms	−0.28	−3.22 to 2.65	0%	1.00	2	76
Suicidal ideation	0.10	−3.74 to 3.94	20%	1.25	2	60
PTSD	−0.72	−2.90 to 1.46	0%	0.59	2	91
Rumination	−0.49∗	−0.93 to −0.04	36%	4.65	4	355
Burnout	−0.03	−0.58 to 0.52	–	–	1	51

*Notes*: ∗∗∗*p* < 0.001, ∗*p* < 0.05, PANSS = Positive and Negative Symptoms Scale, PTSD = Post Traumatic Stress Disorder.

#### Composite mental health

**Fig. 2:**
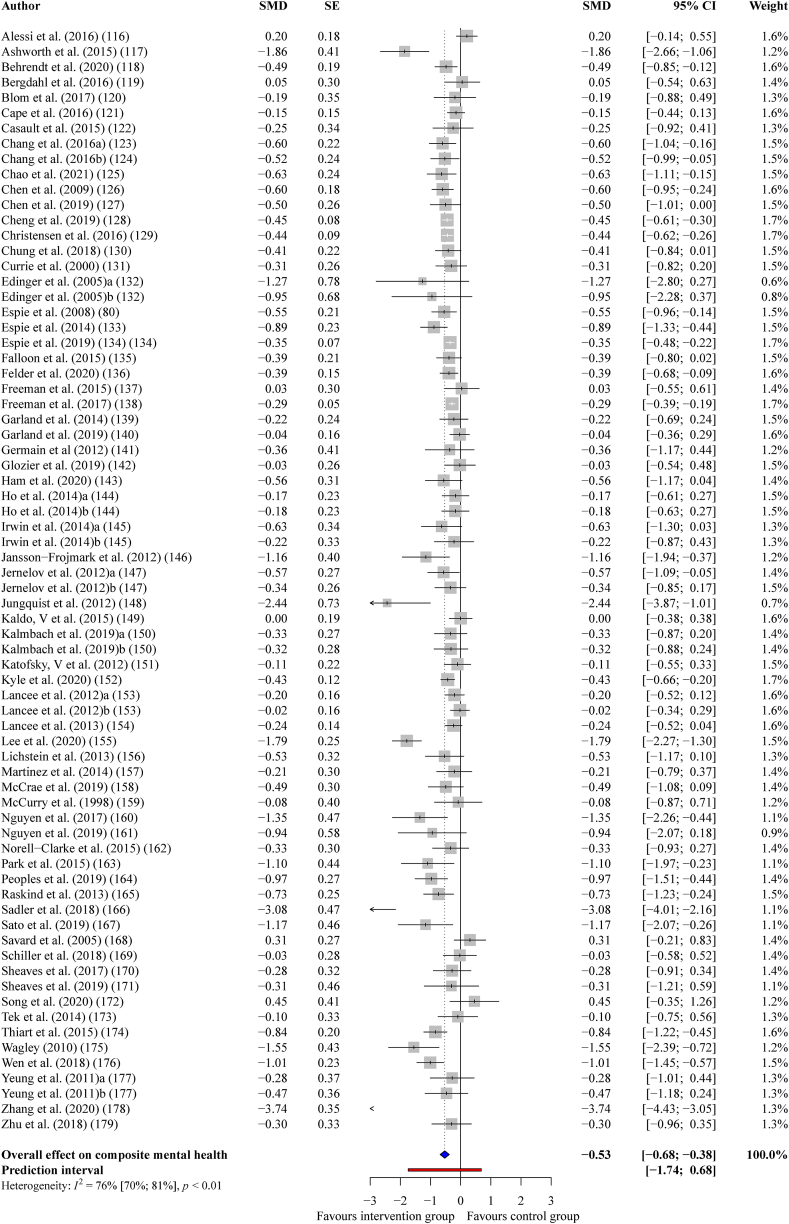
Forest plot showing the effect of improving sleep on composite mental health outcomes.

On average, the 72 interventions that successfully improved sleep quality had a statistically significant, medium-sized effect on subsequent composite mental health outcomes, (*g*_+_ = −0.53, 95% CI = −0.68 to −0.38, *p* < 0.001); however, there was substantial heterogeneity between the effect sizes, (*I*^*2*^ = 76%, *Q* = 291.94, *p* < 0.001). After re-running the analysis with eleven outlying effect sizes removed, the effect of improving sleep on composite mental health outcomes was small-to-medium sized but still statistically significant, (*g*_+_ = −0.42, 95% CI = −0.49 to −0.34, *p* < 0.001) and now relatively homogeneous (*I*^*2*^ = 20%, *Q* = 75.24, *p* = 0.0888). See Fig. 2 for a forest plot.

#### Depression

**Fig. 3:**
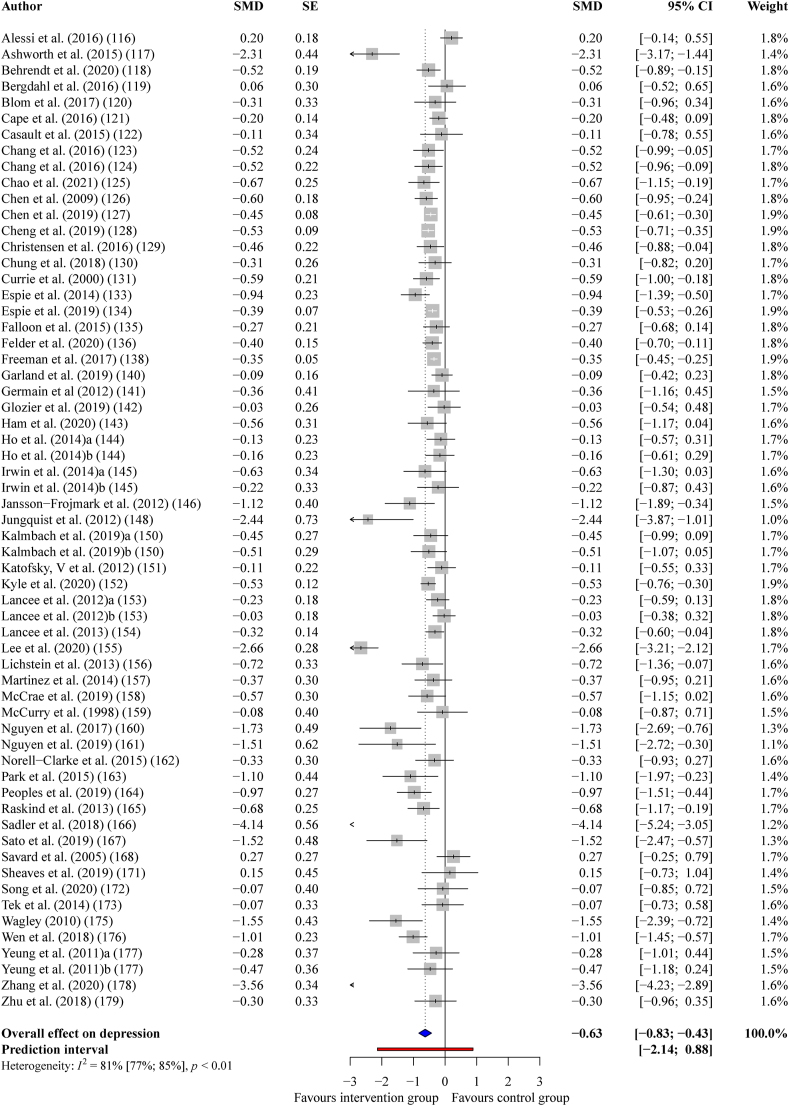
Forest plot showing the effect of improving sleep on depression.

Interventions that successfully improved sleep quality had a statistically significant, medium-sized effect on depression across 61 comparisons, (*g*_+_ = −0.63, 95% CI = −0.83 to −0.43, *p* < 0.001); however, once again, there was substantial heterogeneity, (*I*^*2*^ = 81%, *Q* = 322.09, *p* < 0.001). After re-running the analysis with nine outlying effect sizes removed, the effect of improving sleep on depression remained medium-sized, (*g*_+_ = −0.47, 95% CI = −0.57 to −0.37, *p* < 0.001), with moderate heterogeneity, (*I*^*2*^ = 32%, *Q* = 74.86, *p* = 0.0164). See Fig. 3 for a forest plot.

#### Anxiety

**Fig. 4:**
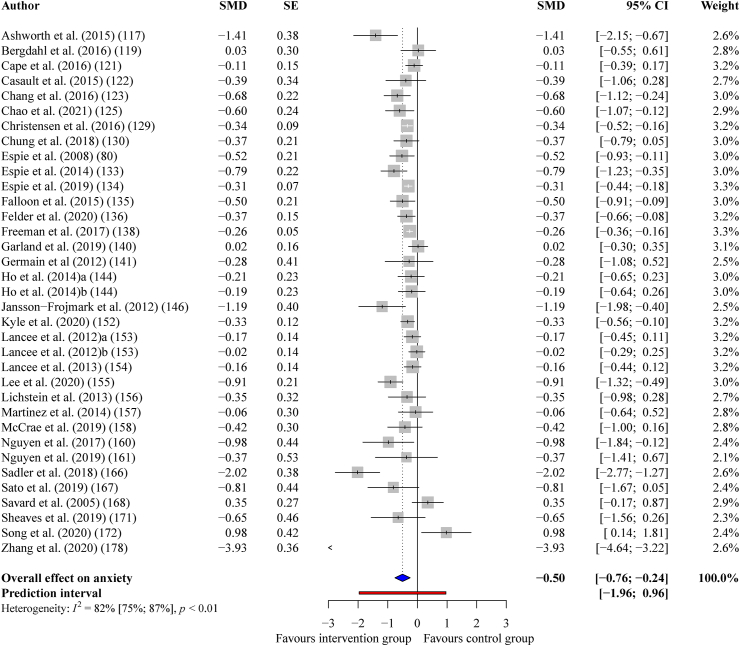
Forest plot showing the effect of improving sleep on anxiety.

Interventions that successfully improved sleep quality had a statistically significant, small-to-medium sized effect on anxiety across 35 comparisons, (*g*_+_ = −0.50, 95% CI = −0.76 to −0.24, *p* < 0.001), with substantial levels of heterogeneity, (*I*^*2*^ = 82%, *Q* = 187.02, *p* < 0.001). After re-running the analysis with four outlying effect sizes removed, the effect improving sleep on anxiety outcomes was small-to-medium sized, but still statistically significant, (*g*_+_ = −0.38, 95% CI = −0.49 to −0.27, *p* < 0.001), with lower levels of heterogeneity, (*I*^*2*^ = 43%, *Q* = 52.49, *p* = 0.0067). See Fig. 4 for a forest plot.

#### Stress

**Fig. 5:**
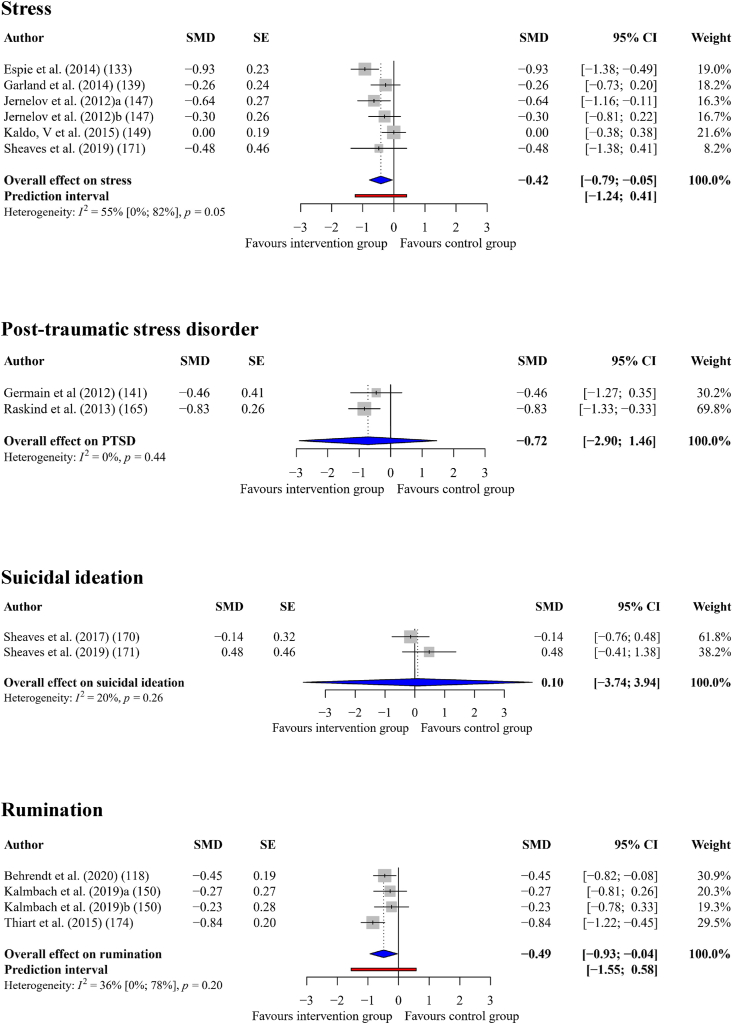
Forest Plot Showing the Effect of Improving Sleep on Stress, Suicidal Ideation, PTSD, and rumination.

Interventions that successfully improved sleep quality had a statistically significant, small-to-medium sized effect on stress (*g*_+_ = −0.42, 95% CI = −0.79 to −0.05, *p* = 0.033), across six comparisons. There were moderate levels of heterogeneity (*I*^*2*^ = 55%, *Q* = 11.05, *p* = 0.05), but there were no outlying effect sizes. See Fig. 5 for a forest plot.

#### Psychosis spectrum experiences

**Fig. 6:**
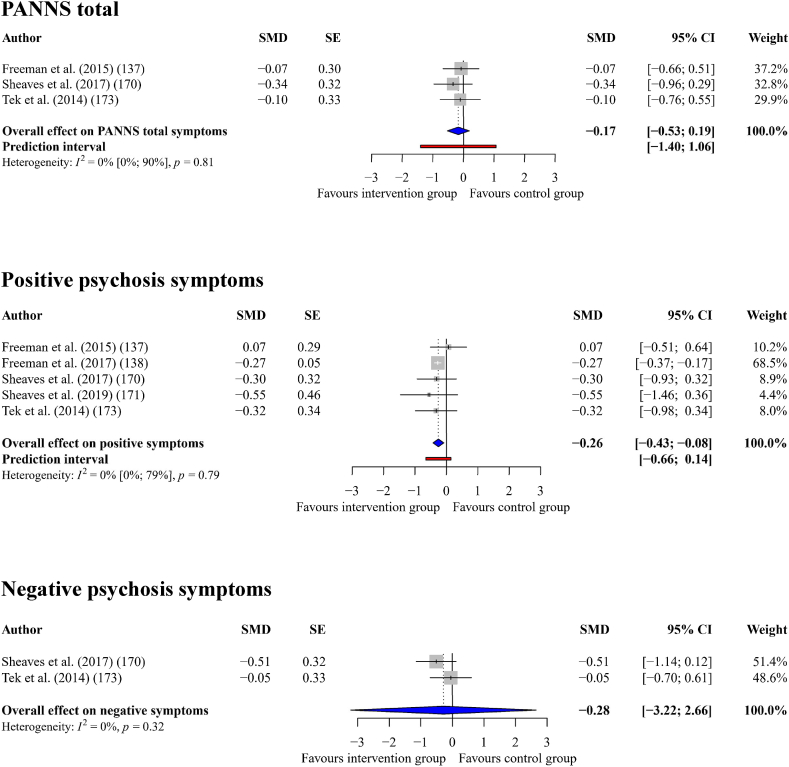
Forest plot showing the effect of improving sleep on psychosis spectrum outcomes.

Interventions that successfully improved sleep quality had a small effect on total symptoms as indicated by the PANSS (*g*_+_ = −0.17, 95% CI = −0.53 to 0.19, *p* = 0.18) across three comparisons, with zero heterogeneity (*I*^*2*^ = 0%, *Q* = 0.41, *p* = 0.813). Interventions that successfully improved sleep quality had a small effect on positive symptoms (*g*_+_ = −0.26, 95% CI = −0.43 to −0.08, *p* = 0.014) across five comparisons, with zero heterogeneity (*I*^*2*^ = 0%, *Q* = 1.71, *p* = 0.788). Finally, interventions that successfully improved sleep quality had a small effect on negative symptoms (*g*_+_ = −0.28, 95% CI = −3.22 to 2.65, *p* = 0.436) across *k* = 2 comparisons, with zero heterogeneity (*I*^*2*^ = 0%, *Q* = 1, *p* = 0.318). See Fig. 6 for a forest plot.

#### Suicidal ideation

Interventions that successfully improved sleep quality had a small, adverse effect on suicidal ideation (*g*_+_ = 0.10, 95% CI = −3.74 to 3.94, *p* = 0.804) across two comparisons. There were low levels of heterogeneity (*I*^*2*^ = 20%, *Q* = 1.25, *p* = 0.263) and no outlying effect sizes. See Fig. 5 for a forest plot.

#### Post-traumatic stress disorder (PTSD)

Interventions that successfully improved sleep quality had a medium-to-large effect on PTSD (*g*_+_ = −0.72, 95% CI = −2.90 to 1.46, *p* = 0.149) across two comparisons, with zero heterogeneity (*I*^*2*^ = 0%, *Q* = 0.59, *p* = 0.442). See Fig. 5 for a forest plot.

#### Rumination

Interventions that successfully improved sleep quality had a statistically significant, medium sized effect on rumination (*g*_+_ = −0.49, 95% CI = −0.93 to −0.04, *p* = 0.041) across four comparisons, with moderate heterogeneity (*I*^*2*^ = 36%, *Q* = 4.65, *p* = 0.1991). See Fig. 5 for a forest plot.

#### Burnout

Only one study reported the effect of improving sleep on burnout finding almost zero effect (*g* = −0.03, CI = −0.58 to 0.52, *p* = 0.917).

### Moderators of the effect of improving sleep quality on composite mental health outcomes

Table 4 presents the findings of analyses evaluating categorical moderators of the effect of improving sleep quality on composite mental health outcomes and Table 5 presents analyses evaluating continuous moderators using meta-regression. Studies that found significant effects of the intervention on sleep quality reported larger effects on subsequent composite mental health, (*g* = −0.53, 95% CI = −0.68 to −0.38, *p* < 0.001), than studies that did not find a significant effect of the intervention on sleep quality, (*g* = −0.12, 95% CI = −0.24 to 0.01, *p* = 0.0522), a difference that was statistically significant, (*Q* = 17.59, *p* < 0.001). This finding strengthens the notion that improvements in sleep are behind improvements in mental health. The effect of improving sleep on mental health was larger in studies with shorter follow-up periods, (i.e., <6 months, *g*+ = −0.60), than in studies with longer follow-ups, (i.e., 6 months, *g*+ = −0.18, *Q* = 10.75, *p* < 0.01). Furthermore, interventions that were delivered face-to-face by a clinician or therapist were associated with significantly larger effects on mental health, (*g*+ = −0.63), than those that were self-administered by participants, (*g*+ = −0.34, *Q* = 4.50, *p* < 0.05). Finally, there was significant variation in the size of the effect between countries (*Q* = 53.69, *p* < 0.001). No other statistically significant categorical moderator effects were found. Regarding continuous moderators, meta-regression revealed a statistically significant dose–response effect for the association between the effect of interventions on sleep quality and the effect on subsequent mental health outcomes (*B* = 0.77, 95% CI = 0.52 to 1.02, *p* < 0.001), suggesting that greater improvements in sleep led to greater improvements in mental health. No other continuous variables significantly moderated the effect of improving sleep on mental health.

**Table 4:** Categorical moderators of the effect of improving sleep on composite mental health outcomes.

Variable	Levels	*k*	*g*_+_	95% CI	*Q*
Significant effect on sleep^a^	Yes	72	−0.53	−0.69 to −0.38	17.69∗∗∗
	No	31	−0.12	−0.24 to 0.01	
Clinical status of MH	Clinical	15	−0.72	−1.14 to −0.30	0.92
	Non-clinical	45	−0.50	−0.68 to −0.31	
Comorbidities	Mental health	18	−0.64	−1.00 to −0.29	0.63
	Physical health	20	−0.54	−0.76 to −0.32	
	No comorbidities	31	−0.47	−0.72 to −0.23	
Follow-up point	Short (<6 months)	61	−0.60	−0.77 to −0.42	10.75∗∗
	Long (≥6 months)	11	−0.18	−0.36 to −0.00	
Assessment type	Self-reported	66	−0.54	−0.70 to −0.38	0.62
	Clinician rated	6	−0.44	−0.65 to −0.23	
Adjusted data	Adjusted	21	−0.51	−0.77 to −0.26	0.01
	Unadjusted	51	−0.53	−0.72 to −0.35	
Recruitment setting	Clinical (MH)	12	−0.52	−1.00 to −0.04	3.72
	Clinical (PH)	14	−0.52	−0.76 to −0.28	
	Community	39	−0.39	−0.53 to −0.26	
	Mixed	9	−1.12	−1.94 to −0.31	
Recruitment method	Voluntary	49	−0.46	−0.58 to −0.34	0.98
	Health professional	7	−0.65	−1.45 to 0.14	
	Mixed	8	−0.88	−1.80 to 0.04	
Control group	Active control	34	−0.58	−0.87 to −0.30	0.57
	TaU	13	−0.52	−0.75 to −0.29	
	Wait-list	25	−0.46	−0.63 to −0.29	
Risk of bias	High	31	−0.38	−0.56 to −0.21	0.74
	Low	10	−0.55	−0.91 to −0.20	
Intervention type	Acupuncture	7	−1.17	−2.08 to −0.25	2.46
	CBTi	53	−0.44	−0.59 to −0.29	
	Exercised based^b^	4	−0.52	−0.85 to −0.19	
	Pharmacological^c^	2	–	–	
	Sleep hygiene only^c^	2	–	–	
	Sleep restriction only^c^	1	–	–	
	CBT for nightmares^c^	1	–	–	
	Herbal tea^c^	1	–	–	
Intervention format	Group	11	−0.42	−0.92 to 0.08	0.25
	Individual	52	−0.55	−0.73 to −0.38	
Intervention delivery	Clinician delivered	43	−0.63	−0.87 to −0.38	4.50∗
	Self-administered	23	−0.34	−0.43 to −0.26	
Country of origin	Australia	5	−1.50	−2.39 to −0.60	53.69∗∗∗
	Canada	4	−0.12	−0.40 to 0.17	
	China	8	−0.85	−1.59 to −0.11	
	Germany	3	−0.49	−0.90 to −0.08	
	Korea	4	−0.78	−1.70 to 0.15	
	Netherlands	3	−0.16	−0.29 to −0.03	
	Sweden	8	−0.28	−0.53 to −0.03	
	Taiwan	4	−0.57	−0.61 to −0.52	
	UK	9	−0.36	−0.51 to −0.22	
	USA	20	−0.50	−0.71 to −0.28	
	New Zealand^c^	2	–	–	
	Spain^c^	1	–	–	

*Notes*: CBTi = cognitive behavioural therapy for insomnia, MH = Mental Health, PH = Physical Health, TaU = treatment as usual, WLC = wait list control.

∗*p* < 0.05, ∗∗*p* < 0.01, ∗∗∗*p* < 0.001.

a Studies in the ‘No’ moderator category were excluded from the review due to having no significant effect on sleep quality but were included for moderation analysis.

b The ‘exercise based’ category combines separate interventions with exercise as a key element, including walking, yoga and Tai Chi.

c Not included in subgroup analysis due to low number.

**Table 5:** Continuous moderators of the effect of improving sleep on composite mental health outcomes.

Variable	*k*	B	SE	95% CI
Publication year	72	−0.02	0.02	−0.05 to 0.02
Journal impact	71	0.01	0.01	−0.01 to 0.03
Age	71	0.00	0.01	−0.01 to 0.02
Sex	71	0.00	0.00	−0.00 to 0.01
Sleep effect	70	0.77∗∗∗	0.13	0.52 to 1.02
Intervention duration	70	0.02	0.02	−0.03 to 0.06
Contact time	55	0.00	0.01	−0.02 to 0.01
Number of sessions	61	0.00	0.01	−0.02 to 0.01

#### Post-hoc moderation analysis

##### Is the smaller effect of improving sleep on mental health at longer follow-ups associated with smaller effects on sleep quality?

We conducted further (unplanned) post-hoc analysis to investigate whether the smaller effect of improving sleep on mental health at longer follow-ups was accompanied by a reduction in the improvements to sleep quality. Studies reporting the effect of the intervention at shorter follow-ups reported larger improvements in sleep quality, (*g* = −1.03, 95% CI = −1.27 to −0.78, *p* < 0.001), than those reporting longer follow-ups (*g* = −0.44, 95% CI = −0.62 to −0.27, *p* < 0.001), a difference that was statistically significant, (*Q* = 14.38, *p* < 0.001). This suggests that the smaller effect of improving sleep on mental health at longer follow-ups might be driven by a smaller effect of the interventions on sleep quality at longer follow-ups.

##### Can some of the effect of improved mental health be explained by CBTi modules that target processes associated with mental health?

Finally, although the present review excluded interventions that specifically and directly targeted mental health, some CBTi protocols include modules that might target similar processes associated with some mental health difficulties (rumination around sleep, catastrophizing over the effect of poor sleep etc.). Therefore, we compared CBTi interventions with modules that could target processes associated with mental health vs. interventions that did not include these modules (e.g., sleep restriction alone, sleep hygiene alone, herbal tea, and pharmacological intervention). There were no significant differences in the effect of improved sleep quality on mental health between CBTi interventions including modules addressing processes associated with mental health (*g* = −0.44, 95% CI = −0.59 to −0.29, *p* < 0.001), relative to those that did not (*g* = −0.48, 95% CI = −0.65 to −0.32, *p* < 0.001, *Q* = 2.51, *p* = 0.285). This finding suggests that it is the beneficial effect of improved sleep quality that confers improvements in mental health rather than the inclusion of modules that target processes associated with mental health commonly seen in CBTi protocols.

### Risk of bias assessments

**Fig. 7:**
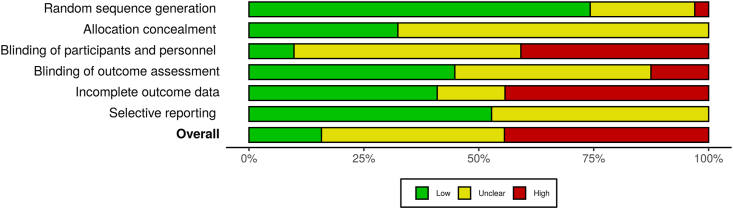
Weighted risk of bias summary plot.

Fig. 7 summarizes the weighted assessment of risk of bias. Individual risk of bias judgements for included studies are presented in Supplementary Material 3. Ten studies (15%) were judged as having low risk of bias, 29 studies (45%) were judged as high risk of bias, and 26 studies (40%) were judged as unclear. The methodological quality of the included studies was not associated with the effect of improving sleep on composite mental health outcomes, *Q* = 0.72, *p* = 0.395.

### Publication bias

**Fig. 8:**
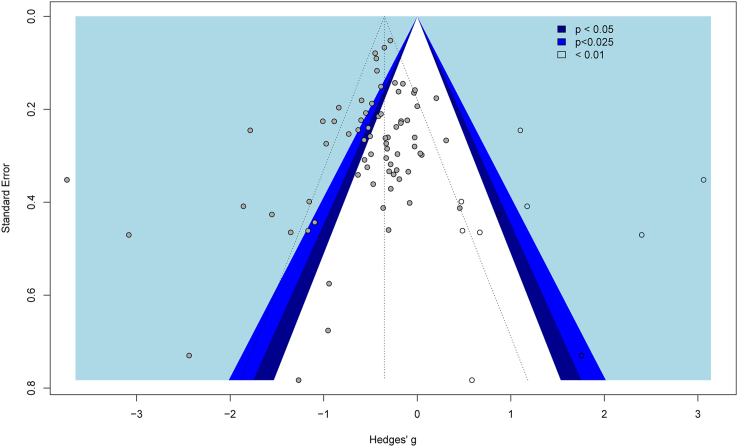
Contrast enhanced funnel plot for the effect of improving sleep on composite mental health (solid grey markers) with imputed studies (hollow markers).

A funnel plot of the effect of improving sleep quality on composite outcomes revealed asymmetry in the effect sizes (Egger's regression = −1.09, 95% CI = −1.91 to −0.28, *p* < 0.05, see Fig. 8). Duval and Tweedie's [74] trim and fill procedure was therefore used to address the asymmetry. Ten studies were imputed resulting in a statistically significant, small-to-medium sized adjusted effect of improving sleep on composite mental health outcomes (*g*_+_ = −0.35, 95% CI = −0.55 to −0.16, *p* < 0.001). Orwin's failsafe *n* test suggested that an additional 4101 comparisons producing null effects would be needed to reduce the average effect of improving sleep on composite outcomes to zero. Taken together these results suggest that the effect of improving sleep on composite mental health is robust to possible publication bias.

## Discussion

The present review used meta-analysis to synthesize the effect of 72 interventions that improved sleep quality relative to a control condition on subsequent mental health. The findings revealed that improving sleep quality had, on average, a medium-sized effect on mental health, including clear evidence that improving sleep reduced depression, anxiety, and stress. A dearth of primary studies of other mental health difficulties (e.g., psychosis spectrum experiences, suicidal ideation, PTSD, rumination, and burnout) mean that it is premature to draw definitive conclusions in these areas. It was also notable that we found a dose–response relationship between improvements in sleep quality and subsequent mental health, such that greater improvements in sleep led to greater improvements in mental health. Although there was some evidence of publication bias, the effects remained robust to correction. Taken together, the findings suggest that improving sleep leads to better mental health, therefore providing strong evidence that sleep plays a causal role in the experience of mental health difficulties.

### Sleep as a transdiagnostic treatment target

The present findings support the idea that targeting sleep promotes mental health across a range of populations and experiences. The effect of improving sleep quality on composite mental health was medium-sized and statistically significant, regardless of the presence of physical and/or mental health comorbidities. This finding is particularly important given the healthcare challenges associated with multimorbidity [75] and mental and physical health problems often co-occur [[76], [77], [78], [79]], something that appears to be increasing [80]. Consequently, it is important that the benefits of improving sleep on mental health occur even in the presence of comorbid health complaints, as was reported in the present research. Improving sleep has also been shown to improve aspects of physical health, including fatigue [81], chronic pain [82,83], and overall health related quality of life [84] and could reduce the cost of healthcare. For example, offering a digital CBTi intervention (Sleepio) to primary care patients was associated with an average saving of £70.44 per intervention user [85], and cost savings following sleep intervention have also been specifically reported in people with comorbid mental health difficulties such as depression [86].

Another finding to suggest that targeting sleep could promote mental health across a range of populations and experiences, is that we found no difference in the effect of improving sleep quality on mental health between those with clinically defined mental health difficulties and those with non-clinical experiences or between those recruited from clinical vs. community settings, with both groups receiving significant benefits of improved sleep on mental health. This suggests that improving sleep could prove helpful across a range of mental health severities, thus broadening the possible impact of sleep interventions within healthcare services. Finally, there is growing evidence that sleep disturbances predict the development of mental health difficulties in the future. For example, shorter and more variable sleep has been shown to be longitudinally associated with more severe hallucinations and delusional ideation in those at high-risk of psychosis [87]. The present research found that improving sleep has a significant beneficial impact on future mental health in those with non-clinical experiences, raising the possibility that delivering interventions that improve sleep early might limit the risk of developing (or exacerbating) substantive mental health difficulties. Indeed, less severe mild-to-moderate presentations of mental health difficulties can develop over time into more severe mental health diagnoses [88,89], therefore improving sleep might be one tool that can be used in combination with others to limit the risk of transition.

### Strengths and limitations

The present review has several strengths. First, it provides a comprehensive and up-to-date search of RCTs examining the effect of improving sleep on a variety of subsequent mental health outcomes. Indeed, with 65 RCTs and *N* = 8608 participants, the present review is one of the largest studies of the effect of improving sleep on mental health to date. Second, the review was specifically designed to test the causal association between sleep and mental health (i.e., RCTs only, successful sleep improvement required, temporal lag between measures etc.). To our knowledge, the review is the first to adopt this approach in the field of sleep and mental health, although the general approach has been used in other fields [90]. Finally, we provide an analysis of possible moderators of the effect of improving sleep on mental health, identifying several key moderators of the effect.

However, there are limitations that must be considered when interpreting the findings. First, relatively few studies examined the effect of improving sleep over the long term. Those that did report longer follow-ups generally found smaller effects (although still statistically significant), most likely due to the diminishing effects of interventions on sleep quality over time [91]. Consequently, it is important that interventions targeting sleep quality as a route to improving mental health seek to maintain their beneficial effects. Second, there were few primary studies for some of the outcomes included in this review. Consequently, in lieu of more studies reporting these outcomes, the inferences that we can make for mental health outcomes other than depression and anxiety are more limited. Third, although the intention of the present review was to include a broad range of sleep disturbances, most of the analyses are based on CBT interventions for insomnia. This might be due to the relationship between insomnia and mental health being the one that is historically most studied. However, it may be that our focus on sleep quality precluded some studies that do not focus on insomnia from inclusion. For example, different sleep disorders have different conceptualisations of improvement that might not include sleep quality. For example, the timing of sleep is particularly important in circadian rhythm disorders and daytime sleepiness is a key outcome in sleep apnoea research. Future research might consider examining the effect of improving specific sleep disorders on mental health by conceptualising improvements using sleep disorder specific outcomes.

### Future directions

The present review highlighted several areas for future research in terms of both research and theory, and the implementation of findings in practice. First, given that mental health was measured on average around 20.5 weeks post-intervention in the primary studies, and that the effect of improving sleep on mental health significantly reduced over time, future research should examine the effect of improving sleep on mental health over the longer term. Second, although not uncommon, the majority of RCTs included in the present review were at high risk, or unclear risk or bias. Consequently, in addition to studying the effect of improving sleep over the longer term, on a range of mental health difficulties beyond depression and anxiety, we need more research at lower risk of methodological bias.

Finally, although the present research provides evidence for a causal association between sleep and mental health, it is less clear *how* sleep affects mental health. One potential mechanism is whether and how people regulate their emotions (e.g., in response to negative events). Indeed, evidence suggests that poor sleep can amplify the adverse effect of negative life events [92,93], dull the beneficial impact of positive events [94], and is associated with more frequent use of emotion regulation strategies that might be detrimental to good mental health [95]. By extension, although we are unaware of RCTs testing the effect of improved sleep on emotion regulation, changes in sleep are prospectively associated with changes in aspects of emotion regulation [96,97], while experimentally induced sleep deprivation is adversely linked to poorer emotion regulation [96,97]. Contemporary perspectives on emotion regulation (e.g., the action control perspective), draw on research on how people regulate their behaviour, to propose that regulating emotions involves three tasks, 1) identifying the need to regulate, 2) deciding whether and how to regulate, and 3) enacting a regulation strategy [98]. We propose that poor sleep quality has the potential to adversely affect anyone (or all) of the three tasks involved in effectively regulating emotions, which might go some way toward explaining the relationship between poor sleep and mental health. Therefore, we would recommend that future research includes measures of aspects of emotion regulation (e.g., the Difficulties in Emotion Regulation Scale, [99]) within experimental and longitudinal designs to elucidate possible mechanisms by which improvements in sleep benefit mental health.

In terms of practice and implementation, evidence on the effect of sleep on mental health also supports calls for routine screening and treatment of problems with sleep. Both the Royal Society for Public Health (RSPH) and the Mental Health Foundation (MHF) recommend that primary health care training should include awareness of, and skills in assessing, sleep problems [100,101]. Despite this and a growing body of evidence, there has been little progress to date [102]. This may reflect under-appreciation of the importance of sleep [103] and lack of training and skills in assessing and managing sleep problems [[104], [105], [106], [107], [108]], as well as limited time and resources [103,109]. Therefore, a profitable next step might be to explore barriers and facilitators to assessing sleep and delivering effective interventions in specific care settings, from both the patient and clinician perspective. Indeed, the present review also highlighted a dearth of trials that tested the effect of improving sleep on mental health outcomes in ‘real world’ settings (e.g., within existing clinical and community health services). Although some researchers are taking important steps in this area [[110], [111], [112]], there is a clear need for more trials of interventions in clinical services so that the effectiveness and implementation of such interventions in routine care can be better understood.

### Conclusions

Taken together, the present research supports the view that sleep is causally related to the experience of mental health difficulties, and therefore that sleep represents a viable treatment target that can confer significant benefits to mental health, as it has been found to do for physical health. We found that improving sleep was associated with better mental health regardless of the severity of mental health difficulty (i.e., clinical vs. non-clinical) or the presence of comorbid health conditions. Poor sleep is almost ubiquitous within mental health services [102,108,113,114], is causally related to the experience of mental health difficulties, and represents a potential treatment target [105,115,116]. Consequently, equipping health professionals with greater knowledge and resources to support sleep is an essential next step. Future research should consider how interventions that improve sleep could be better incorporated into routine mental health care, as well as the possible mechanisms of action that might explain how sleep exerts its effects on mental health.

**Research agenda**

To fully harness the effect of improved sleep on mental health, it is important that future research:

1. Explores the barriers and possible solutions to incorporating interventions that improve sleep into mental health care services.
2. Tests the effect of improving sleep on mental health outcomes beyond depression and anxiety, and over the long term, using designs at low risk of methodological bias.
3. Investigates the possible mechanisms of action that might explain how sleep exerts its effects on the experience of mental health difficulties.

**Practice points**

• Sleep is causally related to the experience of mental health difficulties and represents a viable transdiagnostic treatment target for those experiencing mental health difficulties.
• Improving sleep has beneficial effects on the experience of mental health difficulties, regardless of the severity of those difficulties, or the presence of comorbid health conditions.
• Healthcare professionals aiming to improve mental health (particularly depression, anxiety, and stress) should consider interventions designed to improve sleep, particularly cognitive behavioral therapy for insomnia where the evidence base is strongest.

## Funding

This research was funded by the 10.13039/501100000272National Institute for Health Research under its 10.13039/501100009128Research for Patient Benefit (RfPB) Programme (Grant Reference Number PB-PG- 0817-20027). The views expressed are those of the author(s) and not necessarily those of the NIHR or the Department of Health and Social Care.

## References

[1] Ohayon M.M. (2011). Epidemiological overview of sleep disorders in the general population. Sleep Med Res.

[2] Kerkhof G.A. (2017). Epidemiology of sleep and sleep disorders in The Netherlands. Sleep Med.

[3] Chattu V.K., Manzar M.D., Kumary S., Burman D., Spence D.W., Pandi-Perumal S.R. (2019). Healthcare.

[4] Bebbington P.E., McManus S. (2020). Revisiting the one in four: the prevalence of psychiatric disorder in the population of England 2000–2014. Br J Psychiatry.

[5] Twenge J.M., Cooper A.B., Joiner T.E., Duffy M.E., Binau S.G. (2019). Age, period, and cohort trends in mood disorder indicators and suicide-related outcomes in a nationally representative dataset, 2005–2017. J Abnorm Psychol.

[6] Hale L., Troxel W., Buysse D.J. (2020). Sleep health: an opportunity for public health to address health equity. Annu Rev Publ Health.

[7] Robotham D. (2011). Sleep as a public health concern: insomnia and mental health. J Publ Ment Health.

[8] Ford D.E., Kamerow D.B. (1989). Epidemiologic study of sleep disturbances and psychiatric disorders: an opportunity for prevention?. JAMA.

[9] Baglioni C., Nanovska S., Regen W., Spiegelhalder K., Feige B., Nissen C. (2016). Sleep and mental disorders: a meta-analysis of polysomnographic research. Psychol Bull.

[10] Stepanski E.J., Rybarczyk B. (2006). Emerging research on the treatment and etiology of secondary or comorbid insomnia. Sleep Med Rev.

[11] McCrae C.S., Lichstein K.L. (2001 Feb). Secondary insomnia: diagnostic challenges and intervention opportunities. Sleep Med Rev.

[bib12] Alvaro P.K., Roberts R.M., Harris J.K. (2013). A systematic review assessing bidirectionality between sleep disturbances, anxiety, and depression. Sleep.

[bib13] Baglioni C., Battagliese G., Feige B., Spiegelhalder K., Nissen C., Voderholzer U. (2011). Insomnia as a predictor of depression: a meta-analytic evaluation of longitudinal epidemiological studies. J Affect Disord.

[14] Gregory A.M., Rijsdijk F.V., Lau J.Y., Dahl R.E., Eley T.C. (2009). The direction of longitudinal associations between sleep problems and depression symptoms: a study of twins aged 8 and 10 years. Sleep.

[bib15] Harvey A.G. (2001 Oct). Insomnia: symptom or diagnosis?. Clin Psychol Rev.

[16] Soehner A.M., Kaplan K.A., Harvey A.G. (2013). Insomnia comorbid to severe psychiatric illness. Sleep Med Clin.

[17] Freeman D., Stahl D., McManus S., Meltzer H., Brugha T., Wiles N. (2012 Aug). Insomnia, worry, anxiety and depression as predictors of the occurrence and persistence of paranoid thinking. Soc Psychiatr Psychiatr Epidemiol.

[18] Benca R.M., Obermeyer W.H., Thisted R.A., Gillin J.C. (1992). Sleep and psychiatric disorders: a meta-analysis. Arch Gen Psychiatr.

[19] Breslau N., Roth T., Rosenthal L., Andreski P. (1996). Sleep disturbance and psychiatric disorders: a longitudinal epidemiological study of young adults. Biol Psychiatr.

[20] Chan M.-S., Chung K.-F., Yung K.-P., Yeung W.-F. (2017). Sleep in schizophrenia: a systematic review and meta-analysis of polysomnographic findings in case-control studies. Sleep Med Rev.

[21] Kobayashi I., Boarts J.M., Delahanty D.L. (2007). Polysomnographically measured sleep abnormalities in PTSD: a meta-analytic review. Psychophysiology.

[22] Nota J.A., Sharkey K.M., Coles M.E. (2015). Sleep, arousal, and circadian rhythms in adults with obsessive–compulsive disorder: a meta-analysis. Neurosci Biobehav Rev.

[bib23] Reeve S., Sheaves B., Freeman D. (2015). The role of sleep dysfunction in the occurrence of delusions and hallucinations: a systematic review. Clin Psychol Rev.

[24] Taylor D.J., Lichstein K.L., Durrence H.H., Reidel B.W., Bush A.J. (2005). Epidemiology of insomnia, depression, and anxiety. Sleep.

[25] Harvey A.G., Jones C., Schmidt D.A. (2003). Sleep and posttraumatic stress disorder: a review. Clin Psychol Rev.

[26] Lauer C.J., Krieg J.-C. (2004). Sleep in eating disorders. Sleep Med Rev.

[27] Scott A.J., Rowse G., Webb T.L. (2017). A structural equation model of the relationship between insomnia, negative affect, and paranoid thinking. PLoS One.

[28] Sharafkhaneh A., Giray N., Richardson P., Young T., Hirshkowitz M. (2005). Association of psychiatric disorders and sleep apnea in a large cohort. Sleep.

[29] Wulff K., Gatti S., Wettstein J.G., Foster R.G. (2010). Sleep and circadian rhythm disruption in psychiatric and neurodegenerative disease. Nat Rev Neurosci.

[30] Picchietti D., Winkelman J.W. (2005). Restless legs syndrome, periodic limb movements in sleep, and depression. Sleep.

[31] Dodel R., Peter H., Spottke A., Noelker C., Althaus A., Siebert U. (2007). Health-related quality of life in patients with narcolepsy. Sleep Med.

[32] Plante D.T. (2015). Hypersomnia in mood disorders: a rapidly changing landscape. Curr Sleep Med Rep.

[33] Ohayon M.M., Guilleminault C., Priest R.G. (1999). Night terrors, sleepwalking, and confusional arousals in the general population: their frequency and relationship to other sleep and mental disorders. J Clin Psychiatr.

[34] Hasler B.P., Germain A. (2009). Correlates and treatments of nightmares in adults. Sleep Med Clin.

[35] Brewer M.B., Crano W.D. (2000). Research design and issues of validity. Handb Res Methods Soc Pers Psychol.

[36] Achen C.H. (2005). Let's put garbage-can regressions and garbage-can probits where they belong. Conflict Manag Peace Sci.

[37] Spector P.E., Brannick M.T. (2011). Methodological urban legends: the misuse of statistical control variables. Organ Res Methods.

[38] Zapf D., Dormann C., Frese M. (1996). Longitudinal studies in organizational stress research: a review of the literature with reference to methodological issues. J Occup Health Psychol.

[39] Elwert F., Winship C. (2014). Endogenous selection bias: the problem of conditioning on a collider variable. Annu Rev Sociol.

[40] Morgan S.L., Winship C. (2015).

[41] Pearl J., Glymour M., Jewell N.P. (2016).

[42] Rohrer J.M. (2018). Thinking clearly about correlations and causation: graphical causal models for observational data. Adv Methods Pract Psychol Sci.

[43] Duckworth A.L., Tsukayama E., May H. (2010). Establishing causality using longitudinal hierarchical linear modeling: an illustration predicting achievement from self-control. Soc Psychol Personal Sci.

[44] Woodward J. (2005).

[45] Cartwright N. (2007).

[bib46] Campbell J. (2007). An interventionist approach to causation in psychology. Causal Learn Psychol Philos Comput.

[bib47] Gebara M.A., Siripong N., DiNapoli E.A., Maree R.D., Germain A., Reynolds C.F. (2018). Effect of insomnia treatments on depression: a systematic review and meta-analysis. Depress Anxiety.

[48] Ho F.Y.-Y., Chan C.S., Lo W.-Y., Leung J.C.-Y. (2020). The effect of self-help cognitive behavioral therapy for insomnia on depressive symptoms: an updated meta-analysis of randomized controlled trials. J Affect Disord.

[49] Belleville G., Cousineau H., Levrier K., St-Pierre-Delorme M-È (2011). Meta-analytic review of the impact of cognitive-behavior therapy for insomnia on concomitant anxiety. Clin Psychol Rev.

[50] Gee B., Orchard F., Clarke E., Joy A., Clarke T., Reynolds S. (2019). The effect of non-pharmacological sleep interventions on depression symptoms: a meta-analysis of randomised controlled trials. Sleep Med Rev.

[51] Fedak K.M., Bernal A., Capshaw Z.A., Gross S. (2015). Applying the Bradford Hill criteria in the 21st century: how data integration has changed causal inference in molecular epidemiology. Emerg Themes Epidemiol.

[52] Glanville J.M., Lefebvre C., Miles J.N., Camosso-Stefinovic J. (2006). How to identify randomized controlled trials in MEDLINE: ten years on. J Med Libr Assoc.

[53] Moher D., Liberati A., Tetzlaff J., Altman D.G. (2009 Jul 21). Preferred reporting items for systematic reviews and meta-analyses: the PRISMA statement. BMJ.

[54] Harvey A.G., Stinson K., Whitaker K.L., Moskovitz D., Virk H. (2008). The subjective meaning of sleep quality: a comparison of individuals with and without insomnia. Sleep.

[55] Crivello A., Barsocchi P., Girolami M., Palumbo F. (2019). The meaning of sleep quality: a survey of available technologies. IEEE Access.

[56] Krystal A.D., Edinger J.D. (2008). Measuring sleep quality. Sleep Med.

[57] Libman E., Fichten C., Creti L., Conrod K., Tran D.-L., Grad R. (2016). Refreshing sleep and sleep continuity determine perceived sleep quality. Sleep Disord.

[58] Wu J.Q., Appleman E.R., Salazar R.D., Ong J.C. (2015). Cognitive behavioral therapy for insomnia comorbid with psychiatric and medical conditions: a meta-analysis. JAMA Intern Med.

[59] Strauss J. (2011). Subjectivity and severe psychiatric disorders. Schizophr Bull.

[60] Higgins J.P., Altman D.G., Sterne J.A. (2011). Cochrane handbook for systematic reviews of interventions version 5.1. 0 [updated March 2011].

[61] Team R.C.R. (2013).

[62] Lüdecke Daniel (2019). https://CRAN.R-project.org/package=esc.

[63] Balduzzi S., Rücker G., Schwarzer G. (2019 Nov). How to perform a meta-analysis with R: a practical tutorial. Evid Base Ment Health.

[64] Viechtbauer W. (2010). Conducting meta-analyses in R with the metafor package. J Stat Software.

[65] Harrer M., Cuijpers P., Furukawa T., Ebert D.D. (2019).

[66] McGuinness L.A., Higgins J.P.T. (2020 Apr 26). Risk-of-bias VISualization (robvis): an R package and Shiny web app for visualizing risk-of-bias assessments. Res Synth Methods [Internet].

[67] Borenstein M., Hedges L.V., Higgins J.P., Rothstein H.R. (2010). A basic introduction to fixed-effect and random-effects models for meta-analysis. Res Synth Methods.

[68] Cohen J. (1992). A power primer. Psychol Bull.

[69] Higgins J.P., Thompson S.G., Deeks J.J., Altman D.G. (2003). Measuring inconsistency in meta-analyses. BMJ.

[70] Egger M., Smith G.D., Schneider M., Minder C. (1997). Bias in meta-analysis detected by a simple, graphical test. BMJ.

[71] Orwin R.G. (1983). A fail-safe N for effect size in meta-analysis. J Educ Stat.

[72] Harrer M., Cuijpers P., Furukawa T.A., Ebert D.D. (2019). Doing meta-analysis in R: a hands-on guide. Prot Lab Erlangen.

[73] Scott A., Webb T.L., Martyn-St James M., Rowse G., Weich S. (2021).

[74] Duval S., Tweedie R. (2000). Trim and fill: a simple funnel-plot–based method of testing and adjusting for publication bias in meta-analysis. Biometrics.

[75] Nguyen H., Manolova G., Daskalopoulou C., Vitoratou S., Prince M., Prina A.M. (2019). Prevalence of multimorbidity in community settings: a systematic review and meta-analysis of observational studies. J Comorbidity.

[76] Leucht S., Burkard T., Henderson J.H., Sartorius N., Maj M. (2007).

[77] Sareen J., Cox B.J., Stein M.B., Afifi T.O., Fleet C., Asmundson G.J. (2007). Physical and mental comorbidity, disability, and suicidal behavior associated with posttraumatic stress disorder in a large community sample. Psychosom Med.

[78] Jones D.R., Macias C., Barreira P.J., Fisher W.H., Hargreaves W.A., Harding C.M. (2004). Prevalence, severity, and co-occurrence of chronic physical health problems of persons with serious mental illness. Psychiatr Serv.

[79] El-Gabalawy R., Mackenzie C.S., Shooshtari S., Sareen J. (2011). Comorbid physical health conditions and anxiety disorders: a population-based exploration of prevalence and health outcomes among older adults. Gen Hosp Psychiatr.

[80] Sartorious N. (2013). Comorbidity of mental and physical diseases: a main challenge for medicine of the 21st century. Shanghai Arch Psychiatr.

[81] Espie C.A., Fleming L., Cassidy J., Samuel L., Taylor L.M., White C.A. (2008). Randomized controlled clinical effectiveness trial of cognitive behavior therapy compared with treatment as usual for persistent insomnia in patients with cancer. J Clin Oncol.

[82] Selvanathan J., Pham C., Nagappa M., Peng P.W., Englesakis M., Espie C.A. (2021). Cognitive behavioral therapy for insomnia in patients with chronic pain-A systematic review and meta-analysis of randomized controlled trials. Sleep Med Rev.

[83] Vitiello M.V., Rybarczyk B., Von Korff M., Stepanski E.J. (2009). Cognitive behavioral therapy for insomnia improves sleep and decreases pain in older adults with co-morbid insomnia and osteoarthritis. J Clin Sleep Med.

[84] Kyle S.D., Morgan K., Espie C.A. (2010). Insomnia and health-related quality of life. Sleep Med Rev.

[85] Sampson C.J., Bell E., Cole A., Miller C.B., Rose J. (2021). Digital cognitive behavioural therapy for insomnia and primary care costs in England: an interrupted time series analysis. medRxiv.

[86] Watanabe N. (2014). Cost-effectiveness of brief behavioral therapy for insomnia comorbid with depression: analysis of a randomized controlled trial. Psychosom Med.

[87] Reeve S., Nickless A., Sheaves B., Hodgekins J., Stewart S.L.K., Gumley A. (2019). Sleep duration and psychotic experiences in patients at risk of psychosis: a secondary analysis of the EDIE-2 trial. Schizophr Res.

[88] Van Os J., Linscott R.J., Myin-Germeys I., Delespaul P., Krabbendam L. (2009). A systematic review and meta-analysis of the psychosis continuum: evidence for a psychosis proneness–persistence–impairment model of psychotic disorder. Psychol Med.

[89] Keyes C.L. (2002). The mental health continuum: from languishing to flourishing in life. J Health Soc Behav.

[90] Webb T.L., Sheeran P. (2006). Does changing behavioral intentions engender behavior change? A meta-analysis of the experimental evidence. Psychol Bull.

[91] van der Zweerde T., Bisdounis L., Kyle S.D., Lancee J., van Straten A. (2019). Cognitive behavioral therapy for insomnia: a meta-analysis of long-term effects in controlled studies. Sleep Med Rev.

[92] O'Leary K., Bylsma L.M., Rottenberg J. (2017). Why might poor sleep quality lead to depression? A role for emotion regulation. Cognit Emot.

[93] Gujar N., Yoo S.-S., Hu P., Walker M.P. (2011). Sleep deprivation amplifies reactivity of brain reward networks, biasing the appraisal of positive emotional experiences. J Neurosci.

[94] Zohar D., Tzischinsky O., Epstein R., Lavie P. (2005). The effects of sleep loss on medical residents' emotional reactions to work events: a cognitive-energy model. Sleep.

[95] Zhang J., Lau E.Y.Y., Hsiao J.H. (2019). Using emotion regulation strategies after sleep deprivation: ERP and behavioral findings. Cognit Affect Behav Neurosci.

[96] Vandekerckhove M., Wang Y. (2018). Emotion, emotion regulation and sleep: an intimate relationship. Aims Neurosci.

[97] Palmer C.A., Alfano C.A. (2017). Sleep and emotion regulation: an organizing, integrative review. Sleep Med Rev.

[98] Webb T.L., Schweiger Gallo I., Miles E., Gollwitzer P.M., Sheeran P. (2012). Effective regulation of affect: an action control perspective on emotion regulation. Eur Rev Soc Psychol.

[99] Gratz K.L., Roemer L. (2004 Mar 1). Multidimensional assessment of emotion regulation and dysregulation: development, factor structure, and initial validation of the difficulties in emotion regulation scale. J Psychopathol Behav Assess.

[bib100] Mental Health Foundation (2011).

[bib101] Royal Society for Public Health (2016). https://www.rsph.org.uk/static/uploaded/a565b58a-67d1-4491-ab9112ca414f7ee4.pdf.

[bib102] Freeman D., Sheaves B., Waite F., Harvey A.G., Harrison P.J. (2020 Jul 1). Sleep disturbance and psychiatric disorders. Lancet Psychiatr.

[103] Meaklim H., Jackson M.L., Bartlett D., Saini B., Falloon K., Junge M. (2020). Sleep education for healthcare providers: addressing deficient sleep in Australia and New Zealand. Sleep Health.

[104] Cross E., Ellis J., Draghi-Lorenz R. (2009). The role of sleep and sleep disorders in the therapeutic encounter: an ipa study of counselling psychologists in the UK. Sleep.

[105] Davy Z., Middlemass J., Siriwardena A.N. (2015). Patients' and clinicians' experiences and perceptions of the primary care management of insomnia: qualitative study. Health Expect.

[106] Ellis J. (2012). Sleep and psychology curriculum. Oxf Handb Sleep Disord.

[107] Meltzer L.J., Phillips C., Mindell J.A. (2009). Clinical psychology training in sleep and sleep disorders. J Clin Psychol.

[108] O'Sullivan M., Rahim M., Hall C. (2015). The prevalence and management of poor sleep quality in a secondary care mental health population. J Clin Sleep Med.

[109] Romiszewski S., May F.E.K., Homan E.J., Norris B., Miller M.A., Zeman A. (2020). Medical student education in sleep and its disorders is still meagre 20 years on: a cross-sectional survey of UK undergraduate medical education. J Sleep Res.

[110] Stott R., Pimm J., Emsley R., Miller C.B., Espie C.A. (2021). Does adjunctive digital CBT for insomnia improve clinical outcomes in an improving access to psychological therapies service?. Behav Res Ther.

[111] Kraepelien M., Forsell E., Blom K. (2021). Large-scale implementation of insomnia treatment in routine psychiatric care: patient characteristics and insomnia-depression comorbidity. J Sleep Res.

[112] Kyle S.D., Madigan C., Begum N., Abel L., Armstrong S., Aveyard P. (2020). Primary care treatment of insomnia: study protocol for a pragmatic, multicentre, randomised controlled trial comparing nurse-delivered sleep restriction therapy to sleep hygiene (the HABIT trial). BMJ Open.

[113] Arroll B., Fernando A., Falloon K., Goodyear-Smith F., Samaranayake C., Warman G. (2012). Prevalence of causes of insomnia in primary care: a cross-sectional study. Br J Gen Pract.

[114] Haynes P.L., Parthasarathy S., Kersh B., Bootzin R.R. (2011). Examination of insomnia and insomnia treatment in psychiatric inpatients. Int J Ment Health Nurs.

[115] Waite F., Evans N., Myers E., Startup H., Lister R., Harvey A.G. (2016). The patient experience of sleep problems and their treatment in the context of current delusions and hallucinations. Psychol Psychother Theor Res Pract.

[116] Faulkner S., Bee P. (2016). Perspectives on sleep, sleep problems, and their treatment, in people with serious mental illnesses: a systematic review. PloS One.

[117] Alessi C., Martin J.L., Fiorentino L., Fung C.H., Dzierzewski J.M., Rodriguez Tapia J.C. (2016). Cognitive behavioral therapy for insomnia in older veterans using nonclinician sleep coaches: randomized controlled trial. J Am Geriatr Soc.

[118] Ashworth D.K., Sletten T.L., Junge M., Simpson K., Clarke D., Cunnington D. (2015). A randomized controlled trial of cognitive behavioral therapy for insomnia: an effective treatment for comorbid insomnia and depression. J Counsel Psychol.

[119] Behrendt D., Ebert D.D., Spiegelhalder K., Lehr D. (2020). Efficacy of a self-help web-based recovery training in improving sleep in workers: randomized controlled trial in the general working population. J Med Internet Res.

[120] Bergdahl L., Broman J.E., Berman A.H., Haglund K., von Knorring L., Markstrom A. (2016). Auricular acupuncture and cognitive behavioural therapy for insomnia: a randomised controlled study. Sleep Disord Print.

[121] Blom K., Jernelov S., Ruck C., Lindefors N., Kaldo V. (2017). Three-year follow-up comparing cognitive behavioral therapy for depression to cognitive behavioral therapy for insomnia, for patients with both diagnoses. Sleep.

[122] Cape J., Leibowitz J., Whittington C., Espie C.A., Pilling S. (2016). Group cognitive behavioural treatment for insomnia in primary care: a randomized controlled trial. Psychol Med.

[123] Casault L., Savard J., Ivers H., Savard M.H. (2015). A randomized-controlled trial of an early minimal cognitive-behavioural therapy for insomnia comorbid with cancer. Behav Res Ther.

[124] Chang S.M., Chen C.H. (2016). Effects of an intervention with drinking chamomile tea on sleep quality and depression in sleep disturbed postnatal women: a randomized controlled trial. J Adv Nurs.

[125] Chang Y.L., Chiou A.F., Cheng S.M., Lin K.C. (2016). Tailored educational supportive care programme on sleep quality and psychological distress in patients with heart failure: a randomised controlled trial. Int J Nurs Stud.

[126] Chao L.L., Kanady J.C., Crocker N., Straus L.D., Hlavin J., Metzler T.J. (2021). Cognitive behavioral therapy for insomnia in veterans with gulf war illness: results from a randomized controlled trial. Life Sci.

[127] Chen K.M., Chen M.H., Chao H.C., Hung H.M., Lin H.S., Li C.H. (2009). Sleep quality, depression state, and health status of older adults after silver yoga exercises: cluster randomized trial. Int J Nurs Stud.

[128] Chen I.H., Yeh T.P., Yeh Y.C., Chi M.J., Chen M.W., Chou K.R. (2019). Effects of acupressure on sleep quality and psychological distress in nursing home residents: a randomized controlled trial. J Am Med Dir Assoc.

[129] Cheng P., Kalmbach D.A., Tallent G., Joseph C.L., Espie C.A., Drake C.L. (2019). Depression prevention via digital cognitive behavioral therapy for insomnia: a randomized controlled trial. Sleep.

[bib130] Christensen H., Batterham P.J., Gosling J.A., Ritterband L.M., Griffiths K.M., Thorndike F.P. (2016). Effectiveness of an online insomnia program (SHUTi) for prevention of depressive episodes (the GoodNight Study): a randomised controlled trial. Lancet Psychiatr.

[131] Chung K.F., Yeung W.F., Yu B.Y., Leung F.C., Zhang S.P., Zhang Z.J. (2018). Acupuncture with or without combined auricular acupuncture for insomnia: a randomised, waitlist-controlled trial. Acupunct Med.

[132] Currie S.R. (2000 Feb). Cognitive-behavioural treatment of insomnia secondary to chronic pain. Diss Abstr Int Sect B Sci Eng.

[133] Edinger J.D., Wohlgemuth W.K., Krystal A.D., Rice J.R. (2005). Behavioral insomnia therapy for fibromyalgia patients: a randomized clinical trial. Arch Intern Med.

[134] Espie C.A., Kyle S.D., Miller C.B., Ong J., Hames P., Fleming L. (2014). Attribution, cognition and psychopathology in persistent insomnia disorder: outcome and mediation analysis from a randomized placebo-controlled trial of online cognitive behavioural therapy. Sleep Med.

[135] Espie C.A., Emsley R., Kyle S.D., Gordon C., Drake C.L., Siriwardena A.N. (2019). Effect of digital cognitive behavioral therapy for insomnia on health, psychological well-being, and sleep-related quality of life: a randomized clinical trial. JAMA Psychiatr.

[136] Falloon K., Elley C.R., Fernando A., Lee A.C., Arroll B. (2015). Simplified sleep restriction for insomnia in general practice: a randomised controlled trial. Br J Gen Pract.

[137] Felder J.N., Epel E.S., Neuhaus J., Krystal A.D., Prather A.A. (2020). Efficacy of digital cognitive behavioral therapy for the treatment of insomnia symptoms among pregnant women: a randomized clinical trial. JAMA Psychiatr.

[138] Freeman D., Waite F., Startup H., Myers E., Lister R., McInerney J. (2015). Efficacy of cognitive behavioural therapy for sleep improvement in patients with persistent delusions and hallucinations (BEST): a prospective, assessor-blind, randomised controlled pilot trial. Lancet Psychiatr.

[139] Freeman D., Sheaves B., Goodwin G.M., Yu L.-M., Nickless A., Harrison P.J. (2017). The effects of improving sleep on mental health (OASIS): a randomised controlled trial with mediation analysis. Lancet Psychiatr.

[140] Garland S.N., Carlson L.E., Stephens A.J., Antle M.C., Samuels C., Campbell T.S. (2014). Mindfulness-based stress reduction compared with cognitive behavioral therapy for the treatment of insomnia comorbid with cancer: a randomized, partially blinded, noninferiority trial. J Clin Oncol.

[141] Garland S.N., Xie S.X., DuHamel K., Bao T., Li Q., Barg F.K. (2019). Acupuncture versus cognitive behavioral therapy for insomnia in cancer survivors: a randomized clinical trial. J Natl Cancer Inst.

[142] Germain A., Richardson R., Moul D.E., Mammen O., Haas G., Forman S.D. (2012). Placebo-controlled comparison of prazosin and cognitive-behavioral treatments for sleep disturbances in US Military Veterans. J Psychosom Res.

[143] Glozier N., Christensen H., Griffiths K.M., Hickie I.B., Naismith S.L., Biddle D. (2019 Apr). Adjunctive Internet-delivered cognitive behavioural therapy for insomnia in men with depression: a randomised controlled trial. Aust N Z J Psychiatr.

[144] Ham O.K., Lee B.G., Choi E., Choi S.J. (2020). Efficacy of cognitive behavioral treatment for insomnia: a randomized controlled trial. West J Nurs Res.

[145] Ho F.Y., Chung K.F., Yeung W.F., Ng T.H., Cheng S.K. (2014). Weekly brief phone support in self-help cognitive behavioral therapy for insomnia disorder: relevance to adherence and efficacy. Behav Res Ther.

[146] Irwin M.R., Olmstead R., Carrillo C., Sadeghi N., Breen E.C., Witarama T. (2014). Cognitive behavioral therapy vs. Tai Chi for late life insomnia and inflammatory risk: a randomized controlled comparative efficacy trial. Sleep.

[147] Jansson-Frojmark M., Linton S.J., Flink I.K., Granberg S., Danermark B., Norell-Clarke A. (2012). Cognitive-behavioral therapy for insomnia co-morbid with hearing impairment: a randomized controlled trial. J Clin Psychol Med Settings.

[148] Jernelov S., Lekander M., Blom K., Rydh S., Ljotsson B., Axelsson J. (2012). Efficacy of a behavioral self-help treatment with or without therapist guidance for co-morbid and primary insomnia--a randomized controlled trial. BMC Psychiatr.

[149] Jungquist C.R., Tra Y., Smith M.T., Pigeon W.R., Matteson-Rusby S., Xia Y. (2012). The durability of cognitive behavioral therapy for insomnia in patients with chronic pain. Sleep Disord Print.

[150] Kaldo V., Jernelov S., Blom K., Ljotsson B., Brodin M., Jorgensen M. (2015). Guided internet cognitive behavioral therapy for insomnia compared to a control treatment - a randomized trial. Behav Res Ther.

[151] Kalmbach D.A., Cheng P., Arnedt J.T., Anderson J.R., Roth T., Fellman-Couture C. (2019). Treating insomnia improves depression, maladaptive thinking, and hyperarousal in postmenopausal women: comparing cognitive-behavioral therapy for insomnia (CBTI), sleep restriction therapy, and sleep hygiene education. Sleep Med.

[152] Katofsky I., Backhaus J., Junghanns K., Rumpf H.J., Huppe M., von Eitzen U. (2012). Effectiveness of a cognitive behavioral self-help program for patients with primary insomnia in general practice - a pilot study. Sleep Med.

[153] Kyle S.D., Hurry M.E.D., Emsley R., Marsden A., Omlin X., Juss A. (2020). The effects of digital cognitive behavioral therapy for insomnia on cognitive function: a randomized controlled trial. Sleep.

[154] Lancee J., van den Bout J., van Straten A., Spoormaker V.I. (2012 Jan). Internet-delivered or mailed self-help treatment for insomnia?: a randomized waiting-list controlled trial. Behav Res Ther.

[155] Lancee J., van den Bout J., Sorbi M.J., van Straten A. (2013). Motivational support provided via email improves the effectiveness of internet-delivered self-help treatment for insomnia: a randomized trial. Behav Res Ther.

[156] Lee B., Kim B.K., Kim H.J., Jung I.C., Kim A.R., Park H.J. (2020). Efficacy and safety of electroacupuncture for insomnia disorder: a multicenter, randomized, assessor-blinded, controlled trial. Nat Sci Sleep.

[157] Lichstein K.L., Nau S.D., Wilson N.M., Aguillard R.N., Lester K.W., Bush A.J. (2013). Psychological treatment of hypnotic-dependent insomnia in a primarily older adult sample. Behav Res Ther.

[158] Martinez M.P., Miro E., Sanchez A.I., Diaz-Piedra C., Caliz R., Vlaeyen J.W. (2014). Cognitive-behavioral therapy for insomnia and sleep hygiene in fibromyalgia: a randomized controlled trial. J Behav Med.

[159] McCrae C.S., Williams J., Roditi D., Anderson R., Mundt J.M., Miller M.B. (2019). Cognitive behavioral treatments for insomnia and pain in adults with comorbid chronic insomnia and fibromyalgia: clinical outcomes from the SPIN randomized controlled trial. Sleep.

[160] McCurry S.M., Logsdon R.G., Vitiello M.V., Teri L. (1998). Successful behavioral treatment for reported sleep problems in elderly caregivers of dementia patients: a controlled study. J Gerontol B Psychol Sci Soc Sci.

[161] Nguyen S., McKay A., Wong D., Rajaratnam S.M., Spitz G., Williams G. (2017). Cognitive behavior therapy to treat sleep disturbance and fatigue after traumatic brain injury: a pilot randomized controlled trial. Arch Phys Med Rehabil.

[162] Nguyen S., Wong D., McKay A., Rajaratnam S.M.W., Spitz G., Williams G. (2019). Cognitive behavioural therapy for post-stroke fatigue and sleep disturbance: a pilot randomised controlled trial with blind assessment. Neuropsychol Rehabil.

[163] Norell-Clarke A., Jansson-Frojmark M., Tillfors M., Hollandare F., Engstrom I. (2015). Group cognitive behavioural therapy for insomnia: effects on sleep and depressive symptomatology in a sample with comorbidity. Behav Res Ther.

[164] Park S.D., Yu S.H. (2015). The effects of Nordic and general walking on depression disorder patients' depression, sleep, and body composition. J Phys Ther Sci.

[165] Peoples A.R., Garland S.N., Pigeon W.R., Perlis M.L., Wolf J.R., Heffner K.L. (2019). Cognitive behavioral therapy for insomnia reduces depression in cancer survivors. J Clin Sleep Med.

[166] Raskind M.A., Peterson K., Williams T., Hoff D.J., Hart K., Holmes H. (2013). A trial of prazosin for combat trauma PTSD with nightmares in active-duty soldiers returned from Iraq and Afghanistan. Am J Psychiatr.

[167] Sadler P., McLaren S., Klein B., Harvey J., Jenkins M. (2018). Cognitive behavior therapy for older adults with insomnia and depression: a randomized controlled trial in community mental health services. Sleep.

[168] Sato D., Yoshinaga N., Nagai E., Nagai K., Shimizu E. (2019). Effectiveness of internet-delivered computerized cognitive behavioral therapy for patients with insomnia who remain symptomatic following pharmacotherapy: randomized controlled exploratory trial. J Med Internet Res.

[169] Savard J., Simard S., Ivers H., Morin C.M. (2005). Randomized study on the efficacy of cognitive-behavioral therapy for insomnia secondary to breast cancer, part I: sleep and psychological effects. J Clin Oncol.

[170] Schiller H., Soderstrom M., Lekander M., Rajaleid K., Kecklund G. (2018). A randomized controlled intervention of workplace-based group cognitive behavioral therapy for insomnia. Int Arch Occup Environ Health.

[171] Sheaves B., Freeman D., Isham L., McInerney J., Nickless A., Yu L.M. (2017). Stabilising sleep for patients admitted at acute crisis to a psychiatric hospital (OWLS): an assessor-blind pilot randomised controlled trial. Psychol Med.

[172] Sheaves B., Holmes E.A., Rek S., Taylor K.M., Nickless A., Waite F. (2019). Cognitive behavioural therapy for nightmares for patients with persecutory delusions (nites): an assessor-blind, pilot randomized controlled trial. Can J Psychiatr.

[173] Song M.L., Park K.M., Motamedi G.K., Cho Y.W. (2020). Cognitive behavioral therapy for insomnia in restless legs syndrome patients. Sleep Med.

[174] Tek C., Palmese L.B., Krystal A.D., Srihari V.H., DeGeorge P.C., Reutenauer E.L. (2014). The impact of eszopiclone on sleep and cognition in patients with schizophrenia and insomnia: a double-blind, randomized, placebo-controlled trial. Schizophr Res.

[175] Thiart H., Lehr D., Ebert D.D., Berking M., Riper H. (2015). Log in and breathe out: internet-based recovery training for sleepless employees with work-related strain - results of a randomized controlled trial. Scand J Work Environ Health.

[176] Wagley J. (2010). Efficacy of a brief intervention for insomnia among psychiatric outpatients. Diss Abstr Int Sect B Sci Eng.

[177] Wen X., Wu Q., Liu J., Xu Z., Fan L., Chen X. (2018). Randomized single-blind multicenter trial comparing the effects of standard and augmented acupuncture protocols on sleep quality and depressive symptoms in patients with depression. Psychol Health Med.

[178] Yeung W.F., Chung K.F., Tso K.C., Zhang S.P., Zhang Z.J., Ho L.M. (2011). Electroacupuncture for residual insomnia associated with major depressive disorder: a randomized controlled trial. Sleep.

[179] Zhang L., Tang Y., Hui R., Zheng H., Deng Y., Shi Y. (2020). The effects of active acupuncture and placebo acupuncture on insomnia patients: a randomized controlled trial. Psychol Health Med.

[180] Zhu D., Dai G., Xu D., Xu X., Geng J., Zhu W. (2018). Long-term effects of Tai Chi intervention on sleep and mental health of female individuals with dependence on amphetamine-type stimulants. Front Psychol.

