# Supplementary material for: Improving sleep quality leads to better mental health: A meta-analysis of randomised controlled trials
Source: Sleep Med Rev. 2021 Dec;60:101556. doi: 10.1016/j.smrv.2021.101556 (PMC8651630; doi:10.1016/j.smrv.2021.101556)
Supplement: Supplementary file 1 — Multimedia component 1 [file mmc1.pdf]

Supplementary Materials 1

Example Search Strategies from MEDLINE, Embase, PsychINFO, and The Chochrane Library

MEDLINE

1 *Sleep/ or *Sleep Disorders, Circadian Rhythm/ or *Sleep Disorders, Intrinsic/ or *Narcolepsy/ or *Restless Legs Syndrome/ or *Sleep Apnea Syndromes/ or *"Sleep Initiation and Maintenance Disorders"/ or *Parasomnias/ (50026)

2 (sleep$ or insomnia$ or nightmare$ or hypersomnia$ or parasomnia$ or narcolepsy or circadian rhythm$ or restless leg syndrome or apnea or apnoea).ti. (95569)

3 1 or 2 (104891)

4 *Stress, Psychological/ or *Anxiety Disorders/ or *Obsessive-Compulsive Disorder/ or *Phobic Disorders/ or *"Feeding and Eating Disorders"/ or *Anorexia Nervosa/ or *Binge-Eating Disorder/ or *Bulimia Nervosa/ or *Depressive Disorder/ or *Hallucinations/ or *Delusions/ or *Anxiety/ or *Depression/ or *Psychotic disorders/ (269808)

5 (psychological health or distress or mental or psychiat$ or affect or depress$ or mood or stress or anxious or anxiety or phobi$ or obsessive compulsive disorder$ or OCD or psychos?s or psychotic or schiz$ or bipolar or bi-polar or hallucination$ or delusion$ or eating disorder$ or eating disturbance$ or anorexia or bulimia or binge eating or wellbeing or well-being or QoL or quality of life).ti,ab. (2240175)

6 4 or 5 (2279403)

7 3 and 6 (23138)

8 randomized controlled trial.pt. (456185)

9 controlled clinical trial.pt. (93310)

10 randomized.ab. (395603)

11 placebo.ab. (186506)

12 drug therapy.fs. (1963374)

13 randomly.ab. (275538)

14 trial.ab. (414213)

15 groups.ab. (1697439)

16 or/8-15 (4036167)

17 exp animals/ not humans.sh. (4359144)

18 16 not 17 (3489285)

19 7 and 18 (7165)

****

EMBASE

1 *REM sleep/ or *sleep time/ or *night sleep/ or *REM sleep deprivation/ or *sleep deprivation/ or *sleep quality/ or *sleep waking cycle/ or *sleep/ or *nonREM sleep/ or *slow wave sleep/ or *sleep stage/ or *sleep pattern/ (80556)

2 *circadian rhythm/ (40560)

3 *circadian rhythm sleep disorder/ or *sleep arousal disorder/ or *sleep disorder/ or *"International Classification of Sleep Disorders"/ (19223)

4 *insomnia/ or *primary insomnia/ (14700)

5 *narcolepsy/ (4207)

6 *restless legs syndrome/ (4739)

7 *sleep disordered breathing/ (17731)

8 *parasomnia/ (2165)

9 or/1-8 (159260)

10 (sleep$ or insomnia$ or nightmare$ or hypersomnia$ or parasomnia$ or narcolepsy or circadian rhythm$ or restless leg syndrome or apnea or apnoea).ti. (131816)

11 9 or 10 (188275)

12 *mental stress/ (31138)

13 *anxiety disorder/ (18402)

14 *obsessive compulsive disorder/ (9949)

15 *phobia/ (6400)

16 *eating disorder/ (12239)

17 *anorexia nervosa/ (13369)

18 *bulimia/ or *binge eating disorder/ (9333)

19 *depression/ (131459)

20 *hallucination/ (6359)

21 *delusion/ (5125)

22 *anxiety/ (62434)

23 *psychosis/ (44014)

24 or/12-23 (309044)

25 (psychological health or distress or mental or psychiat$ or affect or depress$ or mood or stress or anxious or anxiety or phobi$ or obsessive compulsive disorder$ or OCD or psychos?s or psychotic or schiz$ or bipolar or bi-polar or hallucination$ or delusion$ or eating disorder$ or eating disturbance$ or anorexia or bulimia or binge eating or wellbeing or well-being or QoL or quality of life).ti,ab. (2823267)

26 24 or 25 (2864273)

27 11 and 26 (44991)

28 random:.tw. (1186456)

29 placebo:.mp. (397027)

30 double-blind:.tw. (178591)

31 or/28-30 (1425736)

32 27 and 31 (6200)

33 limit 32 to embase (2866)

****

PsycINFO

1 *SLEEP WAKE CYCLE/ or *SLEEP ONSET/ or *REM SLEEP/ or *SLEEP DEPRIVATION/ or *NREM SLEEP/ or *SLEEP APNEA/ or *SLEEP/ or *SLEEP DISORDERS/ (30148)

2 *NARCOLEPSY/ (1143)

3 *Restless Leg Syndrome/ (811)

4 *PARASOMNIAS/ (231)

5 *INSOMNIA/ (4299)

6 1 or 2 or 3 or 4 or 5 (34359)

7 (sleep$ or insomnia$ or nightmare$ or hypersomnia$ or parasomnia$ or narcolepsy or circadian rhythm$ or restless leg syndrome or apnea or apnoea).ti. (33274)

8 6 or 7 (38986)

9 *PSYCHOLOGICAL STRESS/ (6528)

10 *Anxiety Disorders/ (12968)

11 *Obsessive Compulsive Disorder/ (10667)

12 *Phobias/ (3907)

13 *Eating Disorders/ (12023)

14 *Anorexia Nervosa/ (8734)

15 *Binge Eating Disorder/ (1024)

16 *Bulimia/ or *"Purging (Eating Disorders)"/ (6139)

17 *Major Depression/ or *"Depression (Emotion)"/ (103683)

18 *HALLUCINATIONS/ (2119)

19 *DELUSIONS/ (3856)

20 *ANXIETY/ (37279)

21 *Psychosis/ (19124)

22 or/7-21 (243050)

23 (psychological health or distress or mental or psychiat$ or affect or depress$ or mood or stress or anxious or anxiety or phobi$ or obsessive compulsive disorder$ or OCD or psychos?s or psychotic or schiz$ or bipolar or bi-polar or hallucination$ or delusion$ or eating disorder$ or eating disturbance$ or anorexia or bulimia or binge eating or wellbeing or well-being or QoL or quality of life).ti,ab. (1111185)

24 22 or 23 (1142651)

25 8 and 24 (38986)

26 double-blind.tw. (20376)

27 random: assigned.tw. (29544)

28 control.tw. (365974)

29 26 or 27 or 28 (399634)

30 25 and 29 (6184)

31 limit 30 to human (5006)

32 25 and 31 (5006)

****

Cochrane

#1 MeSH descriptor: [Sleep] this term only

#2 MeSH descriptor: [Sleep Wake Disorders] this term only

#3 MeSH descriptor: [Narcolepsy] this term only

#4 MeSH descriptor: [Sleep Disorders, Intrinsic] this term only

#5 MeSH descriptor: [Sleep Disorders, Circadian Rhythm] this term only

#6 MeSH descriptor: [Restless Legs Syndrome] this term only

#7 MeSH descriptor: [Sleep Apnea Syndromes] this term only

#8 MeSH descriptor: [Sleep Initiation and Maintenance Disorders] this term only

#9 MeSH descriptor: [Parasomnias] this term only

#10 {or #1-#9}

#11 (sleep* or insomnia* or nightmare* or hypersomnia* or parasomnia* or narcolepsy or circadian rhythm* or restless leg syndrome or apnea or apnoea):ti

#12 #10 or #11

#13 MeSH descriptor: [Stress, Psychological] this term only

#14 MeSH descriptor: [Anxiety Disorders] this term only

#15 MeSH descriptor: [Obsessive-Compulsive Disorder] this term only

#16 MeSH descriptor: [Phobic Disorders] this term only

#17 MeSH descriptor: [Feeding and Eating Disorders] this term only

#18 MeSH descriptor: [Anorexia Nervosa] this term only

#19 MeSH descriptor: [Binge-Eating Disorder] this term only

#20 MeSH descriptor: [Bulimia Nervosa] this term only

#21 MeSH descriptor: [Depressive Disorder] this term only

#22 MeSH descriptor: [Hallucinations] this term only

#23 MeSH descriptor: [Delusions] this term only

#24 MeSH descriptor: [Anxiety] this term only

#25 MeSH descriptor: [Depression] this term only

#26 MeSH descriptor: [Psychotic Disorders] this term only

#27 {or #13-#26}

#28 (psychological health or distress or mental or psychiat* or affect or depress* or mood or stress or anxious or anxiety or phobi* or obsessive compulsive disorder* or OCD or psychos?s or psychotic or schiz* or bipolar or bi-polar or hallucination* or delusion* or eating disorder* or eating disturbance* or anorexia or bulimia or binge eating or wellbeing or well-being or QoL or quality of life):ti

#29 #27 or #28

#30 #12 and #29

****

Search Filters Used

MEDLINE = Cochrane Highly Sensitive Search Strategy for identifying randomized trials in MEDLINE: sensitivity- and precision-maximizing version (2008 revision); Ovid format in Higgins, J.P. and Green, S. eds., 2011. *Cochrane handbook for systematic reviews of interventions* (Vol. 4). John Wiley & Sons.

EMBASE = Best optimization of sensitivity and specificity (Table 3) in Wong, S.S., Wilczynski, N.L. and Haynes, R.B., 2006. Developing optimal search strategies for detecting clinically sound treatment studies in EMBASE. Journal of the Medical Library Association, 94(1), pp.41.

PsycINFO = Best optimization of sensitivity & specificity (Table 3) in Eady, A.M., Wilczynski, N.L., Haynes, R.B. and Hedges Team, 2008. PsycINFO search strategies identified methodologically sound therapy studies and review articles for use by clinicians and researchers. Journal of Clinical Epidemiology, 61(1), pp.34-40.
