# Supplementary material for: Improving sleep quality leads to better mental health: A meta-analysis of randomised controlled trials
Source: Sleep Med Rev. 2021 Dec;60:101556. doi: 10.1016/j.smrv.2021.101556 (PMC8651630; doi:10.1016/j.smrv.2021.101556)
Supplement: Supplementary file 2 — Multimedia component 2 [file mmc2.pdf]

Supplementary Materials 2

Studies that were excluded from the review

1. Abedi P, Nikkhah P. The effect of physical activity based on pedometer on depression, anxiety and insomnia among postmenopausal women: a randomized controlled trial. Maturitas. 2015;81(1):218-.
2. Ahn JH, Kim M, Park S, Jang W, Park J, Oh E, et al. Prolonged-release melatonin in Parkinson's disease patients with a poor sleep quality: A randomized trial. Vol. 75, Parkinsonism & Related Disorders. 2020. p. 50–4.
3. Aibar-Almazán A, Hita-Contreras F, Cruz-Díaz D, de la Torre-Cruz M, Jiménez-García JD, Martínez-Amat A. Effects of Pilates training on sleep quality, anxiety, depression and fatigue in postmenopausal women: a randomized controlled trial. Vol. 124, Maturitas. 2019. p. 62-67.
4. Akbarfahimi M, Nabavi SM, Kor B, Rezaie L, Paschall E. The effectiveness of occupational therapy-based sleep interventions on quality of life and fatigue in patients with multiple sclerosis: a pilot randomized clinical trial study. Vol. 16, Neuropsychiatric disease and treatment. 2020. p. 1369-1379.
5. Al-Sharman A, Khalil H, El-Salem K, Aldughmi M, Aburub A. The effects of aerobic exercise on sleep quality measures and sleep-related biomarkers in individuals with Multiple Sclerosis: A pilot randomised controlled trial. Vol. 45, Neurorehabilitation. 2019. p. 107–15.
6. Alessi CA, Martin JL, Webber AP, Cynthia Kim E, Harker JO, Josephson KR. Randomized, controlled trial of a nonpharmacological intervention to improve abnormal sleep/wake patterns in nursing home residents. Journal of the American Geriatrics Society.

2005;53(5):803–10.

7. Alessi CA, Schnelle JF, MacRae PG, Ouslander JG, al-Samarrai N, Simmons SF, et al. Does physical activity improve sleep in impaired nursing home residents? Journal of the American Geriatrics Society. 1995;43(10):1098–102.

8. Alfaris N, Wadden TA, Sarwer DB, Diwald L, Volger S, Hong P, et al. Effects of a 2-year behavioral weight loss intervention on sleep and mood in obese individuals treated in primary care practice. Obesity. 2015;23(3):558–64.

9. Allain H, Arbus L, Schuck S. Efficacy and safety of zolpidem administered 'as needed' in primary insomnia: Results of a double-blind, placebo-controlled study. Clinical Drug Investigation. 2001;21(6):391–400.

10. Allain H, Le Coz F, Borderies P, Schuck S, De La Giclais B, Patat A, et al. Use of zolpidem 10 mg as a benzodiazepine substitute in 84 patients with insomnia. Human Psychopharmacology. 1998;13(8):551–9.

11. Allen RP, Adler CH, Du W, Butcher A, Bregman DB, Earley CJ. Clinical efficacy and safety of IV ferric carboxymaltose (FCM) treatment of RLS: a multi-centred, placebo-controlled preliminary clinical trial. Sleep Medicine. 2011;12(9):906–13.

12. Alshehri MM, Alenazi AM, Alothman SA, Rucker JL, Phadnis MA, Miles JM, et al. Using Cognitive Behavioral Therapy for Insomnia in People with Type 2 Diabetes, Pilot RCT Part I: Sleep and Concomitant Symptom. Behavioral Sleep Medicine. 2020. p. 1–20.

13. Anonymous. Correction: Anxiety, depression, and insomnia among adults with opioid dependence treated with extended-release naltrexone vs buprenorphine-naloxone: a randomized clinical trial and follow-up study (JAMA Psychiatry (2018) DOI: 10.1001/jamapsychiatry.2018.3537). JAMA Psychiatry. 2019 Feb;76(2):219.

14. Arditte Hall KA, Werner KB, Griffin MG, Galovski TE. The effects of cognitive processing therapy + hypnosis on objective sleep quality in women with posttraumatic stress disorder. Vol. 10, Psychological Trauma:Theory, Pesearch, Practice and Policy. 2020. p. 10.

15. Arias P, Vivas J, Grieve KL, Cudeiro J. Double-blind, randomized, placebo controlled trial on the effect of 10 days low-frequency rTMS over the vertex on sleep in Parkinson's disease. Sleep Medicine. 2010;11(8):759–65.

16. Armitage R, Yonkers K, Cole D, Rush AJ. A multicenter, double-blind comparison of the effects of nefazodone and fluoxetine on sleep architecture and quality of sleep in depressed outpatients. Journal of Clinical Psychopharmacology. 1997;17(3):161–8.

17. Arslan SS, Alemdaroglu I, Karaduman AA, Yilmaz OT. The effects of physical activity on sleep quality, job satisfaction, and quality of life in office workers. Vol. 63, Work. 2019. p. 3–7.

18. Asarnow LD, Bei B, Krystal A, Buysse DJ, Thase ME, Edinger JD, et al. Circadian Preference as a Moderator of Depression Outcome Following Cognitive Behavioral Therapy for Insomnia Plus Antidepressant Medications: A Report From the TRIAD Study. Journal of Clinical Sleep Medicine. 2019;15(4):573–80.

19. Asnis GM, Chakraburtty A, DuBoff EA, Krystal A, Londborg PD, Rosenberg R, et al. Zolpidem for persistent insomnia in SSRI-treated depressed patients. Journal of Clinical Psychiatry. 1999;60(10):668–76.

20. Atlantis E, Chow CM, Kirby A, Singh MAF. Worksite intervention effects on sleep quality: a randomized controlled trial. Journal of Occupational Health Psychology. 2006;11(4):291–304.

21. Ayalon L, Ancoli-Israel S, Stepnowsky C, Marler M, Palmer BW, Liu L, et al. Adherence to continuous positive airway pressure treatment in patients with Alzheimer's disease and obstructive sleep apnea. American Journal of Geriatric Psychiatry. 2006;14(2):176–80.

22. Baek Y, Kim H, Mun S, Lee S. Three-Component Herbal Tea Alleviates Prolonged Fatigue and Improves Sleep Quality: A Randomized Controlled Pilot Study. Explore: The Journal of Science & Healing. 2018;14(6):420–3.

23. Balcan B, Thunström E, Strollo PJ, Peker Y. Continuous Positive Airway Pressure Treatment and Depression in Adults with Coronary Artery Disease and Nonsleepy Obstructive Sleep Apnea. A Secondary Analysis of the RICCADSA Trial. Vol. 16, Annals of the american thoracic society. 2019. p. 62-70.

24. Barabasz AF. Treatment of insomnia in depressed patients by hypnosis and cerebral electrotherapy. American Journal of Clinical Hypnosis. 1976;19(2):120–2.

25. Barnes CM, Miller JA, Bostock S. Helping employees sleep well: Effects of cognitive behavioral therapy for insomnia on work outcomes. Journal of Applied Psychology. 2017;102(1):104–13.

26. Barton DL, Atherton PJ, Bauer BA, Moore DF Jr, Mattar BI, Lavasseur BI, et al. The use of Valeriana officinalis (Valerian) in improving sleep in patients who are undergoing treatment for cancer: a phase III randomized, placebo-controlled, double-blind study (NCCTG Trial, N01C5). The Journal of Supportive Oncology. 2011;9(1):24–31.

27. Becker PM, Schwartz JR, Feldman NT, Hughes RJ. Effect of modafinil on fatigue, mood, and health-related quality of life in patients with narcolepsy. Psychopharmacology. 2004;171(2):133–9.

28. Belanger L, Harvey AG, Fortier-Brochu E, Beaulieu-Bonneau S, Eidelman P, Talbot L, et

al. Impact of comorbid anxiety and depressive disorders on treatment response to cognitive behavior therapy for insomnia. Journal of Consulting & Clinical Psychology. 2016;84(8):659–67.

29. Bell KR, Fogelberg D, Barber J, Nakase-Richardson R, Zumsteg JM, Dubiel R, et al. The effect of phototherapy on sleep during acute rehabilitation after traumatic brain injury: a randomized controlled trial. Brain Injury. 2021. p. 1–9.

30. Belleville G, Guay C, Guay B, Morin CM. Hypnotic taper with or without self-help treatment of insomnia: a randomized clinical trial. Journal of consulting and clinical psychology. 2007;75(2):325-335.

31. Berg LM, Ankjell TKS, Sun YQ, Trovik TA, Rikardsen OG, Sjogren A, et al. Health-Related Quality of Life and Sleep Quality after 12 Months of Treatment in Nonsevere Obstructive Sleep Apnea: A Randomized Clinical Trial with Continuous Positive Airway Pressure and Mandibular Advancement Splints. Vol. 2020, International journal of otolaryngology. 2020. p. 2856460.

32. Berg SK, Higgins M, Reilly CM, Langberg JJ, Dunbar SB. Sleep quality and sleepiness in persons with implantable cardioverter defibrillators: outcome from a clinical randomized longitudinal trial. Pacing & Clinical Electrophysiology. 2012;35(4):431–43.

33. Bergdahl L, Broman JE, Berman AH, Haglund K, von Knorring L, Markstrom A. Sleep patterns in a randomized controlled trial of auricular acupuncture and cognitive behavioral therapy for insomnia. Complementary Therapies in Clinical Practice. 2017;28:220–6.

34. Bernstein AM, Allexandre D, Bena J, Doyle J, Gendy G, Wang L, et al. ‘Go! to Sleep’: A Web-Based Therapy for Insomnia. Telemedicine Journal & E-Health. 2017;23(7):590–9.

35. Berry R, Verrier MJ, Rashiq S, Simmonds MK, Baloukov A, Thottungal J, et al. A brief

cognitive-behavioral intervention for sleep in individuals with chronic noncancer pain: A randomized controlled trial. Rehabilitation Psychology. 2015;60(2):193–200.

36. Bhati SR. The effect of the Sleep Support for Moms Intervention on postpartum sleep and depressive symptoms. a pilot Randomized Controlled Trial. Dissertation Abstracts International: Section B: The Sciences and Engineering. 2015;76(2-B(E)):No Pagination Specified.

37. Bigelman KA, Chapman DP, Freese EC, Trilk JL, Cureton KJ. Effects of 6 weeks of quercetin supplementation on energy, fatigue, and sleep in ROTC cadets. Military Medicine. 2011;176(5):565–72.

38. Bishop TM, Crean HF, Funderburk JS, Pigeon WR. Initial Session Effects of Brief Cognitive Behavioral Therapy for Insomnia: A Secondary Analysis of A Small Randomized Pilot Trial. Behavioral Sleep Medicine. 2021. p. 1–14.

39. Black JE, Hirshkowitz M. Modafinil for treatment of residual excessive sleepiness in nasal continuous positive airway pressure-treated obstructive sleep apnea/hypopnea syndrome. Sleep. 2005;28(4):464–71.

40. Blackburn L, Achor S, Allen B, Bauchmire N, Dunnington D, Klisovic RB, et al. The Effect of Aromatherapy on Insomnia and Other Common Symptoms Among Patients With Acute Leukemia. Oncology Nursing Forum. 2017;44(4):E185–93.

41. Blom K, Jernelöv S, Kraepelien M, Lindefors N, Kaldo V. Is insomnia treatment superior to depression treatment for patients with both diagnoses?-Results of a randomized controlled trial. Sleep medicine. 2013;14:e38-e39.

42. Blom K, Jernelov S, Kraepelien M, Bergdahl MO, Jungmarker K, Ankartjarn L, et al. Internet treatment addressing either insomnia or depression, for patients with both

diagnoses: a randomized trial. Sleep. 2015;38(2):267–77.

43. Blom K, Jernelov S, Ruck C, Lindefors N, Kaldo V. Three-Year Follow-Up of Insomnia and Hypnotics after Controlled Internet Treatment for Insomnia. Sleep. 2016;39(6):1267–74.

44. Blom K, Tarkian Tillgren H, Wiklund T, Danlycke E, Forssen M, Soderstrom A, et al. Internet-vs. group-delivered cognitive behavior therapy for insomnia: A randomized controlled non-inferiority trial. Behaviour Research & Therapy. 2015;70:47–55.

45. Blytt KM, Bjorvatn B, Husebo B, Flo E. Effects of pain treatment on sleep in nursing home patients with dementia and depression: a multicenter placebo-controlled randomized clinical trial. International journal of geriatric psychiatry [Internet]. 2017;(no pagination). Available from: https://www.cochranelibrary.com/central/doi/10.1002/central/CN-01440908/fullhttps://onlinelibrary.wiley.com/doi/pdf/10.1002/gps.4839 https://onlinelibrary.wiley.com/doi/full/10.1002/gps.4839

46. Blytt KM, Bjorvatn B, Husebo B, Flo E. Effects of pain treatment on sleep in nursing home patients with dementia and depression: A multicenter placebo-controlled randomized clinical trial. International Journal of Geriatric Psychiatry. 2018;33(4):663–70.

47. Blytt KM, Husebo B, Flo E, Bjorvatn B. Long-term pain treatment did not improve sleep in nursing home patients with comorbid dementia and depression: A 13-week randomized placebo-controlled trial. Frontiers in Psychology Vol 9 2018, ArtID 134 [Internet]. 2018 Feb;9. Available from: http://ovidsp.ovid.com/ovidweb.cgi?T=JS&CSC=Y&NEWS=N&PAGE=fulltext&D=psy

c14&AN=2018-07405-001

48. Bogan RK, Fry JM, Schmidt MH, Carson SW, Ritchie SY, Group TRUS. Ropinirole in the treatment of patients with restless legs syndrome: a US-based randomized, double-blind, placebo-controlled clinical trial. Mayo Clinic Proceedings. 2006;81(1):17–27.

49. Bollu V, Bushmakin AG, Cappelleri JC, Chen CC, Feltner D, Wittchen HU. Pregabalin reduces sleep disturbance in patients with generalized anxiety disorder via both direct and indirect mechanisms. European Journal of Psychiatry. 2010 Jan;24(1):18–27.

50. Bonin EM, Beecham J, Swift N, Raikundalia S, Brown JS. Psycho-educational CBT-Insomnia workshops in the community. A cost-effectiveness analysis alongside a randomised controlled trial. Behaviour Research & Therapy. 2014;55:40–7.

51. Bonn SE, Löf M, Östenson CG, Trolle Lagerros Y. App-technology to improve lifestyle behaviors among working adults - the Health Integrator study, a randomized controlled trial. Vol. 19, BMC public health. 2019. p. 273.

52. Bosch P, van den Noort M, Staudte H, Lim S, Yeo S, Coenen A, et al. Sleep disorders in patients with depression or schizophrenia: A randomized controlled trial using acupuncture treatment. European Journal of Integrative Medicine. 2016 Oct;8(5):789–96.

53. Bosch P, van Luijtelaar G, van den Noort M, Lim S, Egger J, Coenen A. Sleep ameliorating effects of acupuncture in a psychiatric population. Evidence-Based Complementary & Alternative Medicine: eCAM. 2013;2013:969032.

54. Bramoweth AD, Lederer LG, Youk AO, Germain A, Chinman MJ. Brief Behavioral Treatment for Insomnia vs. Cognitive Behavioral Therapy for Insomnia: Results of a Randomized Noninferiority Clinical Trial Among Veterans. Vol. 51, Behavior Therapy.

2020. p. 535–47.

55. Brandao GS, Gomes G, Callou Sampaio AA, Donner CF, Oliveira LVF, Camelier AA. Home exercise improves the quality of sleep and daytime sleepiness of elderlies: a randomized controlled trial. Multidisciplinary Respiratory Medicine. 2018;13:2.

56. Broomfield NM, Espie CA. Initial insomnia and paradoxical intention: An experimental investigation of putative mechanisms using subjective and actigraphic measurement of sleep. Behavioural and Cognitive Psychotherapy. 2003 Jul;31(3):313–24.

57. Buman MP, Hekler EB, Bliwise DL, King AC. Moderators and mediators of exercise-induced objective sleep improvements in midlife and older adults with sleep complaints. Health Psychology. 2011;30(5):579–87.

58. Burkhalter H, Wirz-Justice A, Denhaerynck K, Fehr T, Steiger J, Venzin RM, et al. The effect of bright light therapy on sleep and circadian rhythms in renal transplant recipients: a pilot randomized, multicentre wait-list controlled trial. Transplant International. 2015;28(1):59–70.

59. Burkhart K, Phelps JR. Amber lenses to block blue light and improve sleep: a randomized trial. Chronobiology International. 2009;26(8):1602–12.

60. Burns JW, Gerhart J, Rizvydeen M, Kimura M, Burgess HJ. Morning Bright Light Treatment for Chronic Low Back Pain: Potential Impact on the Volatility of Pain, Mood, Function, and Sleep. Vol. 21, Pain Medicine. 2020. p. 1153–61.

61. Canazei M, Bassa D, Jimenez P, Papousek I, Fink A, Weiss E. Effects of an adjunctive, chronotype-based light therapy in hospitalized patients with severe burnout symptoms - a pilot study. Vol. 36, Chronobiology international. 2019. p. 993-1004.

62. Carney CE, Edinger JD, Kuchibhatla M, Lachowski AM, Bogouslavsky O, Krystal AD, et

al. Cognitive behavioral insomnia therapy for those with insomnia and depression: A randomized controlled clinical trial. Sleep: Journal of Sleep and Sleep Disorders Research. 2017 Apr;40(4):1–13.

63. Carter PA. A brief behavioral sleep intervention for family caregivers of persons with cancer. Cancer Nursing. 2006;29(2):95–103.

64. Casement MD, Livingston NR, Allen NB, Forbes EE. Feasibility, acceptability and affective consequences of at-home sleep extension in young women with depressive symptoms: a pilot study [Internet]. Journal of sleep research. 2020. Available from: https://www.cochranelibrary.com/central/doi/10.1002/central/CN-02130981/full

65. Castro Sánchez AM, García López H, Fernández Sánchez M, Pérez Mármol JM, Aguilar-Ferrándiz ME, Luque Suárez A, et al. Improvement in clinical outcomes after dry needling versus myofascial release on pain pressure thresholds, quality of life, fatigue, pain intensity, quality of sleep, anxiety, and depression in patients with fibromyalgia syndrome. Vol. 41, Disability and rehabilitation. 2019. p. 2235-2246.

66. Celik Y, Thunstrom E, Strollo PJ, Peker Y. Continuous positive airway pressure treatment and anxiety in adults with coronary artery disease and nonsleepy obstructive sleep apnea in the RICCADSA trial. Vol. 77, Sleep medicine. 2021. p. 96-103.

67. Chakravorty S, Morales KH, Arnedt JT, Perlis ML, Oslin DW, Findley JC, et al. Cognitive Behavioral Therapy for Insomnia in Alcohol Dependent Veterans: A Randomized, Controlled Pilot Study. Alcoholism: Clinical & Experimental Research. 2019;26:26.

68. Chan AW, Yu DS, Choi KC, Lee DT, Sit JW, Chan HY. Tai chi qigong as a means to improve night-time sleep quality among older adults with cognitive impairment: a pilot

randomized controlled trial. Clinical Interventions In Aging. 2016;11:1277–86.

69. Chan MF, Chan EA, Mok E. Effects of music on depression and sleep quality in elderly people: A randomised controlled trial. Complementary Therapies in Medicine. 2010;18(3–4):150–9.

70. Chapoutot M, Peter-Derex L, Schoendorff B, Faivre T, Bastuji H, Putois B. Telehealth-delivered CBT-I programme enhanced by acceptance and commitment therapy for insomnia and hypnotic dependence: A pilot randomized controlled trial. Vol. 30, Journal of Sleep Research. 2021. p. e13199.

71. Chen CK, Lin YC, Cheng JW, Pei YC, Liu GH, Chen YL, et al. Effectiveness of Laser Acupuncture in Alleviating Chronic Insomnia: A Single-Blinded Randomized Controlled Trial. Vol. 2019, Evidence-Based Complementary & Alternative Medicine: eCAM. 2019. p. 8136967.

72. Chen HM, Tsai CM, Wu YC, Lin KC, Lin CC. Effect of walking on circadian rhythms and sleep quality of patients with lung cancer: a randomised controlled trial. British Journal of Cancer. 2016;115(11):1304–12.

73. Chen KM, Huang HT, Cheng YY, Li CH, Chang YH. Sleep quality and depression of nursing home older adults in wheelchairs after exercises. Nursing Outlook. 2015;63(3):357–65.

74. Chen LJ, Stevinson C, Fang SH, Taun CY, Ku PW. Effects of an Acute Bout of Light-Intensity Walking on Sleep in Older Women With Sleep Impairment: A Randomized Controlled Trial. Journal of Clinical Sleep Medicine. 2019;15(4):581–6.

75. Chen MC, Liu HE, Huang HY, Chiou AF. The effect of a simple traditional exercise programme (Baduanjin exercise) on sleep quality of older adults: a randomized controlled

trial. International Journal of Nursing Studies. 2012;49(3):265–73.

76. Cheng K, Ang NKE, Chan N, Piper B. The effects of home-based symptom management programme to fatigue, sleep disturbance, and mood disturbance in patients with cancer undergoing adjuvant chemotherapy. Supportive care in cancer. 2013;21:S24.

77. Cheng P, Kalmbach D, Fellman-Couture C, Arnedt JT, Cuamatzi-Castelan A, Drake CL. Risk of excessive sleepiness in sleep restriction therapy and cognitive behavioral therapy for insomnia: a randomized controlled trial. Vol. 16, Journal of Clinical Sleep Medicine. 2020. p. 193–8.

78. Cheng P, Luik AI, Fellman-Couture C, Peterson E, Joseph CLM, Tallent G, et al. Efficacy of digital CBT for insomnia to reduce depression across demographic groups: a randomized trial. Psychological Medicine. 2019;49(3):491–500.

79. Cheng P, Luik AI, Fellman-Couture C, Peterson E, Joseph CLM, Tallent G, et al. Efficacy of digital CBT for insomnia to reduce depression across demographic groups: a randomized trial. Vol. 49, Psychological medicine. 2019. p. 491-500.

80. Cheville AL, Kollasch J, Vandenberg J, Shen T, Grothey A, Gamble G, et al. A home-based exercise program to improve function, fatigue, and sleep quality in patients with Stage IV lung and colorectal cancer: a randomized controlled trial. Journal of Pain & Symptom Management. 2013;45(5):811–21.

81. Chien HC, Chung YC, Yeh ML, Lee JF. Breathing exercise combined with cognitive behavioural intervention improves sleep quality and heart rate variability in major depression. Journal of Clinical Nursing. 2015;24(21–22):3206–14.

82. Chun N, Kim M, Noh GO. Effects of a Sleep Improvement Program Combined with Aroma-Necklace on Sleep, Depression, Anxiety and Blood Pressure in Elderly Women.

Journal of Korean Academy of Nursing. 2017;47(5):651–62.

83. Chung KF, Yeung WF, Yu YM, Yung KP, Zhang SP, Zhang ZJ, et al. Acupuncture for residual insomnia associated with major depressive disorder: a placebo- and sham-controlled, subject- and assessor-blind, randomized trial. Journal of Clinical Psychiatry. 2015;76(6):e752-760.

84. Clarke G, McGlinchey EL, Hein K, Gullion CM, Dickerson JF, Leo MC, et al. Cognitive-behavioral treatment of insomnia and depression in adolescents: A pilot randomized trial. Behaviour Research & Therapy. 2015;69:111–8.

85. Cohen L, Warneke C, Fouladi RT, Rodriguez MA, Chaoul-Reich A. Psychological adjustment and sleep quality in a randomized trial of the effects of a Tibetan yoga intervention in patients with lymphoma. Cancer. 2004;100(10):2253–60.

86. Cook JM, Harb GC, Gehrman PR, Cary MS, Gamble GM, Forbes D, et al. Imagery rehearsal for posttraumatic nightmares: a randomized controlled trial. Journal of Traumatic Stress. 2010;23(5):553–63.

87. Correa HL, Moura SRG, Neves RVP, Tzanno-Martins C, Souza MK, Haro AS, et al. Resistance training improves sleep quality, redox balance and inflammatory profile in maintenance hemodialysis patients: a randomized controlled trial. Vol. 10, Scientific Reports. 2020. p. 11708.

88. Creti L, Libman E, Bailes S, Fichten CS. Effectiveness of Cognitive-Behavioral Insomnia Treatment in a Community Sample of Older Individuals: More Questions than Conclusions. Journal of Clinical Psychology in Medical Settings. 2005 Jun;12(2):153–64.

89. D'Aurea CVR, Poyares D, Passos GS, Santana MG, Youngstedt SD, Souza AA, et al. Effects of resistance exercise training and stretching on chronic insomnia. Revista

Brasileira de Psiquiatria. 2019;41(1):51–7.

90. Datto C, Svedsater H, Locklear JC, Endicott J. Effect of extended-release quetiapine fumarate on quality of life and sleep in elderly patients with generalized anxiety disorder. Neuropsychiatry. 2013 Dec;3(6):577–85.

91. Davidson JR, Dawson S, Krsmanovic A. Effectiveness of Group Cognitive Behavioral Therapy for Insomnia (CBT-I) in a Primary Care Setting. Behavioral Sleep Medicine. 2017;1–13.

92. Davis JL, Rhudy JL, Pruiksma KE, Byrd P, Williams AE, McCabe KM, et al. Physiological predictors of response to exposure, relaxation, and rescripting therapy for chronic nightmares in a randomized clinical trial. Journal of Clinical Sleep Medicine. 2011;7(6):622–31.

93. Davis JL, Wright DC. Randomized clinical trial for treatment of chronic nightmares in trauma-exposed adults. Journal of Traumatic Stress. 2007;20(2):123–33.

94. Dean GE, Weiss C, Jungquist CR, Klimpt ML, Alameri R, Ziegler PA, et al. Nurse-Delivered Brief Behavioral Treatment for Insomnia in Lung Cancer Survivors: A Pilot RCT. Vol. 18, Behavioral Sleep Medicine. 2020. p. 774–86.

95. DeBattista C, Doghramji K, Menza MA, Rosenthal MH, Fieve RR, Modafinil in Depression Study G. Adjunct modafinil for the short-term treatment of fatigue and sleepiness in patients with major depressive disorder: a preliminary double-blind, placebo-controlled study. Journal of Clinical Psychiatry. 2003;64(9):1057–64.

96. Declerck A, Smits M. Zolpidem, a valuable alternative to benzodiazepine hypnotics for chronic insomnia? Journal of International Medical Research. 1999;27(6):253–63.

97. Denis D, Eley TC, Rijsdijk F, Zavos HMS, Keers R, Espie CA, et al. Is digital cognitive

behavioural therapy for insomnia effective in treating sub-threshold insomnia: a pilot RCT. Vol. 66, Sleep Medicine. 2020. p. 174–83.

98. Deshpande A, Irani N, Balkrishnan R, Benny IR. A randomized, double blind, placebo controlled study to evaluate the effects of ashwagandha (Withania somnifera) extract on sleep quality in healthy adults. Vol. 72, Sleep Medicine. 2020. p. 28–36.

99. Diaz-Alonso J, Smith-Plaza AM, Suarez-Mier B, Lana A. Impact of a Nurse Intervention to Improve Sleep Quality in Intensive Care Units: Results From a Randomized Controlled Trial. DCCN - Dimensions of Critical Care Nursing. 2018;37(6):310–7.

100. Dixon S, Morgan K, Mathers N, Thompson J, Tomeny M. Impact of cognitive behavior therapy on health-related quality of life among adult hypnotic users with chronic insomnia. Behavioral Sleep Medicine. 2006;4(2):71–84.

101. Dockhorn RJ, Dockhorn DW. Zolpidem in the treatment of short-term insomnia: a randomized, double-blind, placebo-controlled clinical trial. Clinical Neuropharmacology. 1996;19(4):333–40.

102. Dolberg OT, Hirschmann S, Grunhaus L. Melatonin for the treatment of sleep disturbances in major depressive disorder. American Journal of Psychiatry. 1998;155(8):1119–21.

103. Dozeman E, van Straten A, van Genugten CR, van Schaik DJF. [Guided web-based intervention for sleeping problems (I-Sleep) in depressed patients within outpatient specialist mental health care: a pilot study]. Vol. 61, Tijdschrift voor Psychiatrie. 2019. p. 683–91.

104. Ebert DD, Berking M, Thiart H, Riper H, Laferton JAC, Cuijpers P, et al. Restoring depleted resources: Efficacy and mechanisms of change of an internet-based unguided

recovery training for better sleep and psychological detachment from work. Health Psychology. 2015;34S:1240–51.

105. Edinger JD, Olsen MK, Stechuchak KM, Means MK, Lineberger MD, Kirby A, et al. Cognitive behavioral therapy for patients with primary insomnia or insomnia associated predominantly with mixed psychiatric disorders: a randomized clinical trial. Sleep. 2009;32(4):499–510.

106. Edinger JD, Wohlgemuth WK, Radtke RA, Marsh GR, Quillian RE. Cognitive behavioral therapy for treatment of chronic primary insomnia: a randomized controlled trial. Jama. 2001;285(14):1856–64.

107. Edinger JD, Wohlgemuth WK, Radtke RA, Coffman CJ, Carney CE. Dose-response effects of cognitive-behavioral insomnia therapy: a randomized clinical trial. Sleep. 2007;30(2):203–12.

108. Efe Arslan D, Kilic Akca N. The effect of aromatherapy hand massage on distress and sleep quality in hemodialysis patients: a randomized controlled trial. Vol. 39, Complementary therapies in clinical practice. 2020. p. 101136-.

109. Ehrampoush MH, Tabei SZ, Mahmoodabad SSM, Fallahzadeh H, Nami M, Khayer E, et al. A study of comparing two cognitive-behavioral workshop for college students: Sleep, wakefulness program and perseverance program. Vol. 8, Journal of Family Medicine & Primary Care. 2019. p. 1222–6.

110. Ekholm B, Spulber S, Adler M. A randomized controlled study of weighted chain blankets for insomnia in psychiatric disorders [Internet]. Journal of clinical sleep medicine. 2020. Available from: https://www.cochranelibrary.com/central/doi/10.1002/central/CN-02140044/full

111. El Rafihi-Ferreira R, Morin CM, Toscanini AC, Lotufo Neto F, Brasil IS, Gallinaro JG, et al. Acceptance and commitment therapy-based behavioral intervention for insomnia: a pilot randomized controlled trial. Vol. 18, Revista Brasileira de Psiquiatria. 2020. p. 18.

112. Ellis JG, De Koninck J, Bastien CH. Managing Insomnia Using Lucid Dreaming Training: A Pilot Study. Vol. 19, Behavioral Sleep Medicine. 2021. p. 273–83.

113. Espie CA, MacMahon KM, Kelly HL, Broomfield NM, Douglas NJ, Engleman HM, et al. Randomized clinical effectiveness trial of nurse-administered small-group cognitive behavior therapy for persistent insomnia in general practice. Sleep. 2007;30(5):574–84.

114. Fabre LF Jr, Gainey A, Kemple S, McLendon DM, Metzler CM. Pilot open-label study of triazolam in the treatment of insomnia following alcohol withdrawal. Journal of Studies on Alcohol. 1977;38(11):2188–92.

115. Fan B, Song W, Zhang J, Er Y, Xie B, Zhang H, et al. The efficacy of mind-body (Baduanjin) exercise on self-reported sleep quality and quality of life in elderly subjects with sleep disturbances: a randomized controlled trial. Vol. 24, Sleep & Breathing. 2020. p. 695–701.

116. Fava M, Asnis GM, Shrivastava R, Lydiard B, Bastani B, Sheehan D, et al. Zolpidem extended-release improves sleep and next-day symptoms in comorbid insomnia and generalized anxiety disorder. Journal of Clinical Psychopharmacology. 2009;29(3):222–30.

117. Fava M, Asnis GM, Shrivastava RK, Lydiard B, Bastani B, Sheehan DV. Improved Insomnia Symptoms and Sleep-Related Next-Day Functioning in Patients With Comorbid Major Depressive Disorder and Insomnia Following Concomitant Zolpidem Extended-Release 12.5 mg and Escitalopram Treatment: a Randomized Controlled Trial.

Journal of clinical psychiatry. 2011;72(7):914-928.

118. Fava M, McCall WV, Krystal A, Wessel T, Rubens R, Caron J, et al. Eszopiclone co-administered with fluoxetine in patients with insomnia coexisting with major depressive disorder. Biological Psychiatry. 2006;59(11):1052–60.

119. Felder JN, Epel ES, Neuhaus J, Krystal AD, Prather AA. Efficacy of Digital Cognitive Behavioral Therapy for the Treatment of Insomnia Symptoms Among Pregnant Women: A Randomized Clinical Trial. Vol. 77, JAMA Psychiatry. 2020. p. 484–92.

120. Ferguson JM, Bielski RJ, Houston J, Post GL, Crowder J, Bailey L, et al. Comparison of estazolam and placebo in the outpatient treatment of insomnia associated with major depression. Current Therapeutic Research - Clinical and Experimental. 1991;49(5):898–907.

121. Ferguson JM, Mendels J, Schwart GE. Effects of reboxetine on Hamilton Depression Rating Scale factors from randomized, placebo-controlled trials in major depression. International clinical psychopharmacology. 2002;17(2):45-51.

122. Ferini-Strambi L, Aarskog D, Partinen M, Chaudhuri KR, Sohr M, Verri D, et al. Effect of pramipexole on RLS symptoms and sleep: a randomized, double-blind, placebo-controlled trial. Sleep Medicine. 2008;9(8):874–81.

123. Feuerstein S, Hodges SE, Keenaghan B, Bessette A, Forselius E, Morgan PT. Computerized Cognitive Behavioral Therapy for Insomnia in a Community Health Setting. Journal of Clinical Sleep Medicine. 2017;13(2):267–74.

124. Friedrich A, Clasen M, Schlarb AA. Sleep better, feel better? Effects of a CBT-I and HT-I sleep training on mental health, quality of life and stress coping in university students: a randomized pilot controlled trial. BMC Psychiatry. 2018;18(1):268.

125. Frisk J, Kallstrom AC, Wall N, Fredrikson M, Hammar M. Acupuncture improves health-related quality-of-life (HRQoL) and sleep in women with breast cancer and hot flushes. Supportive Care in Cancer. 2012;20(4):715–24.

126. Galovski TE, Harik JM, Blain LM, Elwood L, Gloth C, Fletcher TD. Augmenting cognitive processing therapy to improve sleep impairment in PTSD: A randomized controlled trial. Journal of Consulting & Clinical Psychology. 2016;84(2):167–77.

127. Gammoh OS, Al-Smadi A, Mukattash T, Al-Katib W, Attarian H, Al-Shawagfeh M. Efficacy of single dose antihistamine vs. single dose valerian-hops in subjective sleep measures among war refugees: A comparison trial. Revista de Psiquiatria Clinica. 2017;44(2):35–9.

128. Garland SN, Rouleau CR, Campbell T, Samuels C, Carlson LE. The Comparative Impact of Mindfulness-Based Cancer Recovery (MBCR) and Cognitive Behavior Therapy for Insomnia (CBT-I) on Sleep and Mindfulness in Cancer Patients. Explore: The Journal of Science & Healing. 2015;11(6):445–54.

129. Gebara MA, Di Napoli E, Kasckow J, Karp J. Brief behavioral treatmnet for insomnia in older veterans with late life treatment resistant depression. American journal of geriatric psychiatry. 2018;26(3):S131-.

130. Gehrman PR, Connor DJ, Martin JL, Shochat T, Corey-Bloom J, Ancoli-Israel S. Melatonin fails to improve sleep or agitation in double-blind randomized placebo-controlled trial of institutionalized patients with alzheimer disease. American Journal of Geriatric Psychiatry. 2009 Feb;17(2):166–9.

131. Gerber M, Minghetti A, Beck J, Zahner L, Donath L. Is improved fitness following a 12-week exercise program associated with decreased symptom severity, better wellbeing,

and fewer sleep complaints in patients with major depressive disorders? A secondary analysis of a randomized controlled trial. Journal of Psychiatric Research. 2019 Jun;113:58–64.

132. Germain A, Richardson R, Stocker R, Mammen O, Hall M, Bramoweth AD, et al. Treatment for insomnia in combat-exposed OEF/OIF/OND military veterans: preliminary randomized controlled trial. Behaviour Research & Therapy. 2014;61:78–88.

133. Ghazal A, Sorichter S, Jonas I, Rose EC. A randomized prospective long-term study of two oral appliances for sleep apnoea treatment. Journal of Sleep Research. 2009;18(3):321–8.

134. Giannaki CD, Hadjigeorgiou GM, Karatzaferi C, Maridaki MD, Koutedakis Y, Founta P, et al. A single-blind randomized controlled trial to evaluate the effect of 6 months of progressive aerobic exercise training in patients with uraemic restless legs syndrome. Nephrology Dialysis Transplantation. 2013;28(11):2834–40.

135. Giannaki CD, Sakkas GK, Karatzaferi C, Hadjigeorgiou GM, Lavdas E, Kyriakides T, et al. Effect of exercise training and dopamine agonists in patients with uremic restless legs syndrome: a six-month randomized, partially double-blind, placebo-controlled comparative study. BMC Nephrology. 2013;14:194.

136. Gieselmann A, Pietrowsky R. The effects of brief chat-based and face-to-face psychotherapy for insomnia: a randomized waiting list controlled trial. Vol. 61, Sleep Medicine. 2019. p. 63–72.

137. Godzik C, Crawford S, Ryan E. Feasibility of an online cognitive behavioral therapy program to improve insomnia, mood, and quality of life in bereaved adults ages 55 and older. Vol. 42, Geriatric Nursing. 2020. p. 99–106.

138. Goforth HW, Preud'homme XA, Krystal AD. A randomized, double-blind, placebo-controlled trial of eszopiclone for the treatment of insomnia in patients with chronic low back pain. Sleep. 2014;37(6):1053–60.

139. Gogenur I, Kucukakin B, Bisgaard T, Kristiansen V, Hjortso NC, Skene DJ, et al. The effect of melatonin on sleep quality after laparoscopic cholecystectomy: a randomized, placebo-controlled trial. Anesthesia & Analgesia. 2009;108(4):1152–6.

140. Grierson A, Hobbs M, Mason E. Self-guided online cognitive behavioural therapy for insomnia: A naturalistic evaluation in patients with potential psychiatric comorbidities. Vol. 266, Journal of Affective Disorders. 2020. p. 305–10.

141. Gross CR, Kreitzer MJ, Reilly-Spong M, Wall M, Winbush NY, Patterson R, et al. Mindfulness-based stress reduction versus pharmacotherapy for chronic primary insomnia: a randomized controlled clinical trial. Explore: The Journal of Science & Healing. 2011;7(2):76–87.

142. Gross PK, Nourse R, Wasser TE. Ramelteon for insomnia symptoms in a community sample of adults with generalized anxiety disorder: an open label study. Journal of Clinical Sleep Medicine. 2009;5(1):28–33.

143. Guan P, Sun C, Chen Z, Chen J, Ran R. Long-term hydroxychloroquine therapy improves the quality of sleep in patients with primary Sjogren's syndrome: a real-world study. Vol. 9, Annals of Palliative Medicine. 2020. p. 2203–10.

144. Hagatun S, Vedaa O, Harvey AG, Nordgreen T, Smith ORF, Pallesen S, et al. Internet-delivered cognitive-behavioral therapy for insomnia and comorbid symptoms. Internet Interventions. 2018 Jun;12:11–5.

145. Halpern J, Cohen M, Kennedy G, Reece J, Cahan C, Baharav A. Yoga for improving sleep

quality and quality of life for older adults. Alternative Therapies in Health & Medicine. 2014;20(3):37–46.

146. Hansen MV, Andersen LT, Madsen MT, Hageman I, Rasmussen LS, Bokmand S, et al. Effect of melatonin on depressive symptoms and anxiety in patients undergoing breast cancer surgery: a randomized, double-blind, placebo-controlled trial. Breast cancer research and treatment. 2014;145(3):683-695.

147. Hansen MV, Madsen MT, Andersen LT, Hageman I, Rasmussen LS, Bokmand S, et al. Effect of Melatonin on Cognitive Function and Sleep in relation to Breast Cancer Surgery: A Randomized, Double-Blind, Placebo-Controlled Trial. International Journal of Breast Cancer. 2014;2014:416531.

148. Hansson Y, Carlsson C, Olsson E. Intramuscular and periosteal acupuncture for anxiety and sleep quality in patients with chronic musculoskeletal pain--an evaluator blind, controlled study. Acupuncture in Medicine. 2007;25(4):148–57.

149. Hariprasad VR, Sivakumar PT, Koparde V, Varambally S, Thirthalli J, Varghese M, et al. Effects of yoga intervention on sleep and quality-of-life in elderly: A randomized controlled trial. Indian Journal of Psychiatry. 2013 Jul;55(7):S364–8.

150. Harorani M, Davodabady F, Masmouei B, Barati N. The effect of progressive muscle relaxation on anxiety and sleep quality in burn patients: A randomized clinical trial. Vol. 46, Burns. 2020. p. 1107–13.

151. Harris KM, Schiele SE, Emery CF. Pilot randomized trial of brief behavioral treatment for insomnia in patients with heart failure. Vol. 48, Heart & Lung. 2019. p. 373–80.

152. Hartescu I, Morgan K, Stevinson CD. Increased physical activity improves sleep and mood outcomes in sedentary people with insomnia: a randomized controlled trial. Sleep.

2014;37:A174-A175.

153. Harvey AG, Dong L, Belanger L, Morin CM. Mediators and treatment matching in behavior therapy, cognitive therapy and cognitive behavior therapy for chronic insomnia. Journal of Consulting & Clinical Psychology. 2017;85(10):975–87.

154. Herberg KW, Laux G, Fischer W. Analysis of the effects of a 14 days treatment with zopiclone 7.5mg/d on performance capability, actual well-being, and quality of sleep of patients with primary insomnia. [German]. Psychopharmakotherapie. 2002;9(1):25–34.

155. Herring MP, Kline CE, O'Connor PJ. Effects of exercise on sleep among young women with Generalized Anxiety Disorder. Mental Health and Physical Activity. 2015 Oct;9:59–66.

156. Herring WJ, Connor KM, Snyder E, Snavely DB, Morin CM, Lines C, et al. Effects of suvorexant on the Insomnia Severity Index in patients with insomnia: analysis of pooled phase 3 data. Sleep Medicine [Internet]. 2018; Available from: http://www.elsevier.com/inca/publications/store/6/2/0/2/8/2http://ovidsp.ovid.com/ovidweb.cgi?T=JS&CSC=Y&NEWS=N&PAGE=fulltext&D=emexb&AN=2001347789https://pdf.sciencedirectassets.com/272127/1-s2.0-S1389945719X00039/1-s2.0-S138994571830248X/main.pdf?X-Amz-Security-Token=AgoJb3JpZ2luX2VjEEgaCXVzLWVhc3QtMSJHMEUCIQDLEJk6SZ%2BAnXs03hr5x9hCAJH%2FthfbgqGSDR1f6UrQiAIgRZRNjXgqWXndYr%2Btl%2F5rTHxMaLZSfrlEtSkyjLVnamQq4wMIof%2F%2F%2F%2F%2F%2F%2F%2F%2F%2F%2FARACGgwwNTkwMDM1NDY4NjUiDPLxkDHge7JZ2PZ2Giq3A6Ke%2Bh50GbVGA7FWas3%2BHrWisH91MW3lA9qQXEKWcrzFm7Ug3o4DVyMTpuLhPP0mdy0tjPvu%2FKcT%2Blm0YABGls9WuzjcHTRMrmId8pgMeRbDkJnao%2FVhZDljc9skIQ8G7tFePnrfz2di3wBKOT8xArsPAgf5NPJIZDGguAZORIdzpD38DpMknBw

LOF4uptdsLesf8U7o46A9l1%2F%2Bo%2BYewGLyekFNmmE5rKmdGGWAxa0O3tX
M75DrQ6scwZRjw5Pk1bkfTUefaxI1IvlHvzRaY5bibA0INVQpt26MTndpDAM5OUsony
th2dDT0DwHZTpcL5XKyKnTyvSQ2DTjyyV%2BPlKDdmuTOGkpRo%2FkVhJ52hx5d
x%2FvFtmKORKnBYCLWh5vZ5Msed8G6g5z6xXKWyZMhT7MoCHRJ8zPpDD7IK%
2BkVeN5bjnGJ9leKrJ7W2lmOonaKbAnyGknz7ro44RgP1LuUT0%2FQrK%2F0zKowB
yu1B20PVmgwACe2oILLQQdVIOKFiJx6ustgD9OjgBM12SppSEuVQyqSH%2Feo%2F
6OmO3Ij7p%2BTd%2Bcnv4JBXaahQ36CWlumCbLaD80S1n%2BulAw0cyb6QU6tAET
c09YgadyKhFbMYvB2HmV53om2%2F26OY1k086DiPpBIdNMbscq%2FYC3ein4Y1dF
6QAWC5y4ZRSJvhAAHXxA0NyLugIh0UE41H0c9QSc23A%2F2hzaICuK3NCYDkg0r
I1HJhN6T5WRHAESsCBSRRI5N99dyl26WLdakzRqfSsFJFJXtzrwe%2F2WhJc8pXwv1
cBRRT6iKZb4%2BEJRDfXClUqHNJZY%2FSXhcvAJcNVj7jxHXcEbYowu1Os%3D&
X-Amz-Algorithm=AWS4-HMAC-SHA256&X-Amz-Date=20190711T083753Z&X-Am
z-SignedHeaders=host&X-Amz-Expires=300&X-Amz-Credential=ASIAQ3PHCVTYUR
M3QR4E%2F20190711%2Fus-east-1%2Fs3%2Faws4_request&X-Amz-Signature=0a767
fdaefe8d1dd7d880c9e1b7fa682e1b307e7a33b4f4e694d3f923b510f04&hash=c885955d85
14ff3a21cca4f8c63a5bd0cda2d69c8d253fe340f11d5d90c395ba&host=68042c943591013a
c2b2430a89b270f6af2c76d8dfd086a07176afe7c76c2c61&pii=S138994571830248X&tid=
spdf-a166340f-88ca-4f7c-95d6-e665db9d4528&sid=804bd5d683ee264abc9ab43161c054
3c8314gxrqb&type=client

https://pdf.sciencedirectassets.com/272127/1-s2.0-S1389945719X00039/1-s2.0-S1389945
71830248X/main.pdf?X-Amz-Security-Token=AgoJb3JpZ2luX2VjEPf%2F%2F%2F%2F
%2F%2F%2F%2F%2F%2FwEaCXVzLWVhc3QtMSJHMEUCIDnKvMGejVhSkbmixLl
x1W0g5jriMAkv%2B%2B1CzvkAyxp9AiEA%2Ba%2Fx%2BTD0TDlDCyKeL2jPofC7

OcLWdOIeSVhSGDuzFIYq4wMI8P%2F%2F%2F%2F%2F%2F%2F%2F%2F%2FARA
CGgwwNTkwMDM1NDY4NjUiDPPhbxG6ssM3iMS99iq3A0rLCcl0pdCUEwra1aFhEc4
TjT%2BigsroAN6orbR4IC9wT4PmE%2BoBo91gGqZchB39%2FGAsByGI81SjYTVxT0
vZfHs%2BX%2FvoVyWhbddVF0EnRUjUORD4STg48b01xE7hrLFjKir7Q3q%2Fdzc71
ZScfrCUaDn6b%2BPI0prRZRuObCi%2BP7z2bETvH0MxNvbtAdshsf6RpsEF8pd52m2h
ffdEl2V0kGJaHCwn8lOTl%2BEeijXOfEi3nwVpP8OJt5KpQye%2F0w1wsDh8s6261bq
wuEeZBekknShgOhQEIQoYOgQJVTAHGszFT%2F8p%2BG9UbMDi3kO9XLpwQRw
UoZ94X6MJjypfB40agGE7TIdriBL%2Fo7yKMSHJZ53G6XNzX6%2BWPdJ7aFXv4YS
Zxli7CQ52cClTAibRyjbPc%2B3nFGO9%2BtupgpSFZVSE2KROuPsTjlOvRIHX25pFf
MTg6VUStuorXxe9EZaqMQgRH6EF1UUZ2Sa%2FEB9db7xa7oAdw0Q3LpfKeh0bbhV
lMXpOKQp6XsSjc0GmUVHTQMXZDw9aSxH27xvdRf2hH7F5uwtsKgM%2BoNHzyr0
ss2vZIuG8DA4RKZfZufkw1LK87QU6tAFLxpuuS8XJJ5WiJ8HEBPHlSYcIYa4UtKQv6
Yl3ioEZQcvLpSpwxmSzEhqqAf%2FNTzP%2BtgSEjPQGvB8DfKg4q%2FKJzgAZrFlrp
NJBoGGDSOFN%2FkIUzi6TqgiAuMCVMsBcsoDNC5hMmZW1PpDO5MCt6zg%2F9s
o%2BKodTCCEaZ3xaSN1GqipqX3JYWTeq%2Be3ZY5rFq%2BM%2FL1PnZCu7YDG
9%2BJI2o9Gt89ytQMZiKCSNVyTS8SJ8MzYU7xs%3D&X-Amz-Algorithm=AWS4-H
MAC-SHA256&X-Amz-Date=20191022T160217Z&X-Amz-SignedHeaders=host&X-A
mz-Expires=300&X-Amz-Credential=ASIAQ3PHCVTYWZZUWJMR%2F20191022%2
Fus-east-1%2Fs3%2Faws4_request&X-Amz-Signature=6c89a7c3291afd0094ace2bc5761
17ad9ff144cfbabe4cc05a67b4951944dbd3&hash=2347f132289ec1a6753031c4f978b1c01
bf2d3b686b13b8d9ee64261d3c955eb&host=68042c943591013ac2b2430a89b270f6af2c7
6d8dfd086a07176afe7c76c2c61&pii=S138994571830248X&tid=spdf-a88f031b-501c-4b1
4-974f-9a0f130bb47c&sid=48de36e7314b8641ce6954320d917f865a8dgxrqb&type=client

157. Hicks JA, Argyropoulos SV, Rich AS, Nash JR, Bell CJ, Edwards C, et al. Randomised controlled study of sleep after nefazodone or paroxetine treatment in out-patients with depression. British Journal of Psychiatry. 2002;180:528–35.

158. Hirshkowitz M, Black J. Effect of adjunctive modafinil on wakefulness and quality of life in patients with excessive sleepiness-associated obstructive sleep apnoea/hypopnoea syndrome: a 12-month, open-label extension study. CNS Drugs. 2007;21(5):407–16.

159. Hirshkowitz M, Black JE, Wesnes K, Niebler G, Arora S, Roth T. Adjunct armodafinil improves wakefulness and memory in obstructive sleep apnea/hypopnea syndrome. Respiratory Medicine. 2007;101(3):616–27.

160. Hoch CC, Reynolds CF 3rd, Buysse DJ, Monk TH, Nowell P, Begley AE, et al. Protecting sleep quality in later life: a pilot study of bed restriction and sleep hygiene. Journals of Gerontology Series B-Psychological Sciences & Social Sciences. 2001;56(1):P52-59.

161. Hogl B, Garcia-Borreguero D, Trenkwalder C, Ferini-Strambi L, Hening W, Poewe W, et al. Efficacy and augmentation during 6 months of double-blind pramipexole for restless legs syndrome. Sleep Medicine. 2011;12(4):351–60.

162. Hogl B, Oertel WH, Stiasny-Kolster K, Geisler P, Benes H, Garcia-Borreguero D, et al. Treatment of moderate to severe restless legs syndrome: 2-year safety and efficacy of rotigotine transdermal patch. BMC Neurology. 2010;10:86.

163. Horsch CH, Lancee J, Griffioen-Both F, Spruit S, Fitrianie S, Neerincx MA, et al. Mobile phone-delivered cognitive behavioral therapy for insomnia: A randomized waitlist controlled trial. Journal of Medical Internet Research. 2017 Apr;19(4):60–80.

164. Hou Y-M, Hu P-C, Liang Y-P, Mo Z-Y. Effects of cognitive behavior therapy on insomnia of maintained hemodialysis patients: A randomly controlled study. Chinese

Mental Health Journal. 2009 Aug;23(8):538–42.

165. Hsu HM, Chou KR, Lin KC, Chen KY, Su SF, Chung MH. Effects of cognitive behavioral therapy in patients with depressive disorder and comorbid insomnia: A propensity score-matched outcome study. Behaviour Research & Therapy. 2015;73:143–50.

166. Hu LL, Zhang X, Liu WJ, Li M, Zhang YH. Suan zao ren tang in combination with zhi zi chi tang as a treatment protocol for insomniacs with anxiety: a randomized parallel-controlled trial. Evidence-Based Complementary & Alternative Medicine: eCAM. 2015;2015:913252.

167. Hu ST, Yu CC, Liu CY, Tsao LI. The effects of integrated nursing education on quality of life and health-related outcomes among obstructive sleep apnea patients receiving continuous positive airway pressure therapy. Sleep & Breathing. 2017;21(4):845–52.

168. Huang GG, Chen Q, Li L. Comparison between the effect of behavioral and drug therapy on the treatment of insomnia in patients with schizophrenia in rehabilitation period. [Chinese]. Chinese Journal of Clinical Rehabilitation. 2004 Mar;8(9):1628–9.

169. Huang QL, Yue FG, Jiang CG, Zhang T, Lei L, Wang YJ, et al. The treatment effect of group cognitive behavioral therapy for insomnia. [Chinese]. Medical Journal of Chinese People's Liberation Army. 2018;43(3):224–8.

170. Huang W, Johnson T, Kutner N, Halpin S, Weiss P, Griffiths P, et al. Acupuncture for treatment of persistent disturbed sleep: a randomized clinical trial in veterans with mild traumatic brain injury and post-traumatic stress disorder. Annals of physical and rehabilitation medicine [Internet]. 2018;(no pagination). Available from: https://www.cochranelibrary.com/central/doi/10.1002/central/CN-01610916/fullhttps://pdf

.sciencedirectassets.com/277754/1-s2.0-S1877065718X00050/1-s2.0-S187706571830265
3/main.pdf?X-Amz-Security-Token=AgoJb3JpZ2luX2VjEEoaCXVzLWVhc3QtMSJHM
EUCIA5WBpjDPpmUZFLqJzFDJ3Tn6XEs96NHK5qxJwrwlN5wAiEAkkalDybVN93xq
aJs5io%2FgetKBYW%2FTiav2AZmaGke2C8q4wMIo%2F%2F%2F%2F%2F%2F%2F%
2F%2F%2F%2FARACGgwwNTkwMDM1NDY4NjUiDATWRwewWblPtNmR%2Byq3
A7dU60ZeWmb3U1cVpn%2BPhY%2FTYl0Lt7mUk774kb4zqhRyujpm7GTM47uJsVL
KCI93pl3Utaz3a6VjK2JB4AbPUuL749ceYMWg3cq35Fd4oaCYbcY5QJRIEU5d6ShU5q
JdKwqACuLz5dQWO9YnUVIMoEJINlsMxTSpkm06BpMgJcq07wuMTT%2FZM5D61
FvjdwS7MK0GmgH%2FTap9wxBuIzV4XbHtBz7qcwmiceprZa7bc9auARKIhMgalmjuR
gcRAg%2F1jiFaagg2GVsaok3OLWF8LyQCD%2By2W%2FyrEA%2BZJ9Ls4msPbud8
VEbdmbLcTlvv48lvQ9uqGPR5mYyxtcWYypUXIOmaK8TaUa7OA%2FgasbZ%2BMcf
yR%2FzvtZp7UT1k6DlbpF%2BfsGTDsw3sVfEWuX27W8Hjzu08vkLillz70IsZI9ow3VT
xBq9NwJibw%2FF7Bpeym4daegheCKtmCLVOyTfl3S3U9BVtb02EG%2F783qsfPYFzS
B8l9q9xzm5%2FwaYY6Bry%2Fw1ExgtX%2FkNoxjqaSrYKT3PN%2BFbfRTYTE6fnm
aTbSMCMNHh3iNQdFTEH%2F1SFICXBYWeOnR0Fq1SM6dcwr4Oc6QU6tAECtKo7
crdtGnJqy%2FHf%2FiJMJE7IIQwkkarUzC4ah0fD5w90CYWZcv5DeqcuGycK64H7ZR
VaZVmRQ2zOXj%2Bi2ixxSdPzGAeh01jU6m%2F4pWSKo8s9GD69M7jMmRss3xxdsr
bWzMqUBBZiCnFvwNNGb%2FLssPfdFBG8DyqUo28JzVz0m6q86OARl5ekW0m4V4
6%2FS74q0WTsfRw6PPI5T7LBtPsYGuKT2ryQSI9NaZQ1dh7wkfXbd2U%3D&X-Amz
-Algorithm=AWS4-HMAC-SHA256&X-Amz-Date=20190711T095646Z&X-Amz-Signe
dHeaders=host&X-Amz-Expires=300&X-Amz-Credential=ASIAQ3PHCVTYYG425CE
K%2F20190711%2Fus-east-1%2Fs3%2Faws4_request&X-Amz-Signature=cc575132a09
59f4a50367429d57784b37f7ab2455aba3534d7440217ca732ae6&hash=7c2d7c8b77b2af4

5e8894075d8458e0f0fea06e145a0626afb4ab758a4269957&host=68042c943591013ac2b2430a89b270f6af2c76d8dfd086a07176afe7c76c2c61&pii=S1877065718302653&tid=spdf-0272b62e-a3f1-44f2-9b17-6021a536936e&sid=804bd5d683ee264abc9ab43161c0543c8314gxrqb&type=client

https://pdf.sciencedirectassets.com/277754/1-s2.0-S1877065718X00050/1-s2.0-S1877065718302653/main.pdf?X-Amz-Security-Token=AgoJb3JpZ2luX2VjEPf%2F%2F%2F%2F%2F%2F%2F%2F%2F%2FwEaCXVzLWVhc3QtMSJHMEUCIDnKvMGejVhSkbmixLlx1W0g5jriMAkv%2B%2B1CzvkAyxp9AiEA%2Ba%2Fx%2BTD0TDlDCyKeL2jPofC7OcLWdOIeSVhSGDuzFIYq4wMI8P%2F%2F%2F%2F%2F%2F%2F%2F%2F%2F%2FARACGgwwNTkwMDM1NDY4NjUiDPPhbxG6ssM3iMS99iq3A0rLCcl0pdCUEwra1aFhEc4TjT%2BigsroAN6orbR4IC9wT4PmE%2BoBo91gGqZchB39%2FGAsByGI81SjYTVxT0vZfHs%2BX%2FvoVyWhbddVF0EnRUjUORD4STg48b01xE7hrLFjKir7Q3q%2Fdzc71ZScfrCUaDn6b%2BPI0prRZRuObCi%2BP7z2bETvH0MxNvbtAdshsf6RpsEF8pd52m2hffdEl2V0kGJaHCwn8lOTl%2BEeijXOfEi3nwVpP8OJt5KpQye%2F0w1wsDh8s6261bqwuEeZBekknShgOhQEIQoYOgQJVTAHGszFT%2F8p%2BG9UbMDi3kO9XLpwQRwUoZ94X6MJjypfB40agGE7TIdriBL%2Fo7yKMSHJZ53G6XNzX6%2BWPdJ7aFXv4YSZxli7CQ52cClTAibRyjbPc%2B3nFGO9%2BtupgpSFZVSE2KROuPsTjlOvRIHX25pFfMTg6VUStuorXxe9EZaqMQgRH6EF1UUZ2Sa%2FEB9db7xa7oAdw0Q3LpfKeh0bbhVlMXpOKQp6XsSjc0GmUVHTQMXZDw9aSxH27xvdRf2hH7F5uwtsKgM%2BoNHzyr0ss2vZIuG8DA4RKZfZufkw1LK87QU6tAFLxpuuS8XJJ5WiJ8HEBPHlSYcIYa4UtKQv6Yl3ioEZQcvLpSpwxmSzEhqqAf%2FNTzP%2BtgSEjPQGvB8DfKg4q%2FKJzgAZrFlrpNJBoGGDSOFN%2FkIUzi6TqgiAuMCVMsBcsoDNC5hMmZW1PpDO5MCt6zg%2F9so%2BKodTCCEaZ3xaSN1GqipqX3JYWTeq%2Be3ZY5rFq%2BM%2FL1PnZCu7YDG

9%2BJI2o9Gt89ytQMZiKCSNVyTS8SJ8MzYU7xs%3D&X-Amz-Algorithm=AWS4-HMAC-SHA256&X-Amz-Date=20191022T160243Z&X-Amz-SignedHeaders=host&X-Amz-Expires=300&X-Amz-Credential=ASIAQ3PHCVTYWZZUWJMR%2F20191022%2Fus-east-1%2Fs3%2Faws4_request&X-Amz-Signature=d68801f81e0fb1703a98eefc09b6456ff88eaf016f51dab3430bbc431e9827fc&hash=daaebc89b912e45eb69785367b27f7bcdcec5468caf20149c8b323f91ffebdcc&host=68042c943591013ac2b2430a89b270f6af2c76d8dfd086a07176afe7c76c2c61&pii=S1877065718302653&tid=spdf-5e362337-98ec-465f-bad4-d90d93294ad8&sid=48de36e7314b8641ce6954320d917f865a8dgxrqb&type=client

171. Huang Y, Zheng Y. Sleep disorder of schizophrenia treated with shallow needling: a randomized controlled trial. Zhongguo Zhenjiu. 2015;35(9):869–73.

172. Hwang DK, Nam M, Lee YJG. The effect of cognitive behavioral therapy for insomnia in schizophrenia patients with sleep Disturbance: A non-randomized, assessor-blind trial. Psychiatry Research. 2019 Apr;274:182–8.

173. Innes KE, Selfe TK, Khalsa DS, Kandati S. Effects of Meditation versus Music Listening on Perceived Stress, Mood, Sleep, and Quality of Life in Adults with Early Memory Loss: A Pilot Randomized Controlled Trial. Journal of Alzheimer’s Disease. 2016;52(4):1277–98.

174. Irwin MR, Olmstead R, Carrillo C, Sadeghi N, Nicassio P, Ganz PA, et al. Tai Chi Chih Compared With Cognitive Behavioral Therapy for the Treatment of Insomnia in Survivors of Breast Cancer: A Randomized, Partially Blinded, Noninferiority Trial. Journal of Clinical Oncology. 2017;35(23):2656–65.

175. Jacobs BP, Bent S, Tice JA, Blackwell T, Cummings SR. An internet-based randomized, placebo-controlled trial of kava and valerian for anxiety and insomnia. Medicine.

2005;84(4):197–207.

176. Jacobs GD, Pace-Schott EF, Stickgold R, Otto MW. Cognitive behavior therapy and pharmacotherapy for insomnia: a randomized controlled trial and direct comparison. Archives of Internal Medicine. 2004;164(17):1888–96.

177. James M. Efficacy of individualized homeopathic treatment of insomnia: Double-blind, randomized, placebo-controlled clinical trial. Complementary Therapies in Medicine. 2019 Apr;43:53–9.

178. Janku K, Smotek M, Farkova E, Koprivova J. Block the light and sleep well: Evening blue light filtration as a part of cognitive behavioral therapy for insomnia. Vol. 37, Chronobiology International. 2020. p. 248–59.

179. Jansson M, Linton SJ. Cognitive-behavioral group therapy as an early intervention for insomnia: a randomized controlled trial. Journal of occupational rehabilitation. 2005;15(2):177-190.

180. Jansson-Frojmark M, Jacobson K. Cognitive behavioural therapy for insomnia for patients with co-morbid generalized anxiety disorder: an open trial on clinical outcomes and putative mechanisms. Behavioural & Cognitive Psychotherapy. 2021. p. 1–16.

181. Jansson-Frojmark M, Lind M, Sunnhed R. Don’t worry, be constructive: a randomized controlled feasibility study comparing behaviour therapy singly and combined with constructive worry for insomnia. British Journal of Clinical Psychology. 2012;51(2):142–57.

182. Jarnefelt H, Harma M, Sallinen M, Virkkala J, Paajanen T, Martimo KP, et al. Cognitive behavioural therapy interventions for insomnia among shift workers: RCT in an occupational health setting. Vol. 93, International Archives of Occupational &

Environmental Health. 2020. p. 535–50.

183. Jespersen KV, Otto M, Kringelbach M, Van Someren E, Vuust P. A randomized controlled trial of bedtime music for insomnia disorder. Journal of Sleep Research [Internet]. 2019;(no pagination)(e12817). Available from: http://onlinelibrary.wiley.com/journal/10.1111/(ISSN)1365-2869http://ovidsp.ovid.com/ovidweb.cgi?T=JS&CSC=Y&NEWS=N&PAGE=fulltext&D=emexb&AN=626129784https://onlinelibrary.wiley.com/doi/abs/10.1111/jsr.12817https://onlinelibrary.wiley.com/doi/pdf/10.1111/jsr.12817 https://onlinelibrary.wiley.com/doi/full/10.1111/jsr.12817

184. Ji XW, Ng SM, Chan CLW, Chan JSM, Chan CHY, Chung KF. Integrative body-mind-spirit intervention for concurrent sleep and mood disturbances: sleep-specific daytime functioning mediates sleep and mood improvements. Journal of Sleep Research. 2018 Feb;27(1):56–63.

185. Jokar A, Kargosha A, Akarzadeh M, Asadi N, Setoudeh Z. Comparing the influence of relaxation training and consumption of valerian on insomnia of menopause women: A randomized clinical trial. African Journal of Traditional, Complementary and Alternative Medicines. 2016;13(1):40–4.

186. Kanady JC, Talbot LS, Maguen S, Straus LD, Richards A, Ruoff L, et al. Cognitive Behavioral Therapy for Insomnia Reduces Fear of Sleep in Individuals With Posttraumatic Stress Disorder. Journal of Clinical Sleep Medicine. 2018 Jul 15;14(7):1193–203.

187. Kasper S, Anghelescu I, Dienel A. Efficacy of orally administered Silexan in patients with anxiety-related restlessness and disturbed sleep--A randomized, placebo-controlled trial. European Neuropsychopharmacology. 2015;25(11):1960–7.

188. Khastar H, Mirrezaie SM, Chashmi NA, Jahanfar S. Sleep Improvement Effect on Sexual

Life Quality Among Rotating Female Shift Workers: A Randomized Controlled Trial. Vol. 17, Journal of Sexual Medicine. 2020. p. 1467–75.

189. Kim SJ, Lee YJ, Cho SJ. Effect of quetiapine XR on depressive symptoms and sleep quality compared with lithium in patients with bipolar depression. Journal of Affective Disorders. 2014;157:33–40.

190. Konsta A, Dikeos D, Bonakis A, Economou N, Chrousos G, Darviri C. Stress management techniques in primary insomnia: a randomized controlled trial. Sleep medicine. 2013;14:e173-.

191. Krieger T, Urech A, Duss SB, Blattler L, Schmitt W, Gast H, et al. A randomized controlled trial comparing guided internet-based multi-component treatment and internet-based guided sleep restriction treatment to care as usual in insomnia. Vol. 62, Sleep Medicine. 2019. p. 43–52.

192. Krystal AD, Erman M, Zammit GK, Soubrane C, Roth T, Group ZS. Long-term efficacy and safety of zolpidem extended-release 12.5 mg, administered 3 to 7 nights per week for 24 weeks, in patients with chronic primary insomnia: a 6-month, randomized, double-blind, placebo-controlled, parallel-group, multicenter study. Sleep. 2008;31(1):79–90.

193. Krystal AD, Walsh JK, Laska E, Caron J, Amato DA, Wessel TC, et al. Sustained efficacy of eszopiclone over 6 months of nightly treatment: results of a randomized, double-blind, placebo-controlled study in adults with chronic insomnia. Sleep. 2003;26(7):793–9.

194. Krystal A, Fava M, Rubens R, Wessel T, Caron J, Wilson P, et al. Evaluation of eszopiclone discontinuation after cotherapy with fluoxetine for insomnia with coexisting depression. Journal of Clinical Sleep Medicine. 2007;3(1):48–55.

195. Kunze AE, Lancee J, Morina N, Kindt M, Arntz A. Mediators of Change in Imagery Rescripting and Imaginal Exposure for Nightmares: Evidence From a Randomized Wait-List Controlled Trial. Vol. 50, Behavior Therapy. 2019. p. 978–93.

196. Kurdi MS, Muthukalai SP. The Efficacy of Oral Melatonin in Improving Sleep in Cancer Patients with Insomnia: A Randomized Double-Blind Placebo-Controlled Study. Indian Journal of Palliative Care. 2016;22(3):295–300.

197. Lai HL, Good M. Music improves sleep quality in older adults. Journal of Advanced Nursing. 2005;49(3):234–44.

198. Lai H-L. The effects of music therapy on sleep quality in elderly people. Dissertation Abstracts International: Section B: The Sciences and Engineering. 2002 May;62(11-B):5034.

199. Lam B, Sam K, Mok WY, Cheung MT, Fong DY, Lam JC, et al. Randomised study of three non-surgical treatments in mild to moderate obstructive sleep apnoea. Thorax. 2007;62(4):354–9.

200. Lam TH, Chung KF, Lee CT, Yeung WF, Yu BY. Hypnotherapy for insomnia: A randomized controlled trial comparing generic and disease-specific suggestions. Complementary Therapies in Medicine. 2018;41:231–9.

201. Lami MJ, Martinez MP, Miro E, Sanchez AI, Prados G, Caliz R, et al. Efficacy of Combined Cognitive-Behavioral Therapy for Insomnia and Pain in Patients with Fibromyalgia: A Randomized Controlled Trial. Cognitive Therapy and Research. 2018 Feb;42(1):63–79.

202. Lancee J, Effting M, Kunze AE. Telephone-guided imagery rehearsal therapy for nightmares: Efficacy and mediator of change [Internet]. Journal of Sleep Research. 2020.

Available from:
http://ovidsp.ovid.com/ovidweb.cgi?T=JS&CSC=Y&NEWS=N&PAGE=fulltext&D=emexb&AN=2005415927
http://resolver.shef.ac.uk/?http://find.shef.ac.uk/openurl/44SFD/44SFD_services_page?sid=OVID:embase&id=pmid:32567103&id=doi:10.1111%2Fjsr.13123&issn=0962-1105&isbn=&volume=&issue=&spage=e13123&pages=&date=2020&title=Journal+of+Sleep+Research&atitle=Telephone-guided+imagery+rehearsal+therapy+for+nightmares%3A+Efficacy+and+mediator+of+change&aulast=Lancee&pid=%3Cauthor%3ELancee+J.%3BEffting+M.%3BKunze+A.E.%3C%2Fauthor%3E%3CAN%3E2005415927%3C%2FAN%3E%3CDT%3EArticle%3C%2FDT%3E

203. Lancee J, Eisma MC, van Straten A, Kamphuis JH. Sleep-Related Safety Behaviors and Dysfunctional Beliefs Mediate the Efficacy of Online CBT for Insomnia: A Randomized Controlled Trial. Cognitive Behaviour Therapy. 2015;44(5):406–22.

204. Lancee J, Sorbi MJ, Eisma MC, van Straten A, van den Bout J. The effect of support on internet-delivered treatment for insomnia: does baseline depression severity matter? Behavior Therapy. 2014;45(4):507–16.

205. Lancee J, Yasiney SL, Brendel RS, Boffo M, Clarke PJF, Salemink E. Attentional bias modification training for insomnia: A double-blind placebo controlled randomized trial. PLoS ONE [Electronic Resource]. 2017;12(4):e0174531.

206. Laurel Franklin C, Walton JL, Raines AM, Chambliss JL, Corrigan SA, Cuccurullo LJ, et al. Pilot study comparing telephone to in-person delivery of cognitive-behavioural therapy for trauma-related insomnia for rural veterans. Journal of Telemedicine & Telecare. 2018;24(9):629–35.

207. Lazaridou A, Koulouris A, Devine JK, Haack M, Jamison RN, Edwards RR, et al. Impact of daily yoga-based exercise on pain, catastrophizing, and sleep amongst individuals with fibromyalgia. Vol. 12, Journal of pain research. 2019. p. 2915–23.

208. Lazowski LK, Townsend B, Hawken ER, Jokic R, du Toit R, Milev R. Sleep architecture and cognitive changes in olanzapine-treated patients with depression: a double blind randomized placebo controlled trial. BMC Psychiatry. 2014;14:202.

209. Le Bon O, Murphy JR, Staner L, Hoffmann G, Kormoss N, Kentos M, et al. Double-blind, placebo-controlled study of the efficacy of trazodone in alcohol post-withdrawal syndrome: polysomnographic and clinical evaluations. Journal of Clinical Psychopharmacology. 2003;23(4):377–83.

210. Lee J, Jung H-Y, Lee SI, Choi JH, Kim S-G. Effects of passiflora incarnata linnaeus on polysomnographic sleep parameters in subjects with insomnia disorder: A double-blind randomized placebo-controlled study. Vol. 35, International Clinical Psychopharmacology. 2020. p. 29–35.

211. Leger D, Quera-Salva MA, Philip P. Health-related quality of life in patients with insomnia treated with zopiclone. Pharmacoeconomics. 1996;10 Suppl 1:39–44.

212. Lehrl S. Clinical efficacy of kava extract WS 1490 in sleep disturbances associated with anxiety disorders. Results of a multicenter, randomized, placebo-controlled, double-blind clinical trial. Journal of Affective Disorders. 2004;78(2):101–10.

213. Li C, Li DL, Zheng H, Li ZW, Du HR. Influence of Estazolam on the sleep quality and daytime function of patients with insomnia. [Chinese]. Journal of Clinical Rehabilitative Tissue Engineering Research. 2007 Dec 30;11(52):10483–5.

214. Li F, Fisher KJ, Harmer P, Irbe D, Tearse RG, Weimer C. Tai chi and self-rated quality of

sleep and daytime sleepiness in older adults: a randomized controlled trial. Journal of the American Geriatrics Society. 2004;52(6):892–900.

215. Lichstein KL, Wilson NM, Johnson CT. Psychological treatment of secondary insomnia. Psychology & Aging. 2000;15(2):232–40.

216. Lien M, Bredeli E, Sivertsen B, Kallestad H, Pallesen S, Smith ORF, et al. Short and long-term effects of unguided internet-based cognitive behavioral therapy for chronic insomnia in morning and evening persons: a post-hoc analysis. Vol. 36, Chronobiology International. 2019. p. 1384–98.

217. Lillehei AS. Effect of lavender aromatherapy via inhalation and sleep hygiene on sleep in college students with self-reported sleep issues. Dissertation Abstracts International: Section B: The Sciences and Engineering. 2015;75(12-B(E)):No Pagination Specified.

218. Liu YH, Chang CF, Hung HM, Chen CH. Outcomes of a walking exercise intervention in postpartum women with disordered sleep. Vol. 25, Journal of Obstetrics & Gynaecology Research. 2021. p. 25.

219. Locklear JC, Svedsater H, Datto C, Endicott J. Effects of once-daily extended release quetiapine fumarate (quetiapine XR) on quality of life and sleep in elderly patients with major depressive disorder. Journal of Affective Disorders. 2013;149(1–3):189–95.

220. Lohr JB, Liu L, Caligiuri MP, Kash TP, May TA, Murphy JD, et al. Modafinil improves antipsychotic-induced parkinsonism but not excessive daytime sleepiness, psychiatric symptoms or cognition in schizophrenia and schizoaffective disorder: a randomized, double-blind, placebo-controlled study. Schizophrenia Research. 2013;150(1):289–96.

221. Londborg PD, Smith WT, Glaudin V, Painter JR. Short-term cotherapy with clonazepam and fluoxetine: anxiety, sleep disturbance and core symptoms of depression. Journal of

Affective Disorders. 2000;61(1–2):73–9.

222. Lorenz N, Heim E, Roetger A, Birrer E, Maercker A. Randomized Controlled Trial to Test the Efficacy of an Unguided Online Intervention with Automated Feedback for the Treatment of Insomnia. Vol. 47, Behavioural and cognitive psychotherapy. 2019. p. 287-302.

223. Lorenz RA, Auerbach S, Nisbet P, Sessanna L, Alanazi N, Lach H, et al. Improving Sleep among Adults with Multiple Sclerosis using Mindfulness plus Sleep Education. Vol. 43, Western Journal of Nursing Research. 2021. p. 273–83.

224. Lovato N, Lack L, Kennaway DJ. Comparing and contrasting therapeutic effects of cognitive-behavior therapy for older adults suffering from insomnia with short and long objective sleep duration. Sleep Medicine. 2016;22:4–12.

225. Lovato N, Lack L, Wright H, Kennaway DJ. Evaluation of a brief treatment program of cognitive behavior therapy for insomnia in older adults. Sleep. 2014;37(1):117–26.

226. Loving RT, Kripke DF, Knickerbocker NC, Grandner MA. Bright green light treatment of depression for older adults [ISRCTN69400161]. BMC Psychiatry [Internet]. 2005 Nov;5 (no pagination)(42). Available from: http://www.biomedcentral.com/1471-244X/5/42http://ovidsp.ovid.com/ovidweb.cgi?T=JS&CSC=Y&NEWS=N&PAGE=fulltext&D=emed9&AN=41662750https://bmcpsychiatry.biomedcentral.com/track/pdf/10.1186/1471-244X-5-42 https://bmcpsychiatry.biomedcentral.com/track/pdf/10.1186/1471-244X-5-42

227. Lu J, Huang L, Wu X, Fu W, Liu Y. Effect of Tai Ji Quan training on self-reported sleep quality in elderly Chinese women with knee osteoarthritis: a randomized controlled trail. Sleep Medicine. 2017;33:70–5.

228. Luik AI, Farias Machado P, Espie CA. Delivering digital cognitive behavioral therapy for insomnia at scale: does using a wearable device to estimate sleep influence therapy? Vol. 1, Npj Digital Medicine. 2018. p. 3.

229. Luik AI, Marsden A, Emsley R, Henry AL, Stott R, Miller CB, et al. Long-term benefits of digital cognitive behavioural therapy for insomnia: Follow-up report from a randomized clinical trial. Vol. 29, Journal of Sleep Research. 2020. p. e13018.

230. Ma R, Tao HY, Xue R. Clinical study on repetitive transcranial magnetic stimulation for the improvement of sleep quality in patients with depression. [Chinese]. Chinese Journal of Contemporary Neurology and Neurosurgery. 2013 Jun;13(6):500–5.

231. Madsen MT, Isbrand A, Andersen UO, Andersen LJ, Taskiran M, Simonsen E, et al. The effect of MElatonin on Depressive symptoms, Anxiety, CIrcadian and Sleep disturbances in patients after acute coronary syndrome (MEDACIS): study protocol for a randomized controlled trial. Trials [Electronic Resource]. 2017;18(1):81.

232. Maguen S, Gloria R, Huggins J, Goldstein LA, Kanady JC, Straus LD, et al. Brief Behavioral Treatment for Insomnia Improves Psychosocial Functioning in Veterans: Results from a Randomized Controlled Trial. Vol. 06, Sleep. 2020. p. 06.

233. Manber R, Bei B, Simpson N, Asarnow L, Rangel E, Sit A, et al. Cognitive Behavioral Therapy for Prenatal Insomnia: A Randomized Controlled Trial. Obstetrics & Gynecology. 2019;133(5):911–9.

234. Manber R, Buysse DJ, Edinger J, Krystal A, Luther JF, Wisniewski SR, et al. Efficacy of Cognitive-Behavioral Therapy for Insomnia Combined With Antidepressant Pharmacotherapy in Patients With Comorbid Depression and Insomnia: A Randomized Controlled Trial. Journal of Clinical Psychiatry. 2016;77(10):e1316–23.

235. Manber R, Edinger JD, Gress JL, San Pedro-Salcedo MG, Kuo TF, Kalista T. Cognitive behavioral therapy for insomnia enhances depression outcome in patients with comorbid major depressive disorder and insomnia. Sleep. 2008;31(4):489–95.

236. Manber R, Rush AJ, Thase ME, Amow B, Klein D, Trivedi MH, et al. The effects of psychotherapy, nefazodone, and their combination on subjective assessment of disturbed sleep in chronic depression. Sleep. 2003;26(2):130–6.

237. Mao JJ, Farrar JT, Bruner D, Zee J, Bowman M, Seluzicki C, et al. Electroacupuncture for fatigue, sleep, and psychological distress in breast cancer patients with aromatase inhibitor-related arthralgia: a randomized trial. Cancer. 2014;120(23):3744–51.

238. Marshall-McKenna R, Morrison A, Stirling L, Hutchison C, Rice AM, Hewitt C, et al. A randomised trial of the cool pad pillow topper versus standard care for sleep disturbance and hot flushes in women on endocrine therapy for breast cancer. Supportive Care in Cancer. 2016;24(4):1821–9.

239. Martinez-Ceron E, Barquiel B, Bezos AM, Casitas R, Galera R, Garcia-Benito C, et al. Effect of Continuous Positive Airway Pressure on Glycemic Control in Patients with Obstructive Sleep Apnea and Type 2 Diabetes. A Randomized Clinical Trial. American Journal of Respiratory & Critical Care Medicine. 2016;194(4):476–85.

240. Martiny K, Refsgaard E, Lund V, Lunde M, Sorensen L, Thougaard B, et al. A 9-week randomized trial comparing a chronotherapeutic intervention (wake and light therapy) to exercise in major depressive disorder patients treated with duloxetine. Journal of Clinical Psychiatry. 2012 Sep;73(9):1234–42.

241. Martiny K, Refsgaard E, Lund V, Lunde M, Thougaard B, Lindberg L, et al. Maintained superiority of chronotherapeutics vs. exercise in a 20-week randomized follow-up trial in

major depression. Acta psychiatrica scandinavica. 2015;131(6):446-457.

242. Mashayekh-Amiri S, Delavar MA, Bakouei F, Faramarzi M, Esmaeilzadeh S. The impact of myo-inositol supplementation on sleep quality in pregnant women: a randomized, double-blind, placebo-controlled study. Journal of Maternal-Fetal & Neonatal Medicine. 2020. p. 1–9.

243. Matthews EE, Berger AM, Schmiege SJ, Cook PF, McCarthy MS, Moore CM, et al. Cognitive behavioral therapy for insomnia outcomes in women after primary breast cancer treatment: a randomized, controlled trial. Oncology Nursing Forum. 2014;41(3):241–53.

244. Mauri MC, Gianetti S, Pugnetti L, Altamura AC. Quazepam versus triazolam in patients with sleep disorders: a double-blind study. International journal of clinical pharmacology research. 1993;13(3):173-177.

245. McCall WV, Benca RM, Rosenquist PB, Youssef NA, McCloud L, Newman JC, et al. Reducing suicidal ideation through insomnia treatment (REST-IT): A randomized clinical trial. Vol. 176, American Journal of Psychiatry. 2019. p. 957–65.

246. McCall WV, Pillai A, Case D, McCloud L, Nolla T, Branch F, et al. A Pilot, Randomized Clinical Trial of Bedtime Doses of Prazosin Versus Placebo in Suicidal Posttraumatic Stress Disorder Patients With Nightmares. Journal of clinical psychopharmacology. 2018;38(6):618-621.

247. McCurry SM, Gibbons LE, Logsdon RG, Vitiello MV, Teri L. Nighttime insomnia treatment and education for Alzheimer’s disease: a randomized, controlled trial. Journal of the American Geriatrics Society. 2005;53(5):793–802.

248. McCurry SM, LaFazia DM, Pike KC, Logsdon RG, Teri L. Development and evaluation of a sleep education program for older adults with dementia living in adult family homes.

American Journal of Geriatric Psychiatry. 2012;20(6):494–504.

249. McMillan A, Bratton DJ, Faria R, Laskawiec-Szkonter M, Griffin S, Davies RJ, et al. Continuous positive airway pressure in older people with obstructive sleep apnoea syndrome (PREDICT): a 12-month, multicentre, randomised trial. The Lancet Respiratory Medicine. 2014;2(10):804–12.

250. McQuade JL, Prinsloo S, Chang DZ, Spelman A, Wei Q, Basen-Engquist K, et al. Qigong/tai chi for sleep and fatigue in prostate cancer patients undergoing radiotherapy: a randomized controlled trial. Psycho-Oncology. 2017;26(11):1936–43.

251. Meurice JC, Ingrand P, Portier F, Arnulf I, Rakotonanahari D, Fournier E, et al. A multicentre trial of education strategies at CPAP induction in the treatment of severe sleep apnoea-hypopnoea syndrome. Sleep Medicine. 2007;8(1):37–42.

252. Mitchell KR. Behavioral treatment of presleep tension and intrusive cognitions in patients with severe predormital insomnia. Journal of Behavioral Medicine. 1979;2(1):57–69.

253. Miyake M, Kirisako T, Kokubo T, Miura Y, Morishita K, Okamura H, et al. Randomised controlled trial of the effects of L-ornithine on stress markers and sleep quality in healthy workers. Nutrition Journal. 2014;13:53.

254. Moldofsky H, Harris HW, Archambault WT, Kwong T, Lederman S. Effects of bedtime very low dose cyclobenzaprine on symptoms and sleep physiology in patients with fibromyalgia syndrome: a double-blind randomized placebo-controlled study. Journal of Rheumatology. 2011;38(12):2653–63.

255. Monk TH, Buysse DJ, Carrier J, Billy BD, Rose LR. Effects of afternoon ‘siesta’ naps on sleep, alertness, performance, and circadian rhythms in the elderly. Sleep. 2001;24(6):680–7.

256. Monti JM, Alterwain P, Debellis J. Comparative double-blind clinical study of midazolam, methaqualone, and placebo in outpatients with slight-to-moderate insomnia. Current Therapeutic Research - Clinical and Experimental. 1987;41(4):437–43.

257. Moon KT. Improving insomnia with melatonin, magnesium, and zinc. American Family Physician. 2011;84(11):1293.

258. Morales-Delgado R, Camara-Lemarroy CR, Salinas-Martinez R, Gamez-Trevino D, Arredondo-Jaime A, Hernandez-Maldonado E, et al. A randomized placebo-controlled trial evaluating the effect of melatonin on sleep quality in patients with mild-moderate dementia. European Geriatric Medicine. 2018 Aug;9(4):449–54.

259. Morgan K, Dixon S, Mathers N, Thompson J, Tomeny M. Psychological treatment for insomnia in the management of long-term hypnotic drug use: a pragmatic randomised controlled trial. British Journal of General Practice. 2003;53(497):923–8.

260. Morin CM, Bastien C, Guay B, Radouco-Thomas M, Leblanc J, Vallieres A. Randomized clinical trial of supervised tapering and cognitive behavior therapy to facilitate benzodiazepine discontinuation in older adults with chronic insomnia. American Journal of Psychiatry. 2004;161(2):332–42.

261. Morin CM, Beaulieu-Bonneau S, Belanger L, Ivers H, Sanchez Ortuno M, Vallieres A, et al. Cognitive-behavior therapy singly and combined with medication for persistent insomnia: Impact on psychological and daytime functioning. Behaviour Research & Therapy. 2016;87:109–16.

262. Morin CM, Edinger JD, Beaulieu-Bonneau S, Ivers H, Krystal AD, Guay B, et al. Effectiveness of Sequential Psychological and Medication Therapies for Insomnia Disorder: A Randomized Clinical Trial. Vol. 08, JAMA Psychiatry. 2020. p. 08.

263. Morin CM, Koetter U, Bastien C, Ware JC, Wooten V. Valerian-hops combination and diphenhydramine for treating insomnia: a randomized placebo-controlled clinical trial. Sleep. 2005;28(11):1465–71.

264. Moss J, Tew GA, Copeland RJ, Stout M, Billings CG, Saxton JM, et al. Effects of a pragmatic lifestyle intervention for reducing body mass in obese adults with obstructive sleep apnoea: a randomised controlled trial. BioMed Research International. 2014;2014:102164.

265. Most EIS, Scheltens P, Van Someren EJW. Prevention of depression and sleep disturbances in elderly with memory-problems by activation of the biological clock with light - a randomized clinical trial. Trials [Internet]. 2010 Feb 23;11 (no pagination)(19). Available from: http://www.trialsjournal.com/content/11/1/19http://ovidsp.ovid.com/ovidweb.cgi?T=JS&CSC=Y&NEWS=N&PAGE=fulltext&D=emed11&AN=50827809https://trialsjournal.biomedcentral.com/track/pdf/10.1186/1745-6215-11-19 https://trialsjournal.biomedcentral.com/track/pdf/10.1186/1745-6215-11-19

266. Mun S, Lee S, Park K, Lee SJ, Koh BH, Baek Y. Effect of Traditional East Asian Medicinal herbal tea (HT002) on insomnia: a randomized controlled pilot study. Integrative Medicine Research. 2019 Mar;8(1):15–20.

267. Murawski B, Plotnikoff RC, Rayward AT, Oldmeadow C, Vandelanotte C, Brown WJ, et al. Efficacy of an m-Health Physical Activity and Sleep Health Intervention for Adults: A Randomized Waitlist-Controlled Trial. Vol. 57, American Journal of Preventive Medicine. 2019. p. 503–14.

268. Mysliwiec V, Neylan TC, Chiappetta L, Nofzinger EA. Effects of a forehead cooling

device in veterans with chronic insomnia disorder and co-morbid medical and psychiatric conditions: a pilot study. Vol. 22, Sleep & Breathing. 2020. p. 22.

269. Naismith SL, Pye J, Terpening Z, Lewis S, Bartlett D. 'Sleep Well, Think Well' Group Program for Mild Cognitive Impairment: A Randomized Controlled Pilot Study. Behavioral Sleep Medicine. 2018;1–12.

270. Nakamura Y, Lipschitz DL, Donaldson GW, Kida Y, Williams SL, Landward R, et al. Investigating Clinical Benefits of a Novel Sleep-Focused Mind-Body Program on Gulf War Illness Symptoms: A Randomized Controlled Trial. Psychosomatic Medicine. 2017;79(6):706–18.

271. Naraphong W. Effects of a culturally sensitive exercise program on fatigue, sleep, mood, and symptom distress among Thai women with breast cancer receiving adjuvant chemotherapy: A pilot randomized controlled trial. Dissertation Abstracts International: Section B: The Sciences and Engineering. 2014;75(2-B(E)):No Pagination Specified.

272. Norell-Clarke A, Jansson-Fröjmark M, Tillfors M, Holländare F, Engström I. Cognitive behavioural therapy for comorbid insomnia and depression: a randomised, controlled study. Sleep medicine. 2013;14:e99-.

273. Norell-Clarke A, Tillfors M, Jansson-Frojmark M, Hollandare F, Engstrom I. How does cognitive behavioral therapy for insomnia work? An investigation of cognitive processes and time in bed as outcomes and mediators in a sample with insomnia and depressive symptomatology. International Journal of Cognitive Therapy. 2017 Dec;10(4):304–29.

274. Norris ER, Karen B, Correll JR, Zemanek KJ, Lerman J, Primelo RA, et al. A double-blind, randomized, placebo-controlled trial of adjunctive ramelteon for the treatment of insomnia and mood stability in patients with euthymic bipolar disorder.

Journal of Affective Disorders. 2013;144(1–2):141–7.

275. Ochsner Margolies S. Efficacy of a cognitive-behavioral treatment for insomnia among Afghanistan and Iraq (OEF/OIF) veterans with PTSD. Dissertation Abstracts International: Section B: The Sciences and Engineering. 2012;73(4-B):2513.

276. Okajima I, Akitomi J, Kajiyama I, Ishii M, Murakami H, Yamaguchi M. Effects of a Tailored Brief Behavioral Therapy Application on Insomnia Severity and Social Disabilities Among Workers With Insomnia in Japan: A Randomized Clinical Trial. Vol. 3, JAMA Network Open. 2020. p. e202775.

277. Olson JA, Artenie DZ, Cyr M, Raz A, Lee V. Developing a light-based intervention to reduce fatigue and improve sleep in rapidly rotating shift workers. Vol. 37, Chronobiology International. 2020. p. 573–91.

278. Omvik S, Sivertsen B, Pallesen S, Bjorvatn B, Havik OE, Nordhus IH. Daytime functioning in older patients suffering from chronic insomnia: treatment outcome in a randomized controlled trial comparing CBT with Zopiclone. Behaviour Research & Therapy. 2008;46(5):623–41.

279. Ong JC, Dawson SC, Mundt JM, Moore C. Developing a cognitive behavioral therapy for hypersomnia using telehealth: a feasibility study. Vol. 16, Journal of Clinical Sleep Medicine. 2020. p. 2047–62.

280. Ong JC, Manber R, Segal Z, Xia Y, Shapiro S, Wyatt JK. A randomized controlled trial of mindfulness meditation for chronic insomnia. Sleep. 2014;37(9):1553–63.

281. Ong JC, Shapiro SL, Manber R. Mindfulness meditation and cognitive behavioral therapy for insomnia: a naturalistic 12-month follow-up. Explore: The Journal of Science & Healing. 2009;5(1):30–6.

282. Ong JC, Xia Y, Smith-Mason CE, Manber R. A randomized controlled trial of mindfulness meditation for chronic insomnia: Effects on daytime symptoms and cognitive-emotional arousal. Mindfulness. 2018 Dec;9(6):1702–12.

283. Palesh O, Scheiber C, Selland B, Packer M, Kesler S, Janelsins M, et al. Phase II RCT of brief behavioral therapy for cancer-related insomnia (BBT-CI): effects on quality of life (QOL) and heart rate variability (HRV). Supportive care in cancer. 2017;25(2):S52-.

284. Palesh O, Solomon N, Hofmeister E, Jo B, Shen H, Cassidy-Eagle E, et al. A novel approach to management of sleep-associated problems in patients with breast cancer (MOSAIC) during chemotherapy: A pilot study. Vol. 43, Sleep: Journal of Sleep and Sleep Disorders Research. 2020. p. 1–11.

285. Palmieri G, Contaldi P, Fogliame G. Evaluation of effectiveness and safety of a herbal compound in primary insomnia symptoms and sleep disturbances not related to medical or psychiatric causes. Nature & Science of Sleep. 2017;9:163–9.

286. Passos GS, Poyares D, Santana MG, D'Aurea CV, Youngstedt SD, Tufik S, et al. Effects of moderate aerobic exercise training on chronic primary insomnia. Sleep Medicine. 2011;12(10):1018–27.

287. Peles E, Hetzroni T, Bar-Hamburger R, Adelson M, Schreiber S. Melatonin for perceived sleep disturbances associated with benzodiazepine withdrawal among patients in methadone maintenance treatment: a double-blind randomized clinical trial. Addiction. 2007;102(12):1947–53.

288. Peoples A, Heckler C, Kamen C, Peppone L, Janelsins M, Mustian K, et al. Examining the benefits of cognitive behavioral therapy for insomnia (CBT-I) on depression in cancer survivors with insomnia. Supportive care in cancer. 2015;23(1 SUPPL. 1):S323-S324.

289. Peoples AR, Garland SN, Perlis ML, Savard J, Heckler CE, Kamen CS, et al. Effects of cognitive behavioral therapy for insomnia and armodafinil on quality of life in cancer survivors: a randomized placebo-controlled trial. Journal of cancer survivorship. 2017;11(3):401-409.

290. Peter L, Reindl R, Zauter S, Hillemacher T, Richter K. Effectiveness of an Online CBT-I Intervention and a Face-to-Face Treatment for Shift Work Sleep Disorder: A Comparison of Sleep Diary Data. Vol. 16, International Journal of Environmental Research & Public Health [Electronic Resource]. 2019. p. 24.

291. Pigeon WR, Funderburk J, Bishop TM, Crean HF. Brief cognitive behavioral therapy for insomnia delivered to depressed veterans receiving primary care services: A pilot study. Journal of Affective Disorders. 2017;217:105–11.

292. Pigeon WR, Funderburk JS, Cross W, Bishop TM, Crean HF. Brief CBT for insomnia delivered in primary care to patients endorsing suicidal ideation: a proof-of-concept randomized clinical trial. Vol. 9, Translational Behavioral Medicine. 2019. p. 1169–77.

293. Pillai V, Anderson JR, Cheng P, Bazan L, Bostock S, Espie CA, et al. The Anxiolytic Effects of Cognitive Behavior Therapy for Insomnia: Preliminary Results from a Web-delivered Protocol [Internet]. Vol. 2, Journal of Sleep Medicine & Disorders. 2015. Available from: http://ovidsp.ovid.com/ovidweb.cgi?T=JS&CSC=Y&NEWS=N&PAGE=fulltext&D=prem6&AN=32195356

294. Pinniger R, Thorsteinsson EB, Brown RF, McKinley P. Tango dance can reduce distress and insomnia in people with self-referred affective symptoms. American Journal of Dance Therapy. 2013 Jun;35(1):60–77.

295. Pollack M, Kinrys G, Krystal A, McCall WV, Roth T, Schaefer K, et al. Eszopiclone coadministered with escitalopram in patients with insomnia and comorbid generalized anxiety disorder. Archives of General Psychiatry. 2008;65(5):551–62.

296. Pruiksma KE, Cranston CC, Rhudy JL, Micol RL, Davis JL. Randomized controlled trial to dismantle exposure, relaxation, and rescripting therapy (ERRT) for trauma-related nightmares. Psychological Trauma:Theory, Pesearch, Practice and Policy. 2018;10(1):67–75.

297. Radhakishun FS, van den Bos J, van der Heijden BC, Roes KC, O'Hanlon JF. Mirtazapine effects on alertness and sleep in patients as recorded by interactive telecommunication during treatment with different dosing regimens. Journal of Clinical Psychopharmacology. 2000;20(5):531–7.

298. Rafii F, Ameri F, Haghani H, Ghobadi A. The effect of aromatherapy massage with lavender and chamomile oil on anxiety and sleep quality of patients with burns. Vol. 46, Burns. 2020. p. 164–71.

299. Rahimi A, Ahmadpanah M, Shamsaei F, Cheraghi F, Bahmani DS, Holsboer-Trachsler E, et al. Effect of adjuvant sleep hygiene psychoeducation and lorazepam on depression and sleep quality in patients with major depressive disorders: Results from a randomized three-arm intervention. Neuropsychiatric Disease and Treatment. 2016 Jun 22;12:1507–15.

300. Ranjbar M, Firoozabadi A, Salehi A, Ghorbanifar Z, Zarshenas MM, Sadeghniiat-Haghighi K, et al. Effects of Herbal combination (Melissa officinalis L. and Nepeta menthoides Boiss. & Buhse) on insomnia severity, anxiety and depression in insomniacs: Randomized placebo controlled trial. Integrative Medicine Research. 2018

Dec;7(4):328–32.

301. Raskind MA, Peskind ER, Hoff DJ, Hart KL, Holmes HA, Warren D, et al. A parallel group placebo controlled study of prazosin for trauma nightmares and sleep disturbance in combat veterans with post-traumatic stress disorder. Biological psychiatry. 2007;61(8):928-934.

302. Rastogi S, Tevaarwerk AJ, Sesto M, Van Remortel B, Date P, Gangnon R, et al. Effect of a technology-supported physical activity intervention on health-related quality of life, sleep, and processes of behavior change in cancer survivors: a randomized controlled trial [Internet]. Psycho-oncology. 2020. Available from: https://www.cochranelibrary.com/central/doi/10.1002/central/CN-02159235/full

303. Rayward AT, Murawski B, Duncan MJ, Holliday EG, Vandelanotte C, Brown WJ, et al. Efficacy of an m-Health Physical Activity and Sleep Intervention to Improve Sleep Quality in Middle-Aged Adults: The Refresh Study Randomized Controlled Trial. Vol. 54, Annals of Behavioral Medicine. 2020. p. 470–83.

304. Redeker NS, Conley S, Anderson G, Cline J, Andrews L, Mohsenin V, et al. Effects of Cognitive Behavioral Therapy for Insomnia on Sleep, Symptoms, Stress, and Autonomic Function Among Patients With Heart Failure. Behavioral Sleep Medicine. 2018;1–13.

305. Redeker NS, Jeon S, Andrews L, Cline J, Mohsenin V, Jacoby D. Effects of Cognitive Behavioral Therapy for Insomnia on Sleep-Related Cognitions Among Patients With Stable Heart Failure. Behavioral Sleep Medicine. 2019;17(3):342–54.

306. Rethorst CD, Sunderajan P, Greer TL, Grannemann BD, Nakonezny PA, Carmody TJ, et al. Does exercise improve self-reported sleep quality in non-remitted major depressive disorder? Psychological Medicine. 2013;43(4):699–709.

307. Reynolds CF 3rd, Buysse DJ, Miller MD, Pollock BG, Hall M, Mazumdar S. Paroxetine treatment of primary insomnia in older adults. American Journal of Geriatric Psychiatry. 2006;14(9):803–7.

308. Reynolds CF 3rd, Serody L, Okun ML, Hall M, Houck PR, Patrick S, et al. Protecting sleep, promoting health in later life: a randomized clinical trial. Psychosomatic Medicine. 2010;72(2):178–86.

309. Reynolds CF 3rd, Smith GS, Dew MA, Mulsant BH, Miller MD, Schlernitzauer M, et al. Accelerating symptom-reduction in late-life depression: a double-blind, randomized, placebo-controlled trial of sleep deprivation. American Journal of Geriatric Psychiatry. 2005;13(5):353–8.

310. Rezaei E, Behboodi Moghadam Z, Hagani H. The effect of sleep health behavioral education on the depression of pregnant women with sleep disorders: a randomized control trial. Iranian Red Crescent Medical Journal. 2015;17(1):e11420.

311. Roger M, Attali P, Coquelin JP. Multicenter, double-blind, controlled comparison of zolpidem and triazolam in elderly patients with insomnia. Clinical Therapeutics. 1993;15(1):127–36.

312. Rondaneli M, Opizzi A, Monteferario F, Antonielo N, Mani R, Klersy C. Efficacy of 8-weeks treatment with a food supplement (melatonin, magnesium, zinc conveyed by pear pulp) on quality of sleep and morning alertness in primary insomnia elderly: Double-blind, placebo-controlled clinical trial. [Italian]. Giornale di Gerontologia. 2011 Feb;59(1):46–56.

313. Roscoe JA, Garland SN, Heckler CE, Perlis ML, Peoples AR, Shayne M, et al. Randomized placebo-controlled trial of cognitive behavioral therapy and armodafinil for

insomnia after cancer treatment. Journal of Clinical Oncology. 2015;33(2):165–71.

314. Roth T, White D, Schmidt-Nowara W, Wesnes KA, Niebler G, Arora S, et al. Effects of armodafinil in the treatment of residual excessive sleepiness associated with obstructive sleep apnea/hypopnea syndrome: a 12-week, multicenter, double-blind, randomized, placebo-controlled study in nCPAP-adherent adults. Clinical Therapeutics. 2006;28(5):689–706.

315. Rutten S, Vriend C, Smit JH, Berendse HW, Hoogendoorn AW, van den Heuvel OA, et al. A double-blind randomized controlled trial to assess the effect of bright light therapy on depression in patients with Parkinson's disease. BMC psychiatry. 2016;16(1):355.

316. Sandberg O, Franklin KA, Bucht G, Eriksson S, Gustafson Y. Nasal continuous positive airway pressure in stroke patients with sleep apnoea: a randomized treatment study. European Respiratory Journal. 2001;18(4):630–4.

317. Sandlund C, Hetta J, Nilsson GH, Ekstedt M, Westman J. Impact of group treatment for insomnia on daytime symptomatology: Analyses from a randomized controlled trial in primary care. International Journal of Nursing Studies. 2018;85:126–35.

318. Sato D, Yoshinaga N, Nagai E, Hanaoka H, Sato Y, Shimizu E. Randomised controlled trial on the effect of internet-delivered computerised cognitive-behavioural therapy on patients with insomnia who remain symptomatic following hypnotics: a study protocol. BMJ Open. 2018;8(1):e018220.

319. Scharf MB. Feasibility of an every-other-night regimen in insomniac patients: Subjective hypnotic effectiveness of quazepam, triazolam, and placebo. The Journal of Clinical Psychiatry. 1993 Jan;54(1):33–8.

320. Schiller H, Soderstrom M, Lekander M, Rajaleid K, Kecklund G. A randomized

controlled intervention of workplace-based group cognitive behavioural therapy for insomnia: the moderating effect of chronic stress. Sleep. 2017;40:A137-.

321. Schmid C, Hansen K, Kroner-Borowik T, Steil R. Imagery Rescripting and Imaginal Exposure in Nightmare Disorder Compared to Positive Imagery: A Randomized Controlled Trial. Psychotherapy & Psychosomatics. 2021. p. 1–13.

322. Schwartz T, Nihalani N, Virk S, Jindal S, Costello A, Muldoon R, et al. A comparison of the effectiveness of two hypnotic agents for the treatment of insomnia. International Journal of Psychiatric Nursing Research. 2004;10(1):1146–50.

323. Scogin F, Lichstein K, DiNapoli EA, Woosley J, Thomas SJ, Larocca MA, et al. Effects of integrated telehealth-delivered cognitive-behavioral therapy for depression and insomnia in rural older adults. Journal of psychotherapy integration. 2018;28(3):292-309.

324. Sengul YS, Ozalevli S, Oztura I, Itil O, Baklan B. The effect of exercise on obstructive sleep apnea: a randomized and controlled trial. Sleep & Breathing. 2011;15(1):49–56.

325. Senturk A, Tekinsoy Kartin P. The Effect of Lavender Oil Application via Inhalation Pathway on Hemodialysis Patients’ Anxiety Level and Sleep Quality. Holistic Nursing Practice. 2018;32(6):324–35.

326. Serfaty MA, Osborne D, Buszewicz MJ, Blizard R, Raven PW. A randomized double-blind placebo-controlled trial of treatment as usual plus exogenous slow-release melatonin (6 mg) or placebo for sleep disturbance and depressed mood. International Clinical Psychopharmacology. 2010;25(3):132–42.

327. Shakya H, Wang D, Zhou K, Luo ZY, Dahal S, Zhou ZK. Prospective randomized controlled study on improving sleep quality and impact of zolpidem after total hip arthroplasty. Vol. 14, Journal of Orthopaedic Surgery. 2019. p. 289.

328. Shaw SH, Curson H, Coquelin JP. A double-blind, comparative study of zolpidem and placebo in the treatment of insomnia in elderly psychiatric in-patients. Journal of International Medical Research. 1992;20(2):150–61.

329. Short NA, Boffa JW, King S, Albanese BJ, Allan NP, Schmidt NB. A randomized clinical trial examining the effects of an anxiety sensitivity intervention on insomnia symptoms: Replication and extension. Behaviour Research & Therapy. 2017;99:108–16.

330. Shum A, Taylor BJ, Thayala J, Chan MF. The effects of sedative music on sleep quality of older community-dwelling adults in Singapore. Complementary Therapies in Medicine. 2014;22(1):49–56.

331. Siengsukon CF, Alshehri M, Williams C, Drerup M, Lynch S. Feasibility and treatment effect of cognitive behavioral therapy for insomnia in individuals with multiple sclerosis: A pilot randomized controlled trial. Vol. 40, Multiple Sclerosis and Related Disorders. 2020. p. 101958.

332. Smirne S, Cassano GB, Ferrillo F, Maggini C, Puca FM, Scarone S, et al. Brotizolam versus triazolam for the treatment of insomnia in anxious patients. Current Therapeutic Research - Clinical and Experimental. 1993;53(1):9–17.

333. Soares CN, Joffe H, Rubens R, Caron J, Roth T, Cohen L. Eszopiclone in patients with insomnia during perimenopause and early postmenopause: a randomized controlled trial. Obstetrics & Gynecology. 2006;108(6):1402–10.

334. Steinan M, Krane-Gartiser K, Langsrud K, Sand T, Kallestad H, Morken G. Cognitive behavioral theapy for insomnia in euthymic bipolar disorder: a randomized controlled trial. Bipolar disorders Conference: 10th international conference on bipolar disorder Miami beach, FL united states Conference start: 20130613 Conference end: 20130616

Conference publication: 15. 2013;15(suppl 1):101.

335. Sun J, Kang J, Wang P, Zeng H. Self-relaxation training can improve sleep quality and cognitive functions in the older: a one-year randomised controlled trial. Journal of Clinical Nursing. 2013;22(9–10):1270–80.

336. Sunnhed R, Hesser H, Andersson G, Carlbring P, Morin CM, Harvey AG, et al. Comparing internet-delivered cognitive therapy and behavior therapy with telephone support for insomnia disorder: A randomized controlled trial [Internet]. Vol. 43, Sleep. 2020. Available from: http://ovidsp.ovid.com/ovidweb.cgi?T=JS&CSC=Y&NEWS=N&PAGE=fulltext&D=emexc&AN=2010099651 http://resolver.shef.ac.uk/?http://find.shef.ac.uk/openurl/44SFD/44SFD_services_page?sid=OVID:embase&id=pmid:&id=doi:10.1093%2Fsleep%2Fzsz245&issn=0161-8105&isbn=&volume=43&issue=2&spage=&pages=&date=2020&title=Sleep&atitle=Comparing+internet-delivered+cognitive+therapy+and+behavior+therapy+with+telephone+support+for+insomnia+disorder%3A+A+randomized+controlled+trial&aulast=Sunnhed&pid=%3Cauthor%3ESunnhed+R.%3BHesser+H.%3BAndersson+G.%3BCarlbring+P.%3BMorin+C.M.%3BHarvey+A.G.%3BJansson-Frojmark+M.%3C%2Fauthor%3E%3CAN%3E2010099651%3C%2FAN%3E%3CDT%3EArticle%3C%2FDT%3E

337. Suresh Kumar PN, Andrade C, Bhakta SG, Singh NM. Melatonin in schizophrenic outpatients with insomnia: a double-blind, placebo-controlled study. Journal of Clinical Psychiatry. 2007;68(2):237–41.

338. Tahmasian M, Khazaie H, Ghadami M, Knight D, Emamian F. Insomnia treatment in the third trimester of pregnancy prevents postpartum depression: a randomized clinical trial.

Sleep medicine. 2013;14:e39.

339. Talbot LS, Maguen S, Metzler TJ, Schmitz M, McCaslin SE, Richards A, et al. Cognitive behavioral therapy for insomnia in posttraumatic stress disorder: a randomized controlled trial. Sleep. 2014;37(2):327-341.

340. Tanaka M, Kusaga M, Nyamathi AM, Tanaka K. Effects of Brief Cognitive Behavioral Therapy for Insomnia on Improving Depression Among Community-Dwelling Older Adults: a Randomized Controlled Comparative Study. Worldviews on evidence-based nursing / Sigma Theta Tau International, Honor Society of Nursing. 2019;16(1):78-86.

341. Tanaka M, Kusaga M, Nyamathi AM, Tanaka K. Effects of Brief Cognitive Behavioral Therapy for Insomnia on Improving Depression Among Community-Dwelling Older Adults: a Randomized Controlled Comparative Study. Vol. 16, Worldviews on evidence-based nursing / Sigma Theta Tau International, Honor Society of Nursing. 2019. p. 78-86.

342. Tang MF, Liou TH, Lin CC. Improving sleep quality for cancer patients: benefits of a home-based exercise intervention. Supportive Care in Cancer. 2010;18(10):1329–39.

343. Taylor DJ, Peterson AL, Pruiksma KE, Hale WJ, Young-McCaughan S, Wilkerson A, et al. Impact of cognitive behavioral therapy for insomnia disorder on sleep and comorbid symptoms in military personnel: A randomized clinical trial. Sleep: Journal of Sleep and Sleep Disorders Research. 2018 Jun;41(6):1–11.

344. Taylor HL, Rybarczyk B, Nay WT, Leszczyszyn D, Perrin PB, Auerbach SM. Effectiveness of a CBT intervention for persistent insomnia and hypnotic dependency in an outpatient psychiatry clinic. Sleep. 2014;37:A186.

345. Tek C, Palmese LB, Krystal AD, DeGeorge PC, Reutenauer EL, Guloksuz S. The impact

of eszopiclone insomnia treatment on cognition in schizophrenia: a double-blind, randomized, placebo-controlled trial. Biological psychiatry. 2014;75(9 SUPPL. 1):341S-342S.

346. Tsay SL, Cho YC, Chen ML. Acupressure and Transcutaneous Electrical Acupoint Stimulation in improving fatigue, sleep quality and depression in hemodialysis patients. American Journal of Chinese Medicine. 2004;32(3):407–16.

347. Tsay SL, Rong JR, Lin PF. Acupoints massage in improving the quality of sleep and quality of life in patients with end-stage renal disease. Journal of Advanced Nursing. 2003;42(2):134–42.

348. Tsoli S, Vasdekis S, Tigani X, Artemiadis A, Chrousos G, Darviri C. A novel cognitive behavioral treatment for patients with chronic insomnia: A pilot experimental study. Complementary Therapies in Medicine. 2018;37:61–3.

349. Tuomilehto HP, Seppa JM, Partinen MM, Peltonen M, Gylling H, Tuomilehto JO, et al. Lifestyle intervention with weight reduction: first-line treatment in mild obstructive sleep apnea. American Journal of Respiratory & Critical Care Medicine. 2009;179(4):320–7.

350. Uchimura N, Kamijo A, Takase T. Effects of eszopiclone on safety, subjective measures of efficacy, and quality of life in elderly and nonelderly Japanese patients with chronic insomnia, both with and without comorbid psychiatric disorders: a 24-week, randomized, double-blind study. Annals of General Psychiatry [Internet]. 2012 Jun 25;11 (no pagination)(15). Available from: http://www.annals-general-psychiatry.com/content/11/1/15http://ovidsp.ovid.com/ovidweb.cgi?T=JS&CSC=Y&NEWS=N&PAGE=fulltext&D=emed13&AN=52082711https://annals-general-psychiatry.biomedcentral.com/track/pdf/10.1186/1744-859X-11-15

https://annals-general-psychiatry.biomedcentral.com/track/pdf/10.1186/1744-859X-11-15

351. Vadas L, Bloch B, Reshef A, Kremer I, Haimov I. The effects of acupuncture on sleep quality and emotional measures among psychiatric ward inpatients. Journal of molecular neuroscience. 2012;48:S119.

352. van der Zweerde T, van Straten A, Effting M, Kyle SD, Lancee J. Does online insomnia treatment reduce depressive symptoms? A randomized controlled trial in individuals with both insomnia and depressive symptoms. Psychological medicine. 2019;49(3):501-509.

353. van Schagen AM, Lancee J, de Groot IW, Spoormaker VI, van den Bout J. Imagery rehearsal therapy in addition to treatment as usual for patients with diverse psychiatric diagnoses suffering from nightmares: a randomized controlled trial. Journal of Clinical Psychiatry. 2015;76(9):e1105-1113.

354. van Straten A, Emmelkamp J, de Wit J, Lancee J, Andersson G, van Someren EJ, et al. Guided Internet-delivered cognitive behavioural treatment for insomnia: a randomized trial. Psychological Medicine. 2014;44(7):1521–32.

355. Vedaa O, Hagatun S, Kallestad H, Pallesen S, Smith ORF, Thorndike FP, et al. Long-term effects of an unguided online cognitive behavioral therapy for chronic insomnia. Journal of Clinical Sleep Medicine. 2019 Jan 15;15(1):101–10.

356. Venter CP, Joubert PH, Stahmer SD. Zopliclone compared with triazolam in insomnia in geriatric patients. Current Therapeutic Research - Clinical and Experimental. 1986;40(6):1062–8.

357. Von Korff M, Vitiello MV, McCurry SM, Balderson BH, Moore AL, Baker LD, et al. Group interventions for co-morbid insomnia and osteoarthritis pain in primary care: the lifestyles cluster randomized trial design. Contemporary Clinical Trials.

2012;33(4):759–68.

358. Vuletic S, Bell KR, Jain S, Bush N, Temkin N, Fann JR, et al. Telephone Problem-Solving Treatment Improves Sleep Quality in Service Members With Combat-Related Mild Traumatic Brain Injury: Results From a Randomized Clinical Trial. Journal of Head Trauma Rehabilitation. 2016;31(2):147–57.

359. Wade AG, Crawford G, Ford I, McConnachie A, Nir T, Laudon M, et al. Prolonged release melatonin in the treatment of primary insomnia: evaluation of the age cut-off for short- and long-term response. Current Medical Research & Opinion. 2011;27(1):87–98.

360. Wagley JN, Rybarczyk B, Nay WT, Danish S, Lund HG. Effectiveness of abbreviated CBT for insomnia in psychiatric outpatients: sleep and depression outcomes. Journal of Clinical Psychology. 2013;69(10):1043–55.

361. Walsh JK, Hall-Porter JM, Griffin KS, Dodson ER, Forst EH, Curry DT, et al. Enhancing slow wave sleep with sodium oxybate reduces the behavioral and physiological impact of sleep loss. Sleep. 2010;33(9):1217–25.

362. Walsh JK, Krystal AD, Amato DA, Rubens R, Caron J, Wessel TC, et al. Nightly treatment of primary insomnia with eszopiclone for six months: effect on sleep, quality of life, and work limitations. Sleep. 2007;30(8):959–68.

363. Walsh JK, Roth T, Randazzo A, Erman M, Jamieson A, Scharf M, et al. Eight weeks of non-nightly use of zolpidem for primary insomnia. Sleep. 2000;23(8):1087–96.

364. Walsh JK, Soubrane C, Roth T. Efficacy and safety of zolpidem extended release in elderly primary insomnia patients. American Journal of Geriatric Psychiatry. 2008;16(1):44–57.

365. Wang CW, Kang J, Zhou JW, Hu YP, Li N. Effect of rolling needle therapy on quality of

life in the patient of non-organic chronic insomnia: a randomized controlled trial. Zhongguo Zhenjiu. 2006;26(7):461–5.

366. Wang F, Boros S. Effects of a pedometer-based walking intervention on young adults' sleep quality, stress and life satisfaction: randomized controlled trial. Vol. 24, Journal of bodywork and movement therapies. 2020. p. 286-292.

367. Wang HN, Tan QR, Zhang ZJ. Repetitive transcranial magnetic stimulation combined with venlafaxine for major depressive disorder with comorbid anxiety and insomnia: a controlled polysomnographic and diffusion MRI study. Sleep medicine. 2017;40:e362-.

368. Watanabe M, Miki E, Watanabe T, Katagiri M, Fukuda R, Urade Y. Effects of Intake of 3,5-Dihydroxy-4-Methoxybenzyl Alcohol (DHMBA) - Containing oyster extract drink on the status of stress, fatigue, and sleep - A randomized, double-blind, placebo-controlled, parallel-group study. [Japanese]. Japanese Pharmacology and Therapeutics. 2017;45(2):271–9.

369. Watanabe N. Clinical efficacy of psychotherapy targeted for insomnia in comorbid depression. Seishin Shinkeigaku Zasshi - Psychiatria et Neurologia Japonica. 2012;114(2):158–66.

370. Watanabe N. Cost-effectiveness of brief behavioral therapy for insomnia comorbid with depression: analysis of a randomized controlled trial. Psychosomatic medicine. 2014;76(3):A-90.

371. Watanabe N, Furukawa TA, Shimodera S, Morokuma I, Katsuki F, Fujita H, et al. Brief behavioral therapy for refractory insomnia in residual depression: an assessor-blind, randomized controlled trial. Journal of Clinical Psychiatry. 2011;72(12):1651–8.

372. Watanabe N, Furukawa T, Shimodera S, Morokuma I, Katsuki F, Fujita H, et al. Brief

behavioral therapy for refractory insomnia in residual depression: assessor-blind, randomized controlled trial. Sleep and biological rhythms. 2011;9(4):256-257.

373. Whitworth JW, Nosrat S, SantaBarbara NJ, Ciccolo JT. High intensity resistance training improves sleep quality and anxiety in individuals who screen positive for posttraumatic stress disorder: A randomized controlled feasibility trial. Mental Health and Physical Activity. 2019 Mar;16:43–9.

374. Wing YK, Lam SP, Zhang J, Li SX, Kwok A, Li C, et al. A brief integrated sleep-focused treatment for persistent sleep disturbances in residual depression: an assessorblind, parallel group, randomized controlled study. Journal of sleep research. 2016;25:186-.

375. Wong KY, Chung KF, Au CH. Low-Intensity Cognitive Behavioral Therapy for Insomnia as the Entry of the Stepped-Care Model in the Community: A Randomized Controlled Trial. Behavioral Sleep Medicine. 2020. p. 1–17.

376. Wu HC, Chen YH, Lai JN, Hwang JS, Wang JD. Improving sleep quality in climacteric women with insomnia: A randomized, head-to-head trial between Jia-Wei-Shiau-Yau San (JWSYS) and Suan-Zao-Ren Tang (SZRT). European Journal of Integrative Medicine. 2011 Sep;3(3):e143–51.

377. Xiu D, Fung YL, Lau BH, Wong DFK, Chan CHY, Ho RTH, et al. Comparing dyadic cognitive behavioral therapy (CBT) with dyadic integrative body-mind-spirit intervention (I-BMS) for Chinese family caregivers of lung cancer patients: a randomized controlled trial. Vol. 28, Supportive care in cancer. 2020. p. 1523-1533.

378. Yamamoto M, Sasaki N, Somemura H, Nakamura S, Kaneita Y, Uchiyama M, et al. Efficacy of sleep education program based on principles of cognitive behavioral therapy to alleviate workers’ distress. Sleep and Biological Rhythms. 2016;14(2):211–9.

379. Yang CL, Chen CH. Effectiveness of aerobic gymnastic exercise on stress, fatigue, and sleep quality during postpartum: A pilot randomized controlled trial. International Journal of Nursing Studies. 2018;77:1–7.

380. Yang XJ, Zhang YF, Liu J, Liu YZ, Lang Y, Wang YJ, et al. The efficacy of internet-based cognitive behavior therapy on blood pressure for comorbid hypertension and insomnia. [Chinese]. Medical Journal of Chinese People's Liberation Army. 2017;42(4):331–5.

381. Ye YY, Liu J, Li XJ, Liu YZ, Lang Y, Lin L, et al. The efficacy of internet-based cognitive behavioral therapy for insomnia. [Chinese]. Medical Journal of Chinese People's Liberation Army. 2016 Apr;41(4):307–11.

382. Yeung WF, Ho FYY, Chung KF, Zhang ZJ, Yu BYM, Suen LKP, et al. Self-administered acupressure for insomnia disorder: a pilot randomized controlled trial. Journal of Sleep Research. 2018 Apr;27(2):220–31.

383. Zachariades F. A CBT self-management approach for insomnia among people with chronic pain: A randomized controlled trial. Dissertation Abstracts International: Section B: The Sciences and Engineering. 2013;73(10-B(E)):No Pagination Specified.

384. Zhen XH, Xie H, Xu X. Psychotherapy intervention for the insomnia status in patients with secondary infertility. [Chinese]. Chinese Journal of Clinical Rehabilitation. 2005 Apr 28;9(16):33–5.

385. Zhou L, Tian Y, Yu J, Chen T, An F. Effect of at-home cognitive behavior therapy combined with nursing on revised piper fatigue scale, pittsburgh sleep quality index, self-rating anxiety scale and self-rating depression scale of ovarian cancer patients after chemotherapy. Vol. 13, International journal of clinical and experimental medicine. 2020.

p. 4227-4234.

386. Zollman FS, Larson EB, Wasek-Throm LK, Cyborski CM, Bode RK. Acupuncture for treatment of insomnia in patients with traumatic brain injury: a pilot intervention study. Journal of Head Trauma Rehabilitation. 2012;27(2):135–42.

387. Zuo X, Dong Z, Zhang P, Zhang P, Chang G, Xiang Q, et al. Effects of cognitive behavioral therapy on sleep disturbances and quality of life among adults with type 2 diabetes mellitus: A randomized controlled trial. Vol. 30, Nutrition Metabolism & Cardiovascular Diseases. 2020. p. 1980–8.
