# Supplementary material for: Improving sleep quality leads to better mental health: A meta-analysis of randomised controlled trials
Source: Sleep Med Rev. 2021 Dec;60:101556. doi: 10.1016/j.smrv.2021.101556 (PMC8651630; doi:10.1016/j.smrv.2021.101556)
Supplement: Supplementary file 3 — Multimedia component 3 [file mmc3.pdf]

Risk of bias

| Study | D1 | D2 | D3 | D4 | D5 | D6 | Overall |
|---|---|---|---|---|---|---|---|
| Alessi et al. (2016) (116) | + | + | - | + | + | + | + |
| Ashworth et al. (2015) (117) | - | - | X | - | X | - | X |
| Behrendt et al. (2020) (118) | + | + | X | - | + | + | - |
| Bergdahl et al. (2016) (119) | + | - | - | - | X | - | X |
| Blom et al. (2017) (120) | + | - | - | X | X | + | X |
| Cape et al. (2016) (121) | + | - | - | X | X | - | X |
| Casault et al. (2015) (122) | + | + | + | + | X | - | + |
| Chang et al. (2016) (123) | - | - | - | - | X | - | X |
| Chang et al. (2016) (124) | + | + | - | + | + | - | + |
| Chao et al. (2021) (125) | + | + | X | + | + | + | + |
| Chen et al. (2009) (126) | + | - | - | - | + | - | - |
| Chen et al. (2019) (127) | - | + | + | + | + | - | + |
| Cheng et al. (2019) (128) | + | - | - | - | - | + | - |
| Christensen et al. (2016) (129) | + | - | + | + | X | - | X |
| Chung et al. (2018) (130) | + | + | - | - | X | + | X |
| Currie et al. (2000) (131) | + | - | - | - | - | - | - |
| Edinger et al. (2005) (132) | - | - | - | - | X | - | X |
| Espie et al. (2008) (80) | + | - | X | - | + | - | - |
| Espie et al. (2014) (133) | + | + | - | + | X | + | + |
| Espie et al. (2019) (134) | + | + | - | + | X | + | X |
| Falloon et al. (2015) (135) | + | + | X | X | + | - | X |
| Felder et al. (2020) (136) | + | + | X | X | + | + | X |
| Freeman et al. (2015) (137) | + | + | X | + | + | + | + |
| Freeman et al. (2017) (138) | + | - | - | - | X | + | X |
| Garland et al. (2014) (139) | + | + | + | - | X | + | X |
| Garland et al. (2019) (140) | - | + | X | - | + | + | - |
| Germain et al (2012) (141) | + | - | X | + | X | - | X |
| Glozier et al. (2019) (142) | + | - | + | + | X | + | X |
| Ham et al. (2020) (143) | - | - | - | + | + | - | - |
| Ho et al. (2014) (144) | + | - | X | + | X | + | X |
| Irwin et al. (2014) (145) | + | - | - | + | + | + | - |
| Jansson-Frojmark et al. (2012) (146) | + | - | - | - | - | - | - |
| Jernelov et al. (2012) (147) | + | - | - | - | + | + | - |
| Jungquist et al. (2012) (148) | - | - | - | - | X | - | X |
| Kaldo, V et al. (2015) (149) | + | - | X | X | X | + | X |
| Kalmbach et al. (2019) (150) | - | - | X | + | + | + | - |
| Katofsky, V et al. (2012) (151) | - | - | - | - | X | - | X |
| Kyle et al. (2020) (152) | + | + | X | + | + | + | + |
| Lancee et al. (2012) (153) | + | - | X | - | - | + | - |
| Lancee et al. (2013) (154) | + | - | X | - | + | + | - |
| Lee et al. (2020) (155) | + | + | X | + | + | + | + |
| Lichstein et al. (2013) (156) | - | - | - | - | X | - | - |
| Martinez et al. (2014) (157) | + | - | - | + | X | - | X |
| McCrae et al. (2019) (158) | + | - | X | + | X | + | X |
| McCurry et al. (1998) (159) | - | - | - | - | - | - | - |
| Nguyen et al. (2017) (160) | + | - | X | + | + | + | - |
| Nguyen et al. (2019) (161) | + | + | - | + | + | + | + |
| Norell-Clarke et al. (2015) (162) | - | - | X | + | - | - | - |
| Park et al. (2015) (163) | - | - | - | - | - | - | - |
| Peoples et al. (2019) (164) | + | - | X | X | X | + | X |
| Raskind et al. (2013) (165) | - | - | + | + | - | - | - |
| Sadler et al. (2018) (166) | + | - | X | - | - | + | - |
| Sato et al. (2019) (167) | + | - | - | X | + | + | X |
| Savard et al. (2005) (168) | - | - | - | + | X | - | X |
| Schiller et al. (2018) (169) | + | - | - | - | X | - | X |
| Sheaves et al. (2017) (170) | + | + | - | - | + | - | - |
| Sheaves et al. (2019) (171) | + | + | - | + | X | - | X |
| Song et al. (2020) (172) | X | - | X | X | X | - | X |
| Tek et al. (2014) (173) | + | - | - | - | + | + | - |
| Thiart et al. (2015) (174) | X | - | X | - | + | + | - |
| Wagley (2010) (175) | - | - | - | - | - | - | - |
| Wen et al. (2018) (176) | + | + | - | + | X | - | X |
| Yeung et al. (2011) (177) | + | - | X | + | + | - | - |
| Zhang et al. (2020) (178) | + | - | X | + | + | + | - |
| Zhu et al. (2018) (179) | + | - | X | + | X | + | X |

D1: Random sequence generation
D2: Allocation concealment
D3: Blinding of participants and personnel
D4: Blinding of outcome assessment
D5: Incomplete outcome data
D6: Selective reporting

Judgement
X High
- Unclear
+ Low
